# Supplementary material for: A dominant role of transcriptional regulation during the evolution of C4 photosynthesis in Flaveria species
Source: Nat Commun. 2025 Feb 14;16:1643. doi: 10.1038/s41467-025-56901-y (PMC11828953; doi:10.1038/s41467-025-56901-y)
Supplement: Supplementary file 1 — Supplementary Information [file 41467_2025_56901_MOESM1_ESM.pdf]

Supplementary figures

A dominant role of transcriptional regulation during the evolution of C_4 photosynthesis in *Flaveria* species

Ming-Ju Amy Lyu[1#*], Huilong Du[2,3#], Hongyan Yao[4#], Zhiguo Zhang[5#], Genyun Chen[1], Yuhui Huang[1,6], Xiaoxiang Ni[1,6], Faming Chen[1,6], Yong-Yao Zhao[1,6], Qiming Tang[1,6], Fenfen Miao[1,6], Yanjie Wang[1,6], Yuhui Zhao[2], Hongwei Lu[2], Lu Fang[2], Qiang Gao[2], Yiying Qi[7], Qing Zhang[7], Jisen Zhang[7], Tao Yang[8], Xuean Cui[5], Chengzhi Liang[2,3*], Tiegang Lu[5*], Xin-Guang Zhu[1*]

[1] State Key Laboratory of Plant Molecular Genetics, Center of Excellence for Molecular Plant Sciences, Chinese Academy of Sciences, Shanghai, China, 200032
[2] State Key Laboratory of Plant Genomics, Institute of Genetics and Developmental Biology, Innovation Academy for Seed Design, Chinese Academy of Sciences, Beijing, China;
[3] University of Chinese Academy of Sciences, Beijing, China; School of Life Sciences, Institute of Life Sciences and Green Development, Hebei University, Baoding, China.
[4] State Key Laboratory of Genetic Engineering, School of Life Sciences, Fudan University, Shanghai 200438, China
[5] Biotechnology Research Institute/National Key Facility for Gene Resources and Gene Improvement, Chinese Academy of Agricultural Sciences, Beijing, 100081, China
[6] University of Chinese Academy of Sciences, Beijing 100049, China
[7] Center for Genomics and Biotechnology, Fujian Provincial Key Laboratory of Haixia Applied Plant Systems Biology, Key Laboratory of Sugarcane Biology and Genetic Breeding, National Engineering Research Center for Sugarcane, College of Life Sciences, Fujian Agriculture and Forestry University, Fuzhou, China
[8] China National GeneBank, Shenzhen, 518120, China.
[#] These authors contributed equally

* Correspondence: zhuxg@cemps.ac.cn (X.G.Z.), lutiegang@caas.cn (L.T.), cliang@genetics.ac.cn (C.L.), lvmj@cemps.ac.cn (M.J.L.)

a

Species	*F. robusta*	*F. sonorensis*	*F. linearis*	*F. ramosissima*	*F. trinervia*
**Photosynthetic type**	C_3	Type I C_3-C_4	Type I C_3-C_4	Type II C_3-C_4	C_4
**Genome size (GB)**	0.55	1.26	1.66	1.42	1.8
**Genome size estimated by flow cytometry (GB)**	0.45	1.2	1.86	1.62	1.65
**anchored to chromosome (%)**	92.2	92	91.2	94.3	93.4
**Contig N50 (MB)**	7.9	1.8	1.2	0.76	1.6
**GC content (%)**	32.87	36.06	37.45	37.3	37.85
**BUSCO%**	99.2	98.1	92.5	97	95.1
**Gene number**	35,875	37,028	38,652	34,029	32,915
**Average gene length (bp, intron+exon)**	3564.67	3670.95	3973.67	3971.15	3555.47
**Average Exons number per gene**	5.53	5.54	5.44	5.93	5.66

b

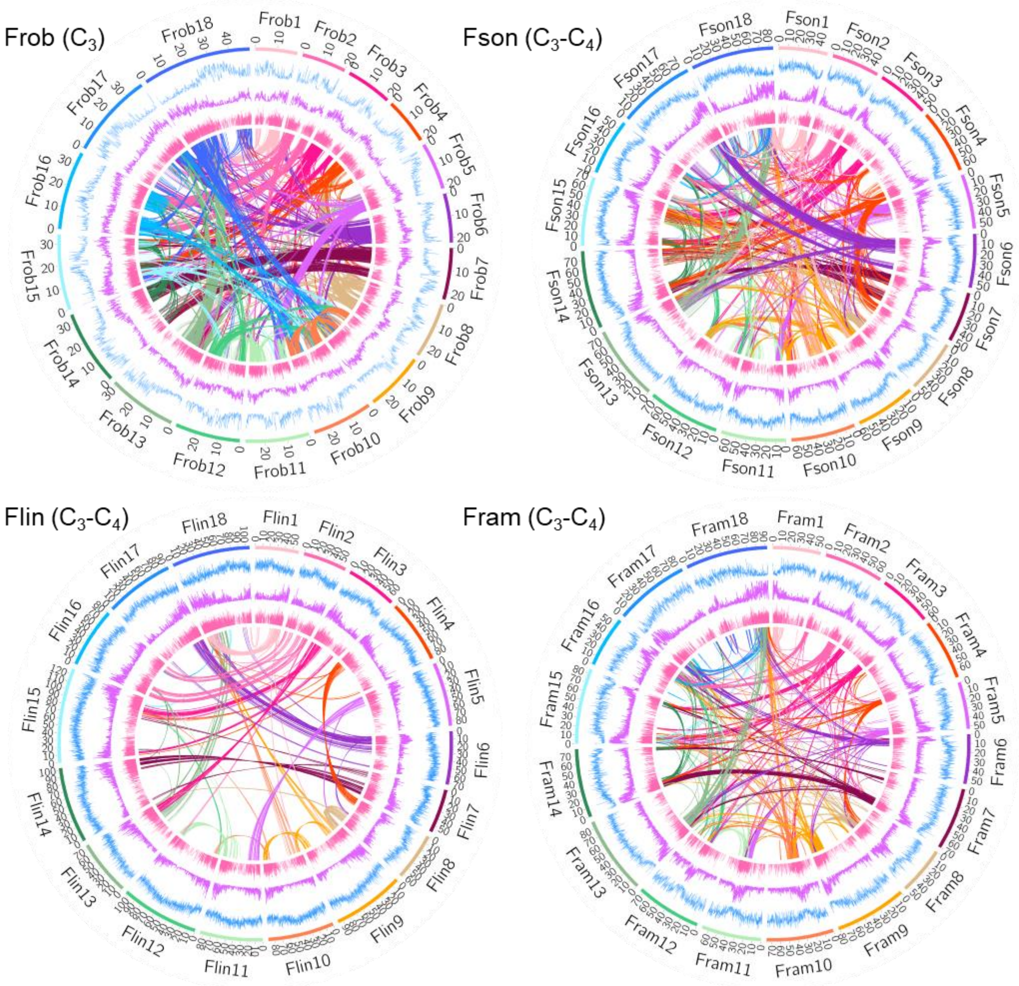

Supplementary Fig.1: Statistics of genome assemblies and genome features of five *Flaveria* species

(a) Statistics of genome assemblies and annotations. (b) The circular representation of pseudochromosomes. From outer to inner side: blue: LTR density per million base pair (Mb), purple:

exon density per Mb, pink: transcript abundance per gene in log10 TPM (transcript per million mapped reads). Lines in the inner circle represent links between synteny-selected paralogs. The genome features of the C_4 species *F. trinervia* is shown in Fig. 1c. (Abbreviations: Frob: *F. robusta*, Fson: *F. sonorensis*, Flin: *F. linearis*, Fram: *F. ramosissima*.)

a

b

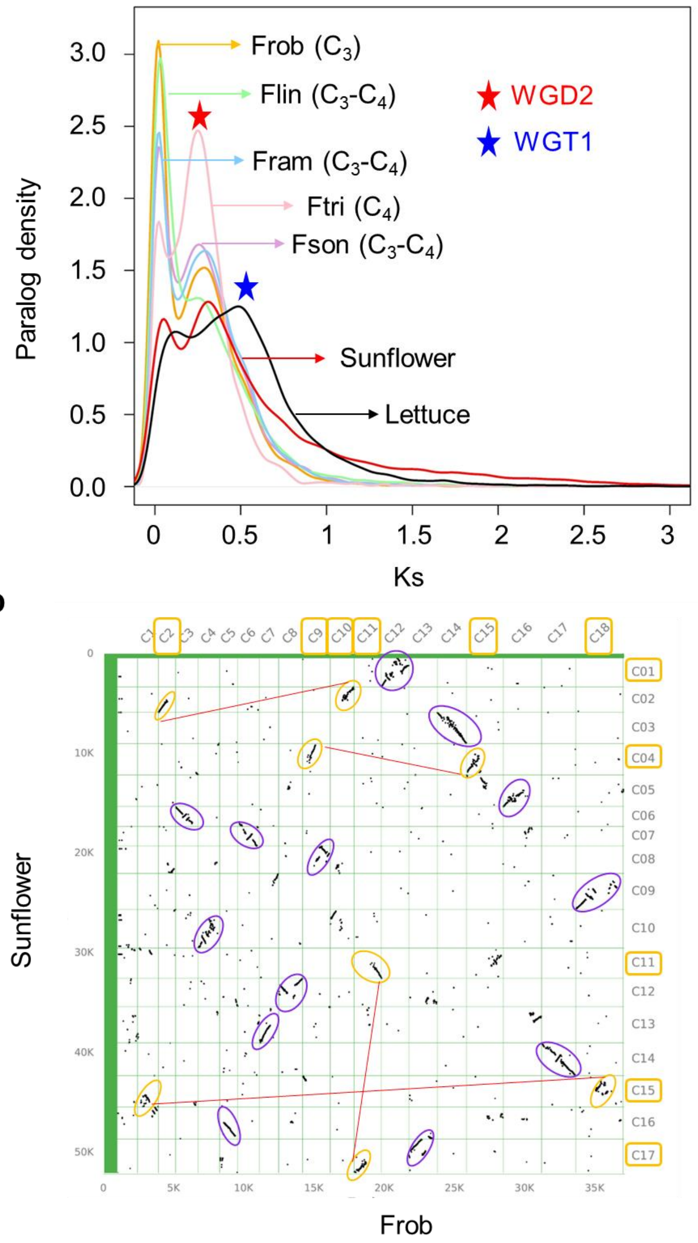

Supplementary Fig.2: *Flaveria* shared the whole genome duplication 2 event with sunflower

(a) Figure presents the distribution of Ks for paralogous gene pairs within and five *Flaveria* species, lettuce, and sunflower. Red star indicates the whole genome duplication 2 (WGD2) event and blue star indicates the whole genome triplication 1(WGT1) event. (b) Synteny analysis between Frob (C_3) and sunflower. The alignments of homologous regions between the Frob and sunflower chromosome were shown. Circles represent syntenic blocks between the two species, with purple circles indicating conserved chromosomes, and orange representing rearrangements, corresponding rearranged chromosomes are interconnected with red lines.

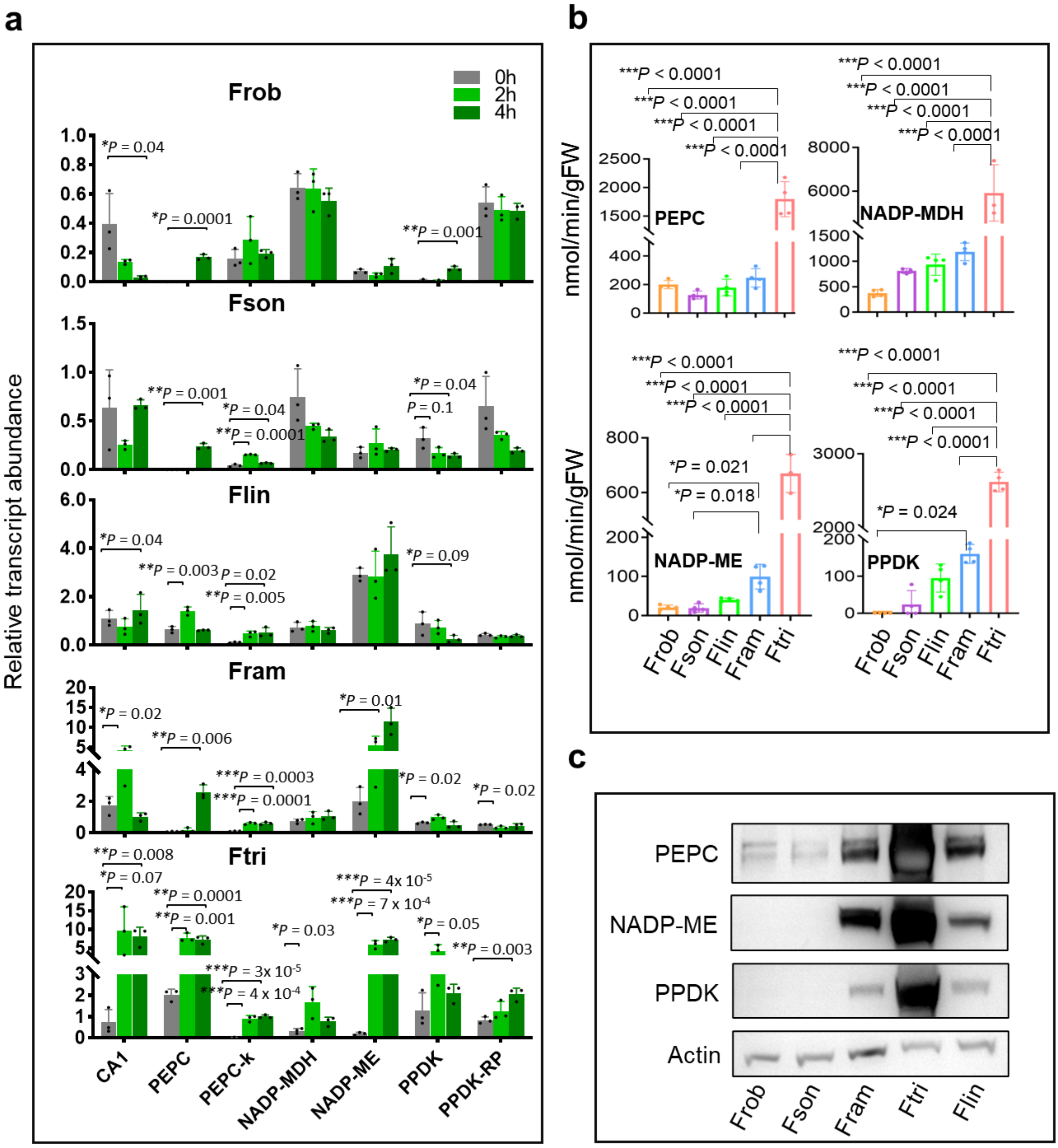

Supplementary Fig.3: Verification of functional copy of C_4 gene

(a) Real-time quantitative (qRT)-PCR was used to quantify the transcript abundance of C_4 enzymes in mature leaves after 0, 2 and 4h upon illumination. The relative transcript abundances are represented as mean ± SD (n>=3 biological replicates). (b) Enzyme activity of selected C_4 enzymes, Enzyme activities of four key C_4 enzymes PEPC, NADP-MDH, NADP-ME and PPDK were measured in leaf tissues of five *Flaveria* species. The activities are normalized to fresh weight and presented as mean ± SD (n>=3 biological replicates). Significance in (a) between 0h vs 2h and 0h vs 4h and (b) was determined using two-tailed Wilcoxon rank sum tests, P-values were adjusted with "BH" method (* P < 0.05, ** P < 0.01, *** P < 0.001). The activation of PPDK in Frob was unmeasurable due to very

low protein abundances. (c) Western blot analysis of selected C_4 enzymes. The analysis was conducted on leaf extracts prepared with the loading amount standardized per unit of fresh leaf weight, three plants from each species were analyzed. Actin was used as loading control. Due to the lack of a suitable *Flaveria* NADP-MDH antibody, the Western blot for NADP-MDH was not performed. (Abbreviations: *CA1*, *carbonic anhydrase 1*; *PEPC*, *phosphoenolpyruvate carboxylase*; *PEPC-k*: *PEPC kinase*; *NADP-MDH*, *NADP-dependent malate dehydrogenase*; *NADP-ME*, *NADP-dependent malic enzyme*; *PPDK*, *pyruvate/orthophosphate dikinase*; *PPDK-RP*, *PPDK regulatory protein*. Abbreviations for *Flaveria* species are the same as Fig.1). This experiment was conducted in 2 biological replicates and one representative result (c) was shown. Source data are provided as a Source Data file.

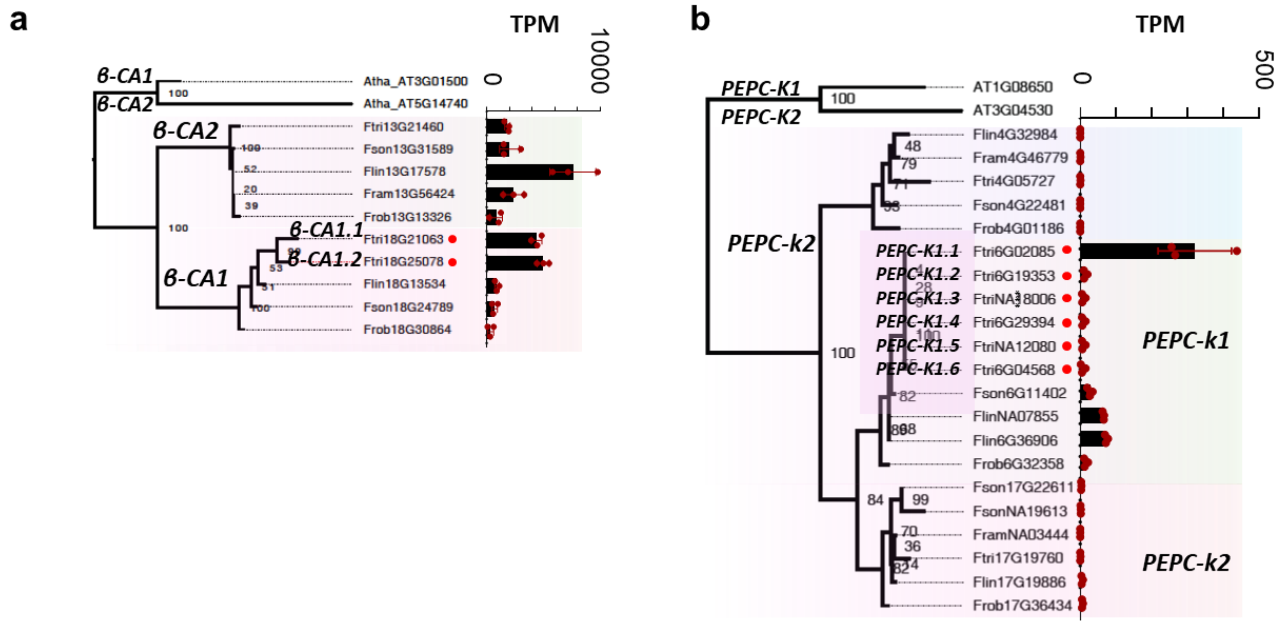

Supplementary Fig.4: C_4 versions of *CA* and *PEPC-k* show more copies in the C_4 species Ftri than in other *Flaveria* species resulting from gene duplications

(a) and (b) illustrate the gene trees of *CA* and *PEPC-k* respectively. Gene trees were constructed based alignment of protein sequences. Bootstrap scores were from 100 bootstrap samplings. Bars show transcript abundances in transcript per kilobase per million mapped reads (TPM). The bars show mean ± SD (n=3 biological replicates). C_4 versions of *CA* and *PEPC-k* are indicated with red circles. (Abbreviations: *CA1*: *carbonic anhydrase1*; *PEPC-k1*: *phosphoenolpyruvate carboxylase kinase1*.) Source data are provided as a Source Data file.

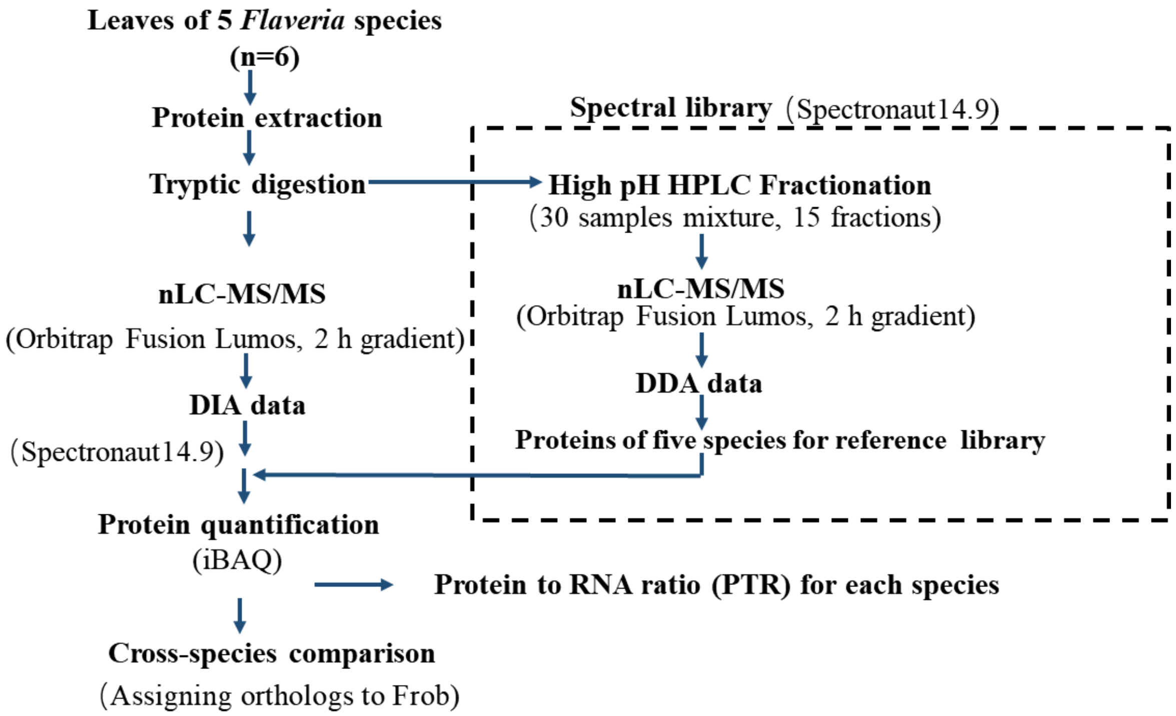

Supplementary Fig.5: Workflow for protein quantification and analysis in five *Flaveria* species

Total proteins were extracted from leaf tissues of five *Flaveria* species and subjected to tryptic digestion. Six biological replicates were used for each species. The resulting peptides were fractionated via high-pH HPLC, followed by LC-MS/MS analysis. For data-dependent acquisition (DDA), a reference library comprising the protein sequences of the five *Flaveria* species was used. Data-independent acquisition (DIA) scans were set to a resolution of 120,000. DIA data were analyzed using Spectronaut software. False discovery rate (FDR) was controlled at 1% for both peptide and protein levels. iBAQ values, quantified based on each species' own protein reference, were utilized for quantitative analysis. Protein-to-RNA ratios (PTR) were calculated for each species. To compare the PTRs between different orthologs across the five *Flaveria* species, genes from the other four species were assigned to the orthologs of Frob.

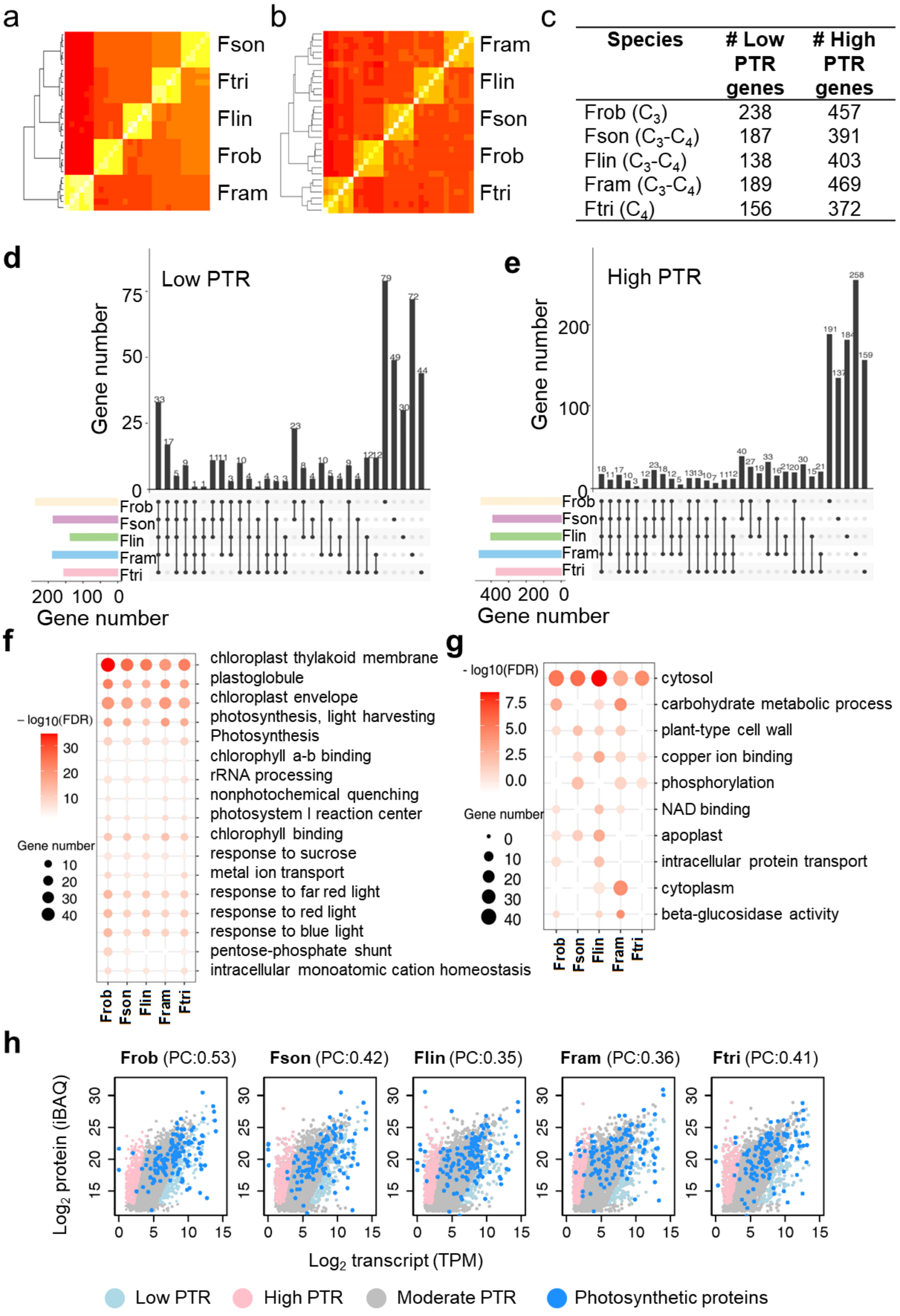

Supplementary Fig.6: Gene expression and protein abundances in five *Flaveria* species

(a) and (b) Heatmap showing the clustering of six replicates for each of the five *Flaveria* species based on gene expression levels (a) and protein abundances (b). Six RNA-seq samples from a previous report were used to study the gene expression profile along evolution. (c) The number of low and high protein-to-RNA ratio (PTR) genes. (d) and (e) Upset plots illustrates the intersection of low and high

PTR genes among the five *Flaveria* species, respectively. The x-axis represents the individual species, with the bar height indicating the number of low/high PTR genes in each species. The y-axis shows the number of intersected genes between different species combinations. Gene were assigned to orthologous to Frob for the other four species for (d) and (e). (f) and (g) Enriched gene ontology (GO) terms for low PTR and high PTR genes across five *Flaveria* species. (h) Scatter plots of protein abundance versus transcript abundance for the five *Flaveria* species. Low PTR, moderate PTR, high PTR, and photosynthetic genes (with gene ontology of GO:0015979) are labeled in different colors as indicated in the legend. The Pearson correlation (PC) between protein abundance and transcript abundance is shown in parentheses at the top of each panel.

a

Sample		Total Reads	Unique Mapped Reads	Multiple Mapped reads	Mapping Ratio
RNA-seq					
Frob	Rep 1	46, 729, 912	41, 523, 095 (88. 86%)	3, 846, 505 (8. 23%)	97.09%
Frob	Rep 2	49, 860, 724	44, 607, 561 (89. 46%)	3, 880, 411 (7. 78%)	97.25%
Ftri	Rep 1	43, 164， 414	39, 373, 809 (91. 22%)	2, 124, 477 (4. 92%)	96.14%
Ftri	Rep 2	41, 910, 674	38, 268, 697 (91. 31%)	1, 956, 667 (4. 67%)	95.98%
Ribo-seq					
Frob	Rep 1	6,166,898	1,283,449 (20.81%)	667,064 (10.82%)	31.63%
Frob	Rep 2	7,572,353	2,651,510 (35.02%)	1,007,866 (13.31%)	48.33%
Ftri	Rep 1	34,521,975	6,158,275 (17.84%)	1,791,245 (5.19%)	23.03%
Ftri	Rep 2	17,983,576	3,657,724 (20.34%)	1,206,263 (6.71%)	27.05%

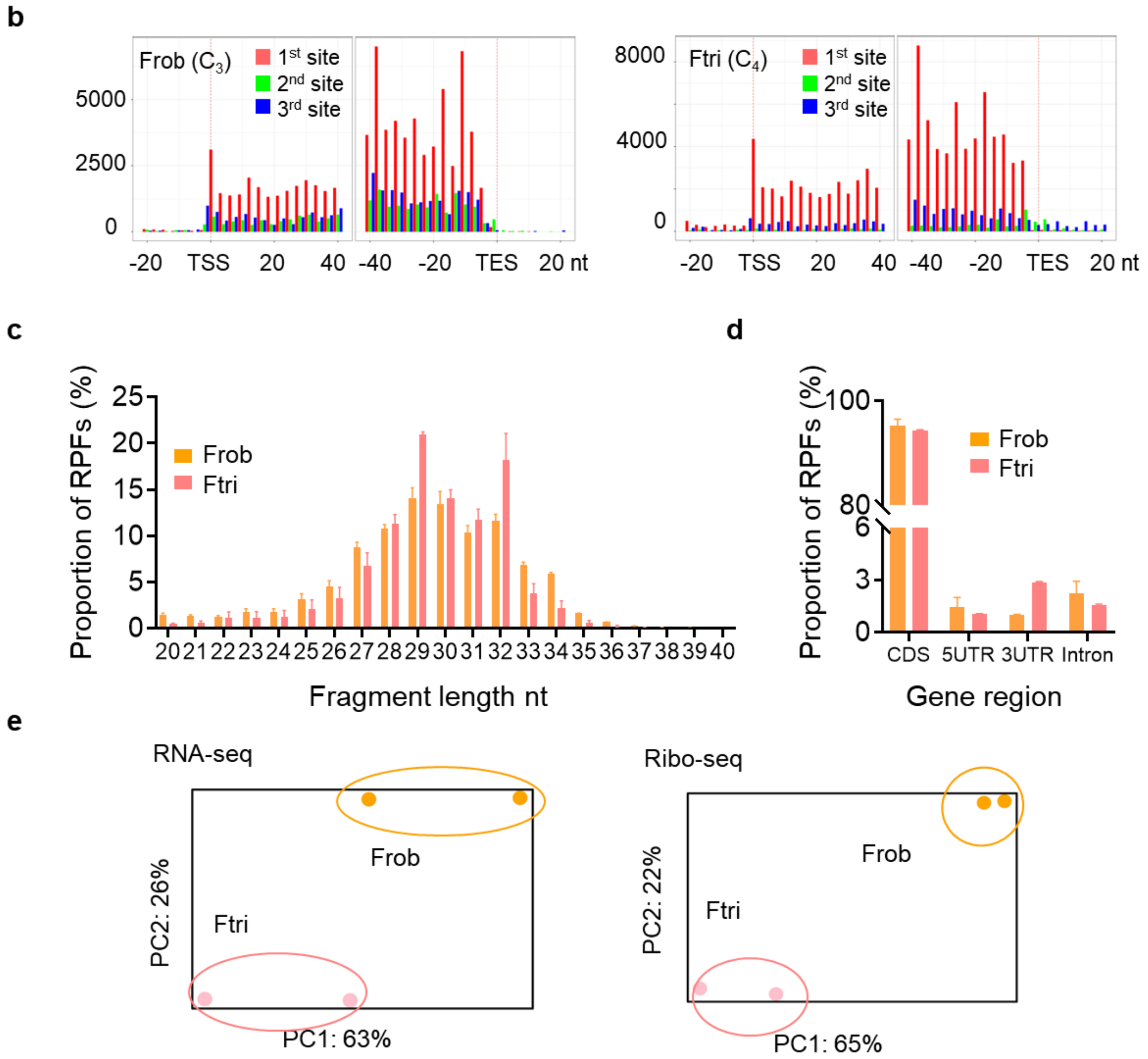

Supplementary Fig.7: The ribosome profiling for Frob and Ftri

(a) The statistic of ribosome profiling (Ribo-seq) for Frob and Ftri. (b) Three-nucleotide periodicity in Frob and Ftri near transcription start sites (TSS) and transcription end sites (TES). (c) The length distribution and relative abundance of the ribosome protected fragments (RPFs). (d) The distribution of RPFs coverage on CDS, 5' UTR, 3' UTR, and intron regions. RPFs from two corresponding replicates (n = 2 biological replicates) were combined for the analysis (c and d) in Frob and Ftri. (e) Principal component analysis based on gene expression from RNA-seq and Ribo-seq.

Frob (C_3)	Motif name	Ftri (C_4)	Motif name
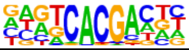	Ppas4 (q=0.05)	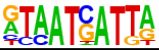	PHV(HB) (q=0.007)
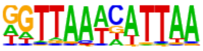	Hnf1 (q=0.049)	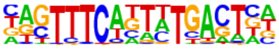	bZIP (q=0.006)
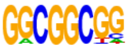	ERF104 (ERF) (q=0.04)	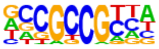	CRF10 (ERF) (q=0.05)
**Fson (C_3-C_4)**		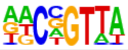	MYB65 (q=0.05)
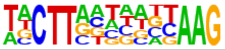	ANAC062 (NAC) (q=0.05)	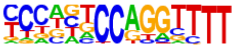	PRDM15 (q=0.05)
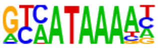	Cdx2 (homeobox) (q=0.04)	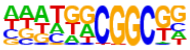	RAP26 (ERF) (q=0.05)
**Fram (C_3-C_4)**		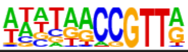	MYB3R4 (q=0.05)
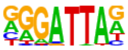	GSC (Homeobox) (q=0.05)	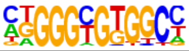	KLF6 (q=0.05)
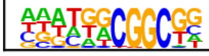	PR(NR) (q=0.05)	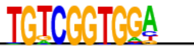	At1G77640 (ERF) (q=0.05)

Supplementary Fig.8: Enriched *cis*-regulatory elements of C_4 genes across five *Flaveria* species

The enriched motifs in the promoters (3 kb upstream of the start codon) of C_4 genes and their orthologous genes were identified to predict enriched motifs. The false discovery rate (q-values) for each motif is shown. For each species, the promoters of all the genes except for C_4 ones were used as background, p-values were adjusted with Benjamini and Hochberg correction (termed as q-values).

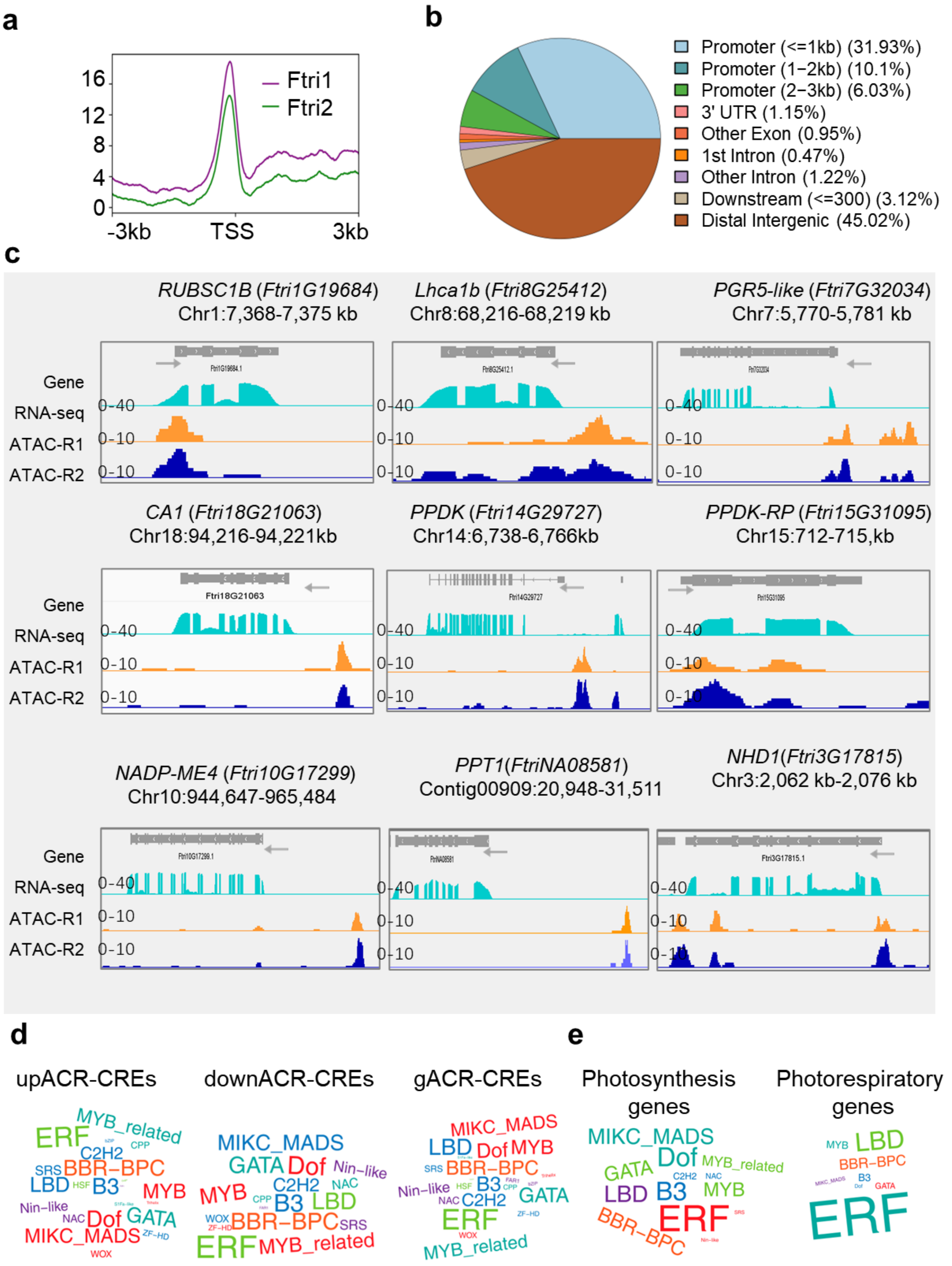

Supplementary Fig.9: Predicted *cis*-regulatory elements in the C4 species Ftri using ATAC-seq

(a) The plots show profiles of average ATAC-seq peak intensity from two independent biological replicates for all annotated genes near the transcription start sites (TSS). The x-axis represents the

distance from the TSS, y-axis depicts the normalized read count per million mapped reads. (b) The pie chart demonstrates the distribution of ATAC-seq peaks in relation to gene positions. (c) Genome browser tracks displaying accessible chromatin regions and transcript abundances (RNA-seq) of examples of photosynthesis genes and C_4 genes. (d) Word clouds show the log2-transformed frequency of enriched *cis*-regulatory elements (CREs) identified through Monte Carlo permutation testing (FDR<0.05) across three types of accessible chromatin regions (ACR-CREs), *i.e.*, genic (gACR-CREs: overlapping a gene), upstream (upACR-CREs: within 3kb upstream of the start codon of a gene) and downstream (down ACRs-CRES: within 3kb downstream of the stop codon of a gene). (e) Enriched ACR-CREs associated with photosynthetic genes and photorespiratory genes. (Abbreviations: *RUBSC1b*: *Rubisco small subunit 1b*; *Lhca1b*: *light-harvesting complex a 1b*; *PGR5-like*: *proton gradient regulation 5-like*; *CA1*: *carbonic anhydrase 1*; *PPDK*: *pyruvate orthophosphate dikinase; PPDK-RP: PPDK regulatory protein; NADP-ME4: NADP-dependent malic enzyme 4; PPT1: phosphate/phosphoenolpyruvate translocator 1*; *NHD1*: *sodium: hydrogen antiporter 1;* ATAC-seq: transposase-accessible chromatin using sequencing; ACR: accessible chromatin regions; CREs: *cis*-regulatory elements.)

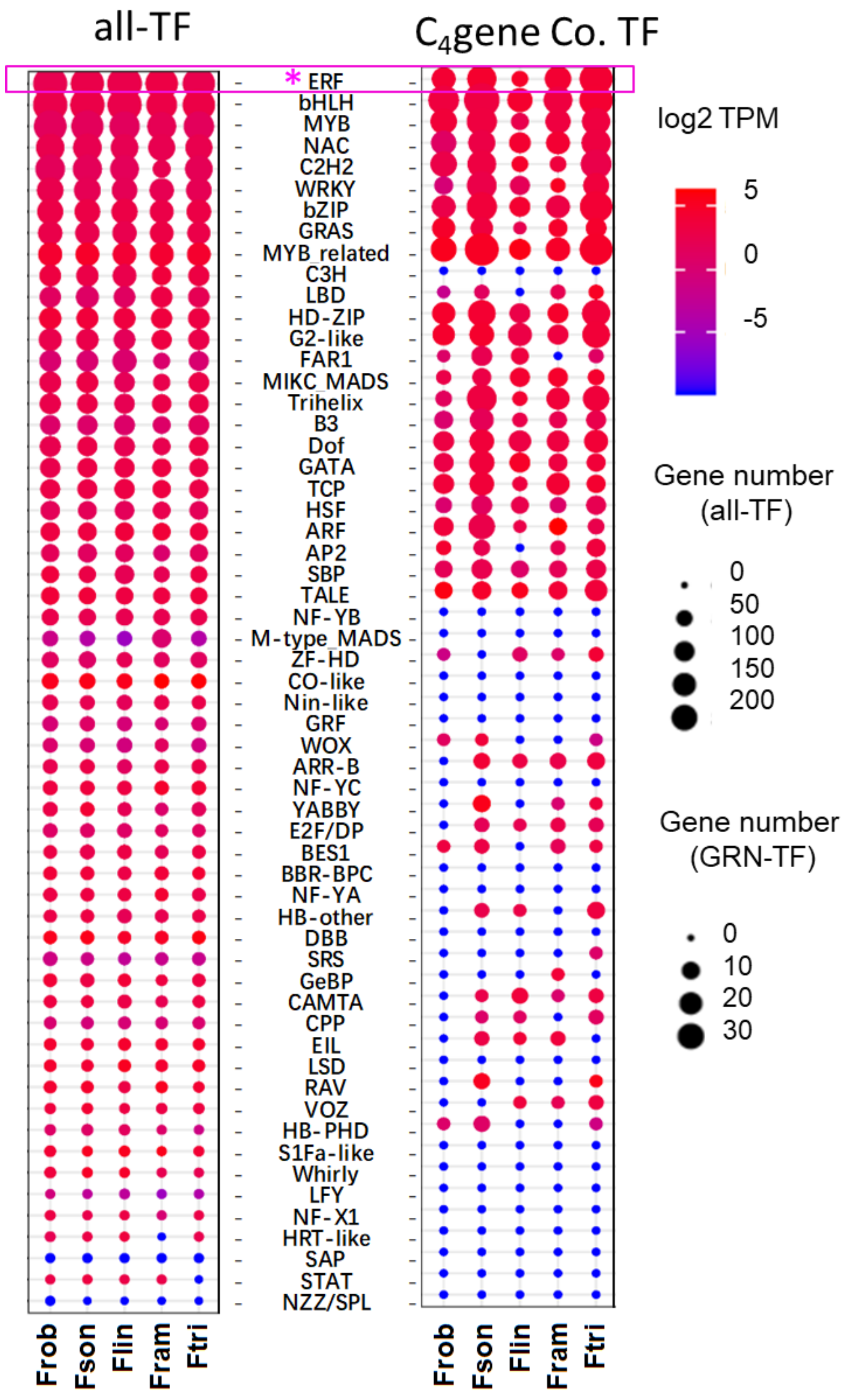

Supplementary Fig.10: The number of genes in each TF family and the transcript abundances of protein encoded by them

Heat maps show the gene number and transcript abundances in each TF family from all the annotated TFs (left panel) and from C_4GRN (right panel). The size of circle represents the number of genes, and the color represents the log2 transformed transcript abundances in transcript per million mapped reads

(TPM). (Abbreviations for *Flaveria* species are the same as Fig.1) Source data are provided as a Source Data file.
