# Supplementary material for: A dominant role of transcriptional regulation during the evolution of C4 photosynthesis in Flaveria species
Source: Nat Commun. 2025 Feb 14;16:1643. doi: 10.1038/s41467-025-56901-y (PMC11828953; doi:10.1038/s41467-025-56901-y)
Supplement: Supplementary file 3 — Description of Additional Supplementary Files [file 41467_2025_56901_MOESM3_ESM.pdf]

Description of Additional Supplementary Files

Supplementary Data 1: Estimation of genome sizes of five *Flaveria* species using Flow cytometry

Supplementary Data 2: Investigation of chromosome numbers using Fluorescence in situ hybridization assays

Supplementary Data 3: Estimation of genome assembly completeness

Supplementary Data 4: Comparison of protein-coding genes from Taniguchi's assemblies and our assemblies

Supplementary Data 5: Determination of functional copies of C4 genes

Supplementary Data 6: C4 version of *PEPC-k* was absent in Fram plant sequenced in this study

Supplementary Data 7: Verification of three copies of PEPC1 in the C4 species Ftri

Supplementary Data 8: Investigation of tandem duplications of C4 genes in other C4 species

Supplementary Data 9: Analysis of transposable elements and their effects on duplicated Ftri*PEPC1*

Supplementary Data 10: Comparison of transcript abundances based on RNA-seq data

Supplementary Data 11: Comparison of protein abundance based on proteomics

Supplementary Data 12: Comparison of protein-to-transcript ratio

Supplementary Data 13: Prediction of *cis*-regulatory elements using ATAC-seq 52

Supplementary Data 14: ERF *cis*-regulatory elements were abundant in photosynthesis related genes of different C4 species

Supplementary Data 15: Construction of gene regulatory networks
