# Supplementary material for: A dominant role of transcriptional regulation during the evolution of C4 photosynthesis in Flaveria species
Source: Nat Commun. 2025 Feb 14;16:1643. doi: 10.1038/s41467-025-56901-y (PMC11828953; doi:10.1038/s41467-025-56901-y)
Supplement: Supplementary file 4 — Supplementary data [file 41467_2025_56901_MOESM4_ESM.pdf]

Supplementary Data

Supplementary Data 1: Estimation of genome sizes of five *Flaveria* species using flow cytometry 2

Supplementary Data 2: Investigation of chromosome numbers using Fluorescence in situ hybridization assays 4

Supplementary Data 3: Estimation of genome assembly completeness 7

Supplementary Data 4: Comparison of protein-coding genes from Taniguchi's assemblies and our assemblies 12

Supplementary Data 5: Determination of functional copies of C_4 genes 18

Supplementary Data 6: C_4 version of *PEPC-k* was absent in Fram plant sequenced in this study 24

Supplementary Data 7: Verification of three copies of PEPC1 in the C_4 species Ftri 26

Supplementary Data 8: Investigation of tandem duplications of C_4 genes in other C_4 species 28

Supplementary Data 9: Analysis of transposable elements and their effects on duplicated Ftri*PEPC1* 30

Supplementary Data 10: Comparison of transcript abundances based on RNA-seq data 38

Supplementary Data 11: Comparison of protein abundance based on proteomics 41

Supplementary Data 12: Comparison of protein-to-transcript ratio 49

Supplementary Data 13: Prediction of *cis*-regulatory elements using ATAC-seq 52

Supplementary Data 14: ERF *cis*-regulatory elements were abundant in photosynthesis related genes of different C_4 species 55

Supplementary Data 15: Construction of gene regulatory networks 57

References 64

Supplementary Data 1: Estimation of genome sizes of five *Flaveria* species using flow cytometry

Methods

Genome size was estimated using flow-cytometry (FCM). For the FCM, *Solanum lycopersicum* (tomato, genome size 828 MB) was used as reference. Young leaves from multiple species were chopped with a blade soaked in nuclear isolation and staining buffer DAPI (NPE Analyzer, NPE 731085, USA). The suspension was filtered through a 40-lm nylon mesh (Sysmex Cell Trics). Samples were analyzed with a MD FACS Melody flow cytometer and data was analyzed using Kaluza software.

Results

The genome size of the five *Flaveria* species was estimated to be 0.45G for Frob, 1.2G for Fson, 1.86G for Flin, 1.62G for Fram and 1.65G for and Ftri (Supplementary data Fig. 1)

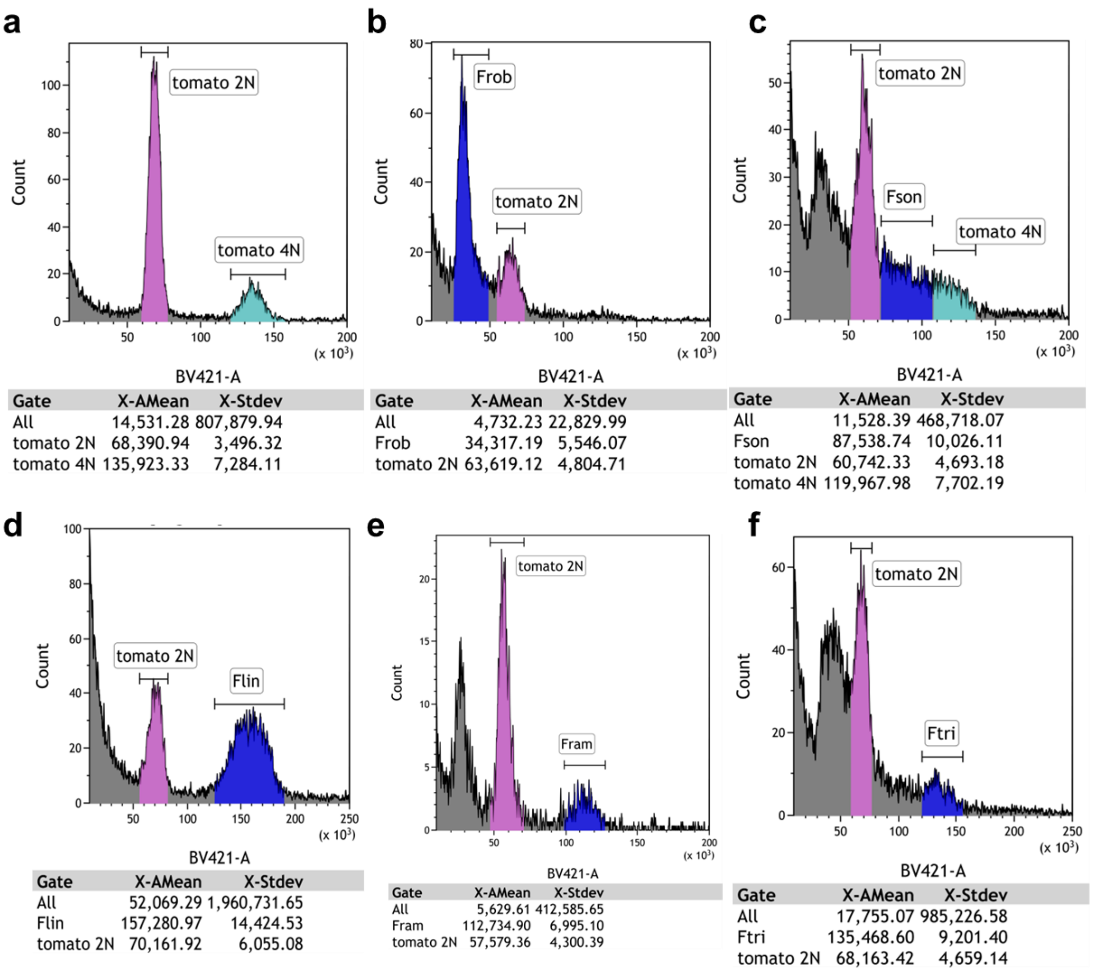

g

Tomato genome size (2n) in bp: 827,747,456			
Species	X-mean (Flaveria 2n)	X-mean (tomato 2n)	Estimated genome size (bp)
Frob(C_3)	34317.19	63619.12	447,039,887
Fson(C_3-C_4)	87538.74	60742.19	1,194,351,222
Flin(C_3-C_4)	157280.97	70161.92	1,857,791,309
Fram(C_3-C_4)	112734.9	57579.36	1,622,608,563
Ftri(C_4)	135468.6	68163.42	1,647,060,239

Supplementary data Fig. 1: Estimation of genome size using flow-cytometry

Solanum lycopersicum (tomato, genome size: 828 MB) was used as a reference. Samples were mixed for measurement as (a) only tomato, (b) Frob and tomato, (c) Fson and tomato, (d) Flin and tomato, (e) Fram and tomato and (f) Ftri and tomato. Young leaves from multiple species were chopped with a blade in nuclear isolation and the staining buffer DAPI. Estimated genome size of *Flaveria* species according to tomato are shown in (g). (Abbreviations: Frob: *F. robusta*; Fson: *F. sonorensis*; Flin: *F. linearis*; Fram: *F. ramosissima*; Ftri: *F. trinervia*)

Supplementary Data 2: Investigation of chromosome numbers using Fluorescence in situ hybridization assays

Methods

To investigate the chromosome number of Frob, Flin and Ftri, mitotic metaphase spreads were prepared from meristem root tip cells following a previously published method [1] with minor modifications. Briefly, root tips approximately 1 cm in length were cut and pretreated with nitrous oxide gas for 1-3 hours. The root tips were then fixed in ice-cold 90% acetic acid for 10 minutes and stored in 70% ethanol at - 20 °C. Root segments with actively dividing regions were excised and incubated in an enzyme mixture containing 1% (w/w) pectolyase Y-23 (Yakult Pharmaceutical, Tokyo, Japan) and 2% (w/w) cellulose Onozula R-10 (Yakult Pharmaceutical) for 30-50 minutes at 37 °C. After digestion, the root sections were washed twice in 75% ethanol. The root sections were fine-broken with a needle and treated in vortex machine at 2000 rpm for 20 seconds. The cells were collected by centrifugation and re-suspended in 30 ml 100% acetic acid to prepare a cell suspension.

The cell suspension (5–8 μL) was placed onto glass slides within a moist chamber and allowed to dry. The slides were then cross-linked using an ultraviolet cross-linking apparatus. Centromeric and telomeric repetitive sequences were used as probes for chromosome identification and counting. The slides were hybridized with these probes, followed by washing and counterstaining with DAPI (NPE Analyzer, NPE 731085, USA). Chromosome numbers of the three Flaveria species were determined by visualizing the slides under a microscope (Leica DM2500).

Results

The chromosome number was 2x18 in all the three analyzed species (Fig.1c), in line with previous reports. Additionally, Hi-C assembly supported the chromosome number of 18 for all five *Flaveria* species (Supplementary data Fig. 2). The synteny of the 18 chromosomes was conserved across the five *Flaveria* species; from 50% to 75% of protein coding genes being colinear between Frob and the other species

(Supplementary data table 1).

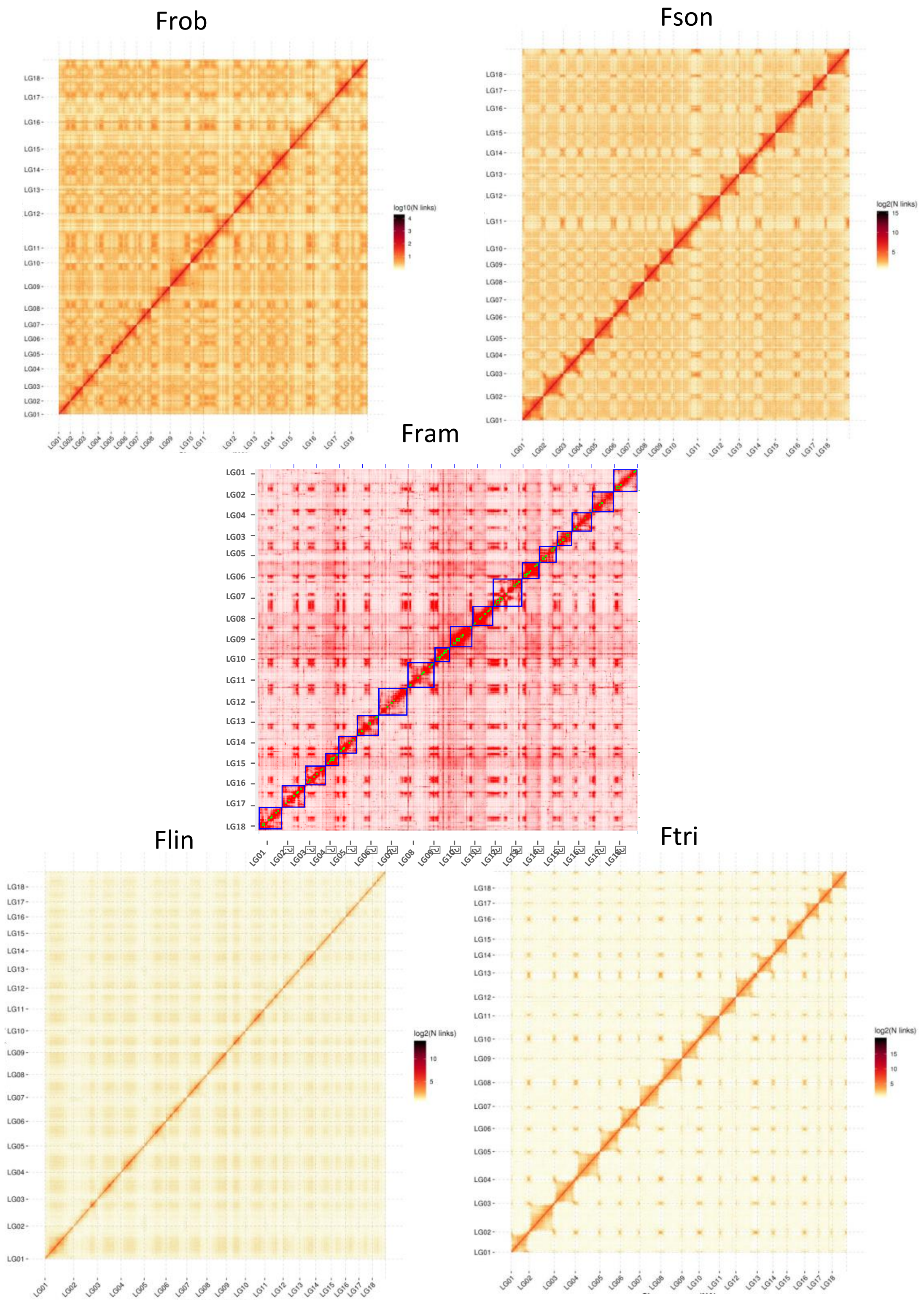

Supplementary data Fig. 2: Contact maps of the chromosomes of each *Flaveria* species

Normalized Hi-C contract matrix and assembled chromosomes number from 1 to 18 are shown in fragments of 100kb. X and Y lab show the chromosomes numbered from1 to 18.

Supplementary data table 1: The proportions of colinear genes between Frob and the other four sequenced *Flaveria* species

**Pair**	**% Collinearity**
Fson *vs*. Frob	72.46
Flin *vs*. Frob	50.15
Fram *vs*. Frob	51.96
Ftri *vs*. Frob	75.37

Supplementary Data 3: Estimation of genome assembly completeness

Methods

The completeness of genome assembly was estimated in three methods. First, the completeness of protein repertoire was estimated using Benchmarking Universal Single Copy Orthologues (BUSCO) (v3.0.2) [2] against a viridiplantae reference. Second, the completeness of genome assembly was estimated through RNA-seq mapping. RNA short reads sequencing was performed using the Illumina platform to obtain paired-ended reads with a length of 150 bp. RNA-seq reads from multiple experiments were mapped to the assembled genome sequences applying RSEM [3] in default parameters, where STAR (v2.7.3a) [4] was selected as the mapping tool. Third, the completeness of genome assembly was estimated through DNA-seq short reads mapping. DNA short reads sequencing was performed using the Illumina platform to obtain paired-ended reads with a length of 150 bp. DNA-seq reads were mapped to genome apply bowtie2 (v2.3.4.3) [5] in with "-k 10".

Results

BUSCO results demonstrated that 99.2% (Frob) to 92.5% (Flin) of BUSCO genes were covered (Supplementary data table 2). RNA-seq reads mapping results showed that a total of 3,290 million reads from five independent experiments were used for the five *Flaveria* species, and from 21 to 34 million reads were generated for each sample. For Frob, Fson, Fram and Ftri, between 92.0% and 97.6% of RNA-seq reads were mapped to corresponding genomes. Flin had a relatively lower mapping rates than other species, *i.e.*, around 86.7% to 90%. RNA-seq datasets from Flin from the high light experiment were generated using a different accession of Flin, which had relatively lower mapping rates, *i.e.*, around 80% (Supplementary data table 3). DNA-seq reads mapping results indicated that 1,110 million reads in total were used for the five *Flaveria* species, and between 170 and 324 million reads were generated for each species. From 95% to 98% paired-end reads were mapped to corresponding assembled genomes (Supplementary data table 4). Therefore, the above results suggested completeness and high quality of the genome assembly.

Supplementary data table 2: Statistics of BUSCO analysis

Species	Complete (%)	Complete (single) (%)	Complete (duplicated) (%)
**Frob** (C_3)	99.2	89.6	9.6
**Fson** (C_3-C_4)	98.1	89.6	8.5
**Flin** (C_3-C_4)	92.5	86.4	6.1
**Fram** (C_3-C_4)	95	88.4	6.6
**Ftri** (C_4)	95.1	91.1	4

Supplementary data table 3: Statistics of RNA-seq mapping

2-week low CO_2 experiment	# Total reads	Unique mapping ratio (%)	Multiple mapping ratio (%)	Total mapping ratio (%)
**Flin_2w_L_1**[@]	26106017	77.8	8.9	86.7
**Flin_2w_L_2**	33219838	79.6	9.8	89.4
**Flin_2w_L_3**	28760467	78.7	8.9	87.6
**Flin_2w_N_1**	31595547	80.5	9.5	90.0
**Flin_2w_N_2**	32903828	80.3	9.7	90.0
**Flin_2w_N_3**	33093266	80.6	9.7	90.3
**Fram_2w_L_1**	34936336	88.4	3.7	92.1
**Fram_2w_L_2**	32185750	88.8	3.5	92.3
**Fram_2w_L_3**	35172051	88.2	3.6	91.8
**Fram_2w_N_1**	15860328	88.3	3.7	92.0
**Fram_2w_N_2**	24661281	88.5	3.6	92.2
**Fram_2w_N_3**	34759285	88.4	3.8	92.2
**Frob_2w_L_1**	33887554	91.2	5.6	96.7
**Frob_2w_L_2**	36520872	91.6	5.5	97.1
**Frob_2w_L_3**	28938440	91.2	5.6	96.8
**Frob_2w_N_1**	33760952	91.0	5.8	96.8
**Frob_2w_N_2**	26874262	91.1	5.7	96.8
**Frob_2w_N_3**	34406245	91.3	5.8	97.1
**Fson_2w_L_1**	28557889	91.7	4.2	95.9
**Fson_2w_L_2**	26985392	91.7	4.1	95.8
**Fson_2w_L_3**	34615832	91.7	4.2	95.9
**Fson_2w_N_1**	28463981	91.5	4.3	95.8
**Fson_2w_N_2**	26500228	91.6	4.4	95.9
**Fson_2w_N_3**	32336172	91.8	4.3	96.1
**Ftri_2w_L_1**	35188793	92.2	4.3	96.5
**Ftri_2w_L_2**	29165653	89.0	7.4	96.4
**Ftri_2w_L_3**	32889783	92.3	4.4	96.6
**Ftri_2w_N_1**	31460531	92.2	4.0	96.2
**Ftri_2w_N_2**	32803186	92.5	4.1	96.6
**Ftri_2w_N_3**	29192442	92.6	3.9	96.5

**4-week low CO_2 experiment**	**# Total reads**	**Unique mapping ratio (%)**	**Multiple mapping ratio (%)**	**Total mapping ratio (%)**
**Flin_4w_L_1**	28,713,204	81.7	9.5	91.1
**Flin_4w_L_2**	23,778,224	81.5	9.4	90.9
**Flin_4w_L_3**	26,102,203	81.1	9.6	90.7
**Flin_4w_N_1**	24,809,359	80.9	9.6	90.5
**Flin_4w_N_2**	24,953,829	80.6	9.9	90.5
**Flin_4w_N_3**	23,095,900	80.3	9.9	90.1
**Fram_4w_L_1**	26,122,863	88.9	3.5	92.4
**Fram_4w_L_2**	19,480,458	89.3	3.6	92.8
**Fram_4w_L_3**	25,266,411	89.4	3.5	92.9
**Fram_4w_N_1**	22,702,731	89.3	3.5	92.8
**Fram_4w_N_2**	25,915,298	89.5	3.5	93.0
**Fram_4w_N_3**	25,468,944	89.5	3.5	93.0
**Frob_4w_L_1**	23,432,277	91.5	5.7	97.3
**Frob_4w_L_2**	24,386,859	90.9	5.7	96.6
**Frob_4w_L_3**	21,307,320	91.4	5.6	97.0
**Frob_4w_N_1**	28,024,652	91.2	5.9	97.1
**Frob_4w_N_2**	26,865,706	91.1	5.9	97.0
**Frob_4w_N_3**	25,369,429	90.9	5.9	96.8
**Fson_4w_L_1**	25,504,504	91.8	4.2	96.0
**Fson_4w_L_2**	16,723,354	91.9	4.3	96.2
**Fson_4w_L_3**	29,653,035	92.1	4.2	96.3
**Fson_4w_N_1**	27,538,862	91.3	4.5	95.8
**Fson_4w_N_2**	26,785,677	91.8	4.3	96.2
**Fson_4w_N_3**	29,476,651	91.8	4.3	96.2
**Ftri_4w_L_1**	28,423,320	92.0	4.7	96.8
**Ftri_4w_L_2**	27,234,665	92.4	4.7	97.0
**Ftri_4w_L_3**	26,384,930	91.0	4.6	95.6
**Ftri_4w_N_1**	28,143,172	92.3	4.5	96.8
**Ftri_4w_N_2**	23,287,687	92.4	4.4	96.8
**Ftri_4w_N_3**	22,794,859	92.6	4.5	97.1

**6-month low CO_2 experiment**	**# Total reads**	**Unique mapping ratio (%)**	**Multiple mapping ratio (%)**	**Total mapping ratio (%)**
**Fram_6m_L_1**	21847410	90.0	3.5	93.5
**Fram_6m_L_2**	20826620	89.7	3.6	93.2
**Fram_6m_L_3**	20952186	90.0	3.6	93.6
**Fram_6m_L_4**	20198206	89.7	3.5	93.3
**Fson_6m_L_1**	21858279	92.1	4.5	96.7
**Fson_6m_L_2**	24323857	92.4	4.3	96.8
**Fson_6m_L_3**	20875634	92.5	4.4	96.9
**Fson_6m_L_4**	24896827	91.3	4.5	95.8

ABA experiment	# Total reads	Unique mapping ratio (%)	Multiple mapping ratio (%)	Total mapping ratio (%)
**Fro-ABA-1**	22,810,323	92.4	3.7	96.0
**Fro-ABA-2**	26,320,179	92.4	4.4	96.8
**Fro-ABA-3**[#]	23,003,611	92.0	5.6	97.6
**Fro-Ctrl-1**	26,087,085	92.4	4.0	96.4
**Fro-Ctrl-2**	21,806,118	91.3	5.1	96.4
**Fro-Ctrl-3**	34,675,444	92.6	4.5	97.0
**Fso-ABA-1**	26,905,126	92.0	3.3	95.3
**Fso-ABA-2**	24,545,444	92.7	4.1	96.7
**Fso-ABA-3**	25,535,466	91.9	5.9	97.8
**Fso-Ctrl-1**	26,002,565	92.4	4.3	96.7
**Fso-Ctrl-2**	28,004,493	92.4	4.5	96.9
**Fso-Ctrl-3**	25,087,187	91.8	5.1	96.9
**Fra-ABA-1**	22,157,100	88.4	4.7	93.2
**Fra-ABA-3**	24,617,078	89.2	3.3	92.5
**Fra-ABA-4**	22,164,481	88.3	5.6	93.9
**Fra-Ctrl-1**	28,575,584	88.6	3.4	92.0
**Fra-Ctrl-2**	27,412,141	88.6	4.3	92.9
**Fra-Ctrl-3**	26,981,126	89.2	4.5	93.7
**Ftr-ABA-1**	24,748,259	92.4	3.5	95.9
**Ftr-ABA-2**	23,080,636	91.7	5.4	97.1
**Ftr-ABA-3**	25,120,473	91.2	6.0	97.2
**Ftr-Ctrl-1**	22,970,780	93.0	3.7	96.7
**Ftr-Ctrl-2**	23,937,914	92.4	3.7	96.1
**Ftr-Ctrl-3**	26,571,868	92.7	4.4	97.1
**Average**	26,830,957	89.6	5.1	94.8
**High-light experiment** [&]	**# Total reads**	**Unique mapping ratio (%)**	**Multiple mapping ratio (%)**	**Total mapping ratio (%)**
**Frob_HL_H_1**	32059692	75.9	6.8	82.7
**Frob_HL_H_2**	29783728	71.9	10.3	82.3
**Frob_HL_H_3**	31070734	74.9	6.9	81.9
**Frob_HL_L_1**	32903035	75.6	6.1	81.7
**Frob_HL_L_2**	30903465	77.4	6.8	84.2
**Frob_HL_L_3**	31645641	77.9	5.8	83.6
**Flin_HL_H_1**	33192028	72.0	9.2	81.2
**Flin_HL_H_2**	24233911	70.6	9.1	79.7
**Flin_HL_H_3**	22992824	69.2	8.9	78.1
**Flin_HL_L_1**	30945129	71.7	9.1	80.8
**Flin_HL_L_2**	20898845	71.2	9.1	80.3
**Flin_HL_L_3**	36695522	71.6	9.1	80.8
**Fram_HL_H_1**	33766904	75.4	4.5	80.0
**Fram_HL_H_2**	34883202	77.1	4.6	81.7

**Fram_HL_H_3**	34641349	76.3	4.7	81.0
**Fram_HL_L_1**	36517568	77.3	4.9	82.2
**Fram_HL_L_2**	40191972	75.6	4.8	80.4
**Fram_HL_L_3**	33976982	77.3	4.7	82.0
**Ftri_HL_H_1**	41616357	76.7	4.8	81.5
**Ftri_HL_H_2**	41633337	77.4	4.5	81.9
**Ftri_HL_H_3**	42552571	77.0	4.8	81.8
**Ftri_HL_L_1**	43200463	75.4	4.4	79.8
**Ftri_HL_L_2**	43456489	77.6	4.8	82.5
**Ftri_HL_L_3**	37944526	76.1	4.5	80.7

@ Sample with the lowest mapping ratio

Sample with the highest mapping ratio

& High light experiment was not included for computing average mapping ratio.

Supplementary data table 4: DNA-seq mapping statistics

**Species**	**# Total reads**	**# Mapped reads**	**Mapping ratio (%)**	**Properly mapped reads**	**Properly mapping ratio (%)**
**Frob** (C_3)	232,281,182	231,705,950	99.75	227,820,606	98.08
**Fson** (C_3-C_4)	189,408,029	188,479,930	99.51	184,350,835	97.33
**Flin** (C_3-C_4)	170,714,985	169,980,392	99.57	163,505,538	95.78
**Fram** (C_3-C_4)	324,792,136	323,557,926	99.62	309,332,030	95.24
**Ftri** (C_4)	202,443,727	201,073,807	99.32	191,310,924	94.50

Supplementary Data 4: Comparison of protein-coding genes from Taniguchi's assemblies and our assemblies

Methods

The published cDNA sequences from four *Flaveria* species obtained from Taniguchi's assembly [6]. Open reading frames (ORFs) of cDNA were predicted by applying OrfPredictor [7] using default parameters. Genes with an ORF of no less than 100 amino acids were retained for comparisons in both Taniguchi's assembly and our assembly. For Frob, genes from one assembly (either Taniguchi's assembly or our assembly) with counterparts found in another assembly were predicted using Blastp (v2.2.31+) [8] with an E-value threshold of 0.001 and a coverage threshold of 80%, where coverage is defined as the alignment length divided by the query sequence length. Protein identities were obtained from Blastp outputs.

Results

We compared the predicted protein-coding genes from our assembly with those from Taniguchi's assembly [6]. While more protein coding genes were reported in Taniguchi's assembly, approximately 30.7~35.6% of annotated genes were less than 100 amino acid in protein length [6], while the proportion of those in the five species reported here is ~4% (Supplementary data Fig. 3). Frob is the only species used in both and Taniguchi's assembly and ours. For Frob, 93.1% of the genes with protein length >=100 amino acids from Taniguchi's assembly were covered in our assembly (Blastp, E-value<0.001, coverage >=80%). In contrast, 80% of the genes with protein length >=100 amino acids in our assembly were also covered by Taniguchi's assembly (Supplementary data Fig. 4). Therefore, the annotated protein-coding genes in this study can be considered reliable.

We attempted to incorporate protein annotations from Taniguchi's assemblies into our evolutionary comparison study. However, we encountered challenges as several crucial C_4 enzymes, such as CA1, PEPC1, and NADP-ME4, were not annotated in the C_4 species *F. bidentis* (Supplementary data Fig. 5). Consequently, we

opted to exclude the comparison study involving Taniguchi's data in this analysis.

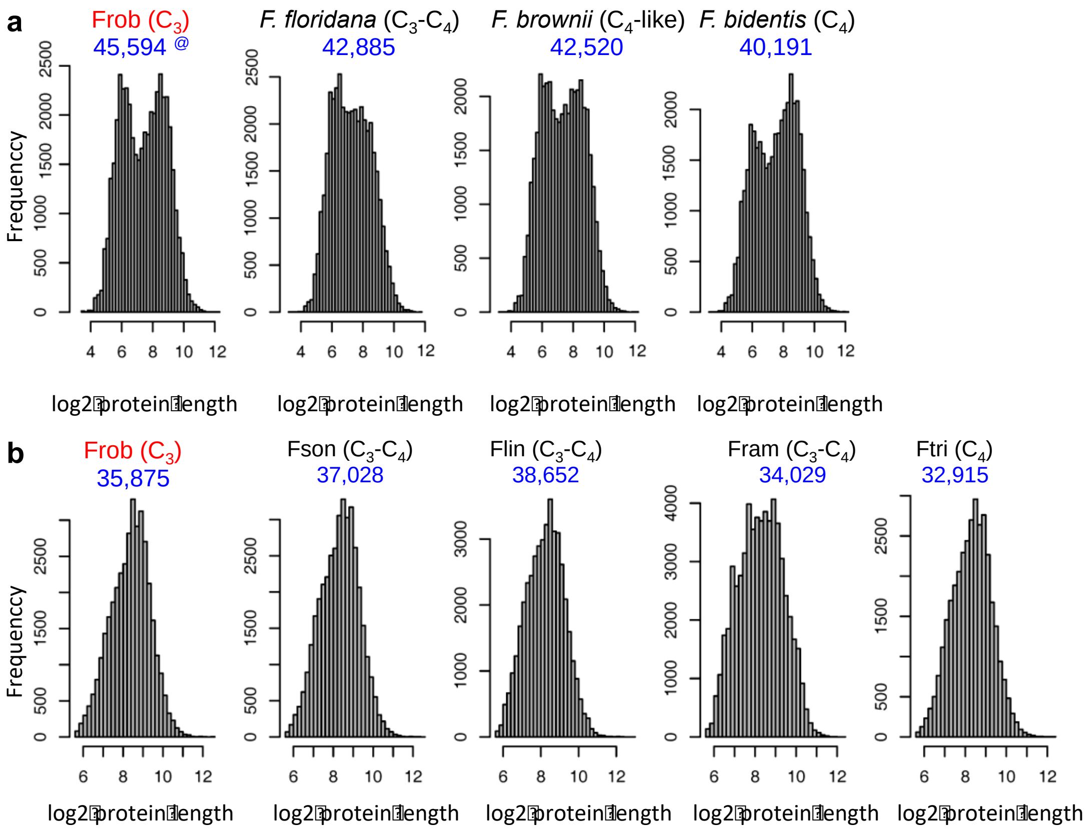

Supplementary data Fig. 3: Comparison of the protein length of predicted genes from our genome assembly as well as a published genome assembly

(a) Histograms showing the distribution protein length of the four *Flaveria* species from Taniguchi's report [6]. (b) Histograms indicate the distribution protein length of the five *Flaveria* species from our study. The number of annotated protein-coding genes are indicated under the name of species in blue font. Frob is the only common species between the two studies. (Note: @ The number of protein-coding genes as reported originally in Taniguchi's assembly are 46138/43016/42802/40631 for Frob/*F. floridana*/*F. brownii*/ *F. bidentis* respectively. The number of protein-coding genes on the histograms are the number of genes with open reading frames, therefore, the numbers are slightly lower than those originally reported)

a

F. robusta (C_3)	# Assembled gene	# Gene >=100 aa	# Gene >=100aa and with counterparts in the other assembly		
			Coverage >=70%	Coverage >=80%	Coverage >=90%
Taniguchi's assembly	45,594@	29,299	27,682 (94.5%)	27,290 (93.1%)	26,487 (90.0%)
Our assembly	35,875	34,388	29,260 (85.1%)	27, 665 (80.0%)	25,671 (74.6%)

b **c**

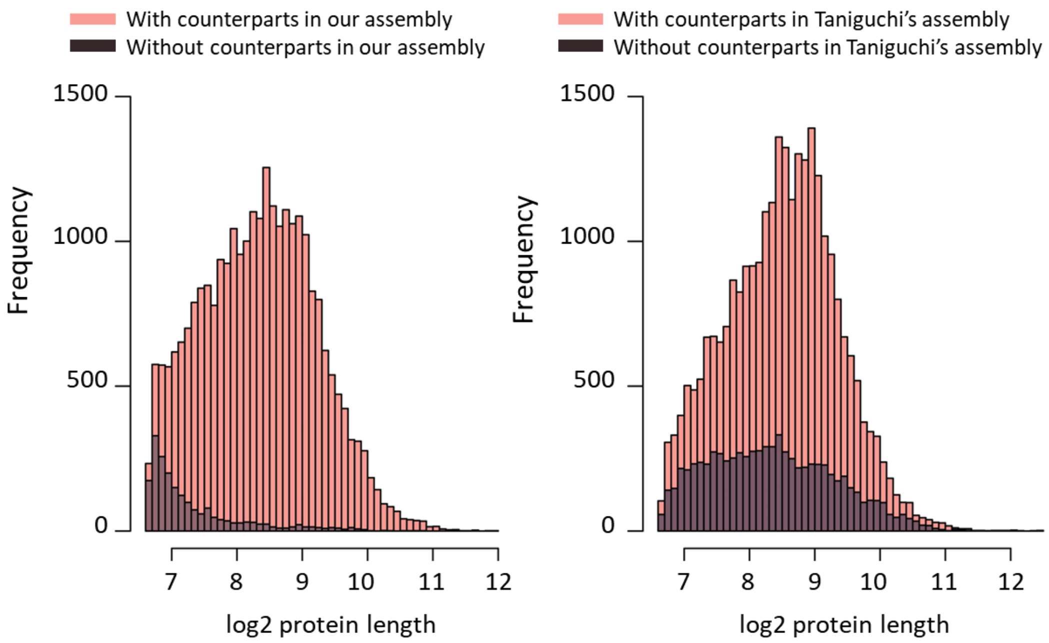

Supplementary data Fig. 4: Comparison of the annotated Frob protein from Taniguchi's assembly and our assembly

Genes with counterparts in another assembly (either ours or Taniguchi's) were predicted. (a) statistics on the number of annotated protein-coding genes and those with counterparts in the other assembly. The table includes data for both assemblies, with the number of genes having ⩾100 amino acids and the corresponding percentages of genes with counterparts in the other assembly at coverage thresholds of 70%, 80%, and 90%. (b) shows the distribution of protein lengths for genes from Taniguchi's assembly with (pink) and without (grey) counterparts in our assembly when considering a coverage threshold of 80%. (c) presents the distribution of protein lengths for genes from our assembly with (pink) and without (grey) orthologs in Taniguchi's assembly when applying a coverage threshold of 80%. (Note: @ The number of protein-coding gene reported originally in Taniguchi's assembly [6] is 46,138.)

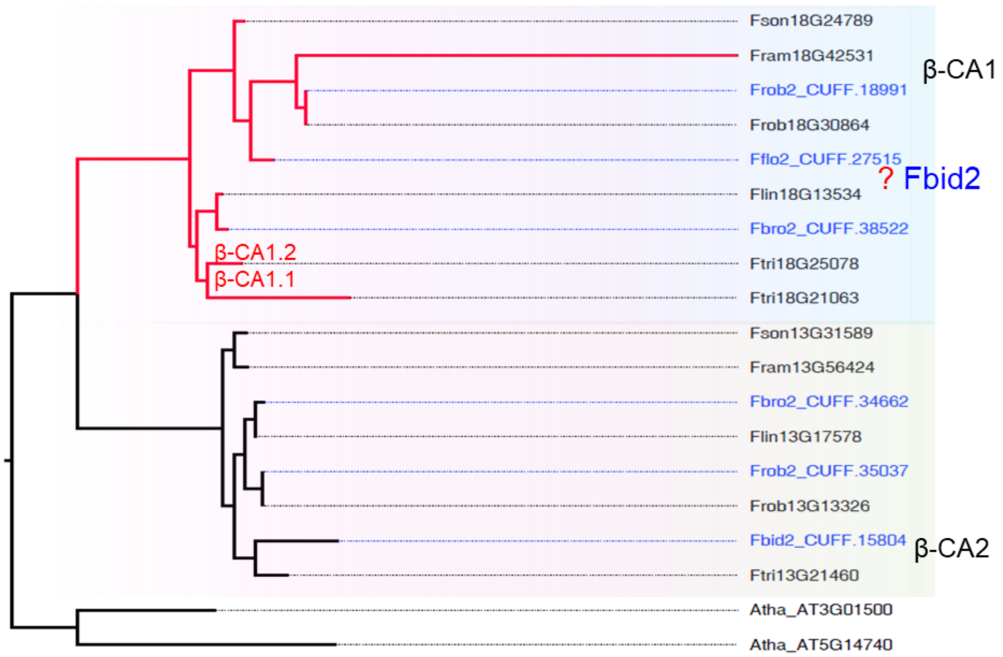

● From published assemblies: Frob2 (C_3), Fflo2 (C_3-C_4), Fbro2 (C_4-like), Fbid2 (C_4)
● From our assemblies: Frob (C_3), Fson (C_3-C_4), Fram (C_3-C_4), Ftri (C_4)

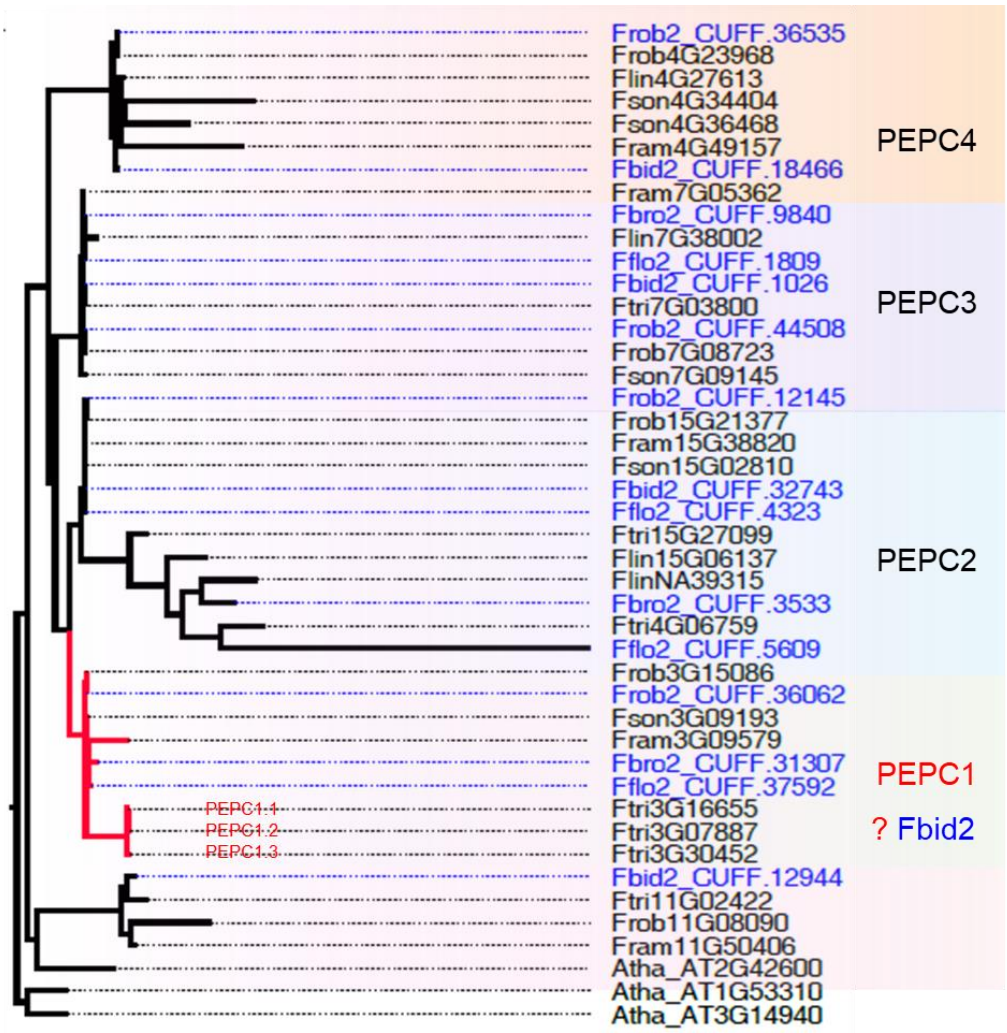

● From published assemblies: Frob2 (C_3), Fflo2 (C_3-C_4), Fbro2 (C_4-like), Fbid2 (C_4)

● From our assemblies: Frob (C_3), Fson (C_3-C_4), Fram (C_3-C_4), Ftri (C_4)

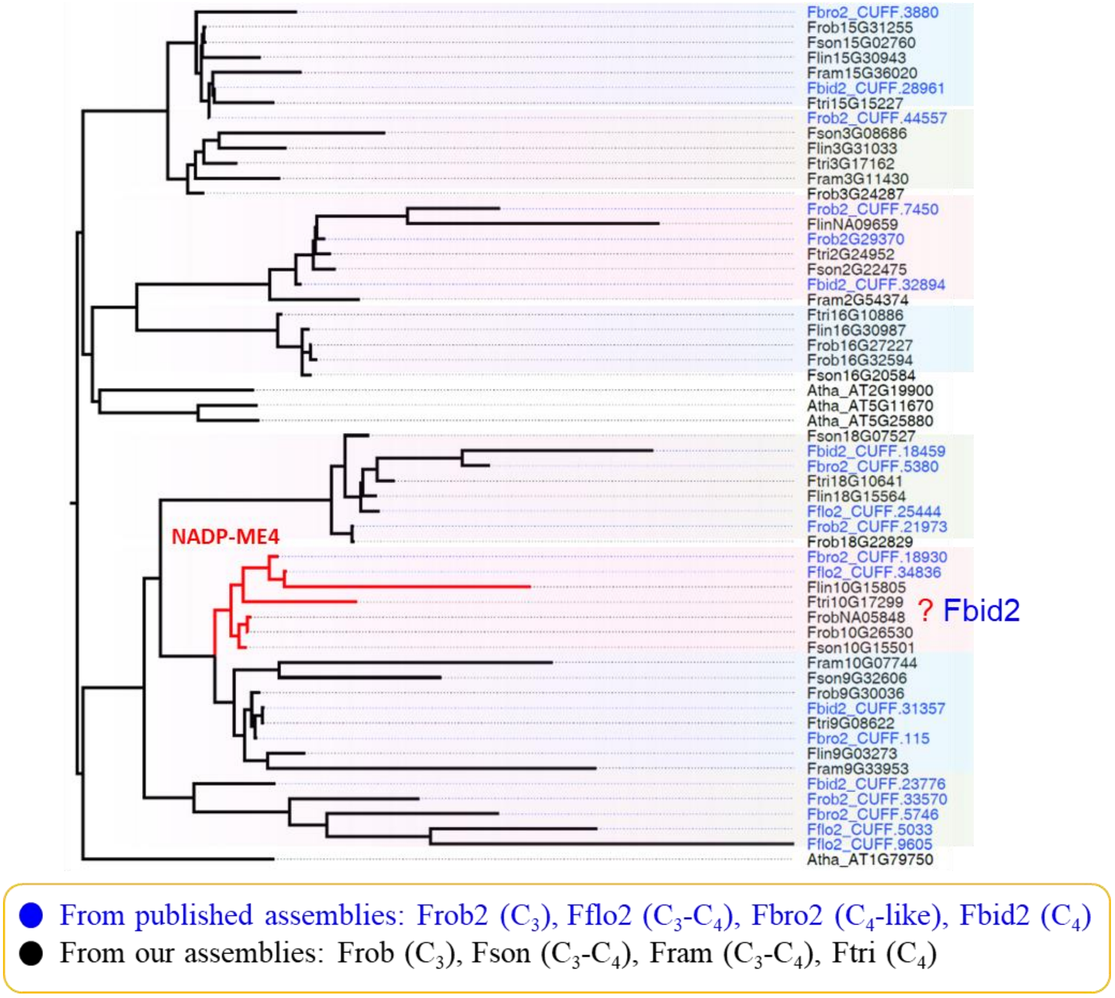

Supplementary data Fig. 5: The C_4 copies of CA1, PEPC1 and NADP-ME4 were not annotated in *F. bidentis* from published assembly

The phylogenetic tree of *PEPC* was constructed based on protein sequences. Sequences of Frob2, Fflo2, Fbro2 and Fbid2 (labeled in blue font on the gene tree) are from Taniguchi's assembly, those of Frob, Fson, Fram and Ftri (labeled in black font) are from our assembly. *PEPC1* was determined as C_4 version, which was not found in Fbid2, which may be a result of uncompleted assembly in this species (Abbreviations: Frob: *F. robusta*, Fflo: F. *floridana*; Fbro: *F. brownii*; Fbid: *F. bidentis*, Fson: *F. sonorensis*; Fram: *F. ramosissima*, Ftri: F. trineriva).

Supplementary Data 5: Determination of functional copies of C_4 genes

Methods

To determine the functional copies of C_4 genes, or C_4 version of C_4 genes, orthologous groups of C_4 genes were first characterized using Orthofinder (v2.3.11) [9] with default parameters. The C_4 versions were determined by combining gene phylogenetic tree and transcript abundances. To construct gene trees, protein sequences were aligned with MUSCLE (v3.8.31) [10] in default parameters, and Raxml (v7.9.3)[11] was applied to construct the gene tree based on the aligned protein sequences using the PROTGAMMAILG model (General Time Reversible amino acid substitution model with assumption that variations in sites follow gamma distribution). Transcript abundances were calculated based on RNA-seq data from a 4-week low CO_2 experiment.

Results

As most C_4 genes belong to gene family with multiple paralogs both across both C_3 and C_4 species, the C_4 version of C_4 genes was determined using the following two criteria: 1) showing higher transcript abundance in C_4 than in C_3 species, and 2) higher transcript abundance among its paralogous group. The C_4 versions of C_4 genes show higher transcript abundance in C_4 species than in other species, and usually are the highest paralog copy in C_4 species (Supplementary data Fig. 6). For these determined C_4 version of C_4 genes, the evolutionary pattern of the C_4 version of C_4 genes in transcript and protein abundances were consistent (Supplementary data Fig. 7).

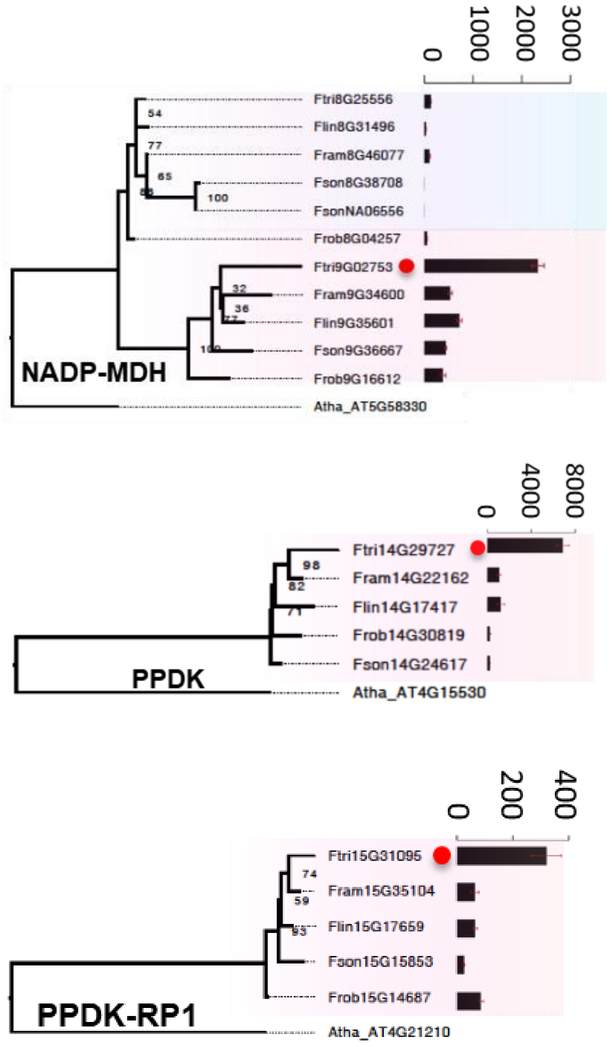
NADP-MDH
Ftri8G25556
Flin8G31496
Fram8G46077
Fson8G38708
FsonNA06556
Frob8G04257
Ftri9G02753
Fram9G34600
Flin9G35601
Fson9G36667
Frob9G16612
Atha_AT5G58330
0
1000
2000
3000
PPDK
Ftri14G29727
Fram14G22162
Flin14G17417
Frob14G30819
Fson14G24617
Atha_AT4G15530
0
4000
8000
PPDK-RP1
Ftri15G31095
Fram15G35104
Flin15G17659
Fson15G15853
Frob15G14687
Atha_AT4G21210
0
200
400

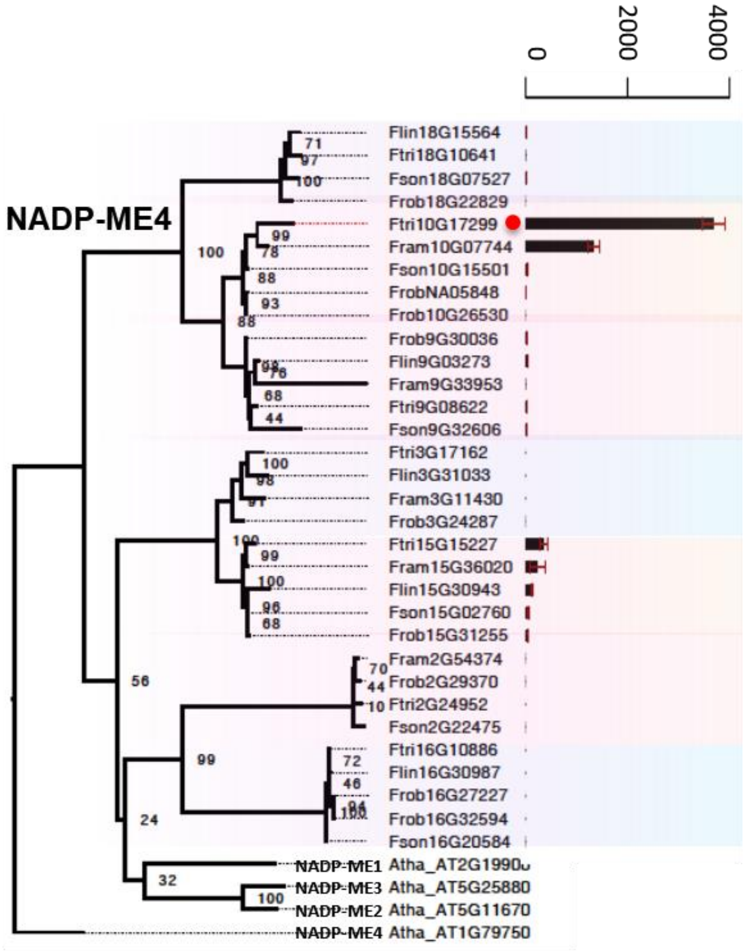
0
2000
4000
NADP-ME4
Flin18G15564
Ftri18G10641
Fson18G07527
Frob18G22829
Ftri10G17299
Fram10G07744
Fson10G15501
FrobNA05848
Frob10G26530
Frob9G30036
Flin9G03273
Fram9G33953
Ftri9G08622
Fson9G32606
Ftri3G17162
Flin3G31033
Fram3G11430
Frob3G24287
Ftri15G15227
Fram15G36020
Flin15G30943
Fson15G02760
Frob15G31255
Fram2G54374
Frob2G29370
Ftri2G24952
Fson2G22475
Ftri16G10886
Flin16G30987
Frob16G27227
Frob16G32594
Fson16G20584
NADP-ME1 Atha_AT2G1990
NADP-ME3 Atha_AT5G25880
NADP-ME2 Atha_AT5G11670
NADP-ME4 Atha_AT1G79750

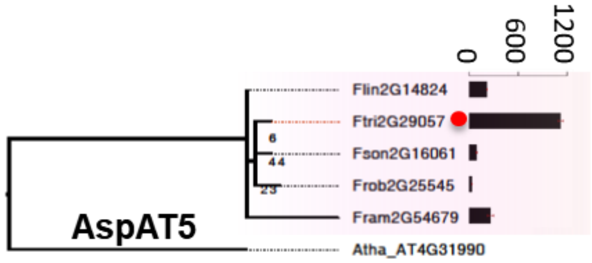
0
600
1200
Flin2G14824
Ftri2G29057
Fson2G16061
Frob2G25545
Fram2G54679
AspAT5
Atha_AT4G31990

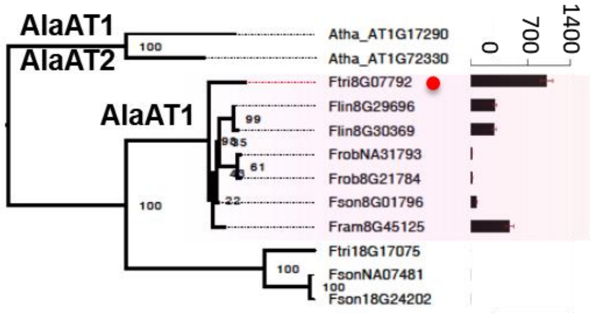
AlaAT1
AlaAT2
Atha_AT1G17290
Atha_AT1G72330
0
700
1400
Ftri8G07792
AlaAT1
Flin8G29696
Flin8G30369
FrobNA31793
Frob8G21784
Fson8G01796
Fram8G45125
Ftri18G17075
FsonNA07481
Fson18G24202

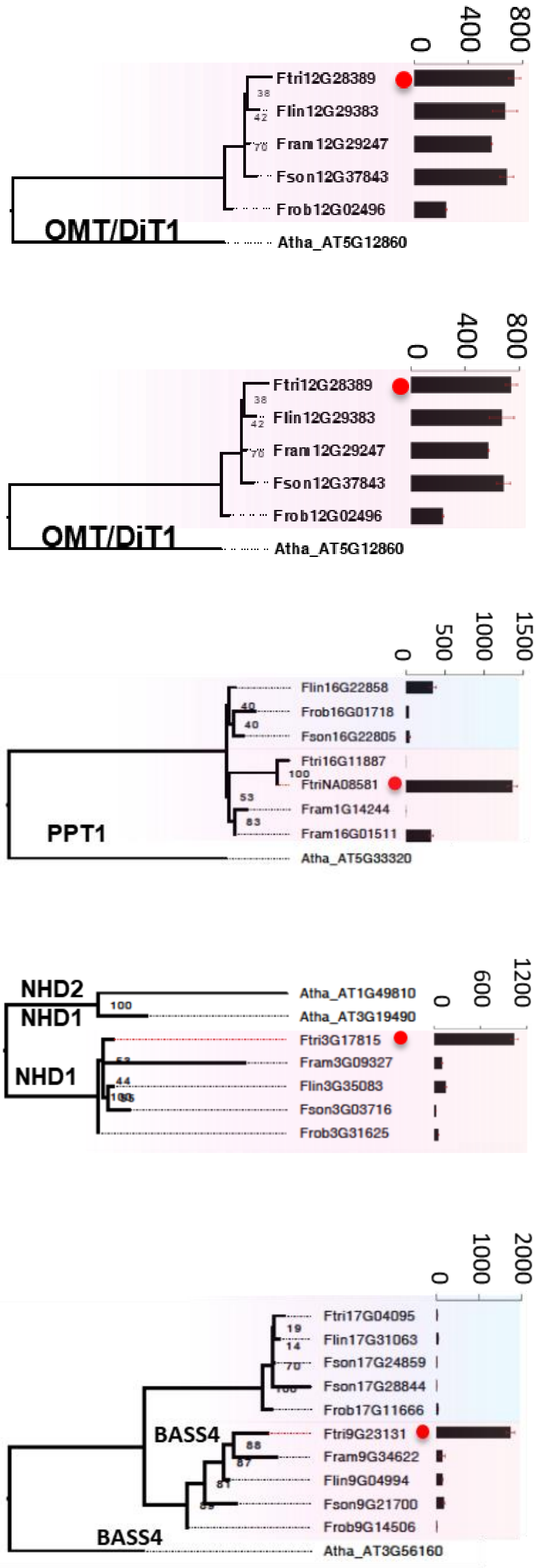
0
400
800
Ftri12G28389
Flin12G29383
Fram12G29247
Fson12G37843
Frob12G02496
OMT/DiT1
Atha_AT5G12860
0
400
800
Ftri12G28389
Flin12G29383
Fram12G29247
Fson12G37843
Frob12G02496
OMT/DiT1
Atha_AT5G12860
0
500
1000
1500
Flin16G22858
Frob16G01718
Fson16G22805
Ftri16G11887
FtriNA08581
Fram1G14244
Fram16G01511
PPT1
Atha_AT5G33320
NHD2
NHD1
NHD1
Atha_AT1G49810
Atha_AT3G19490
Ftri3G17815
Fram3G09327
Flin3G35083
Fson3G03716
Frob3G31625
0
600
1200
0
1000
2000
Ftri17G04095
Flin17G31063
Fson17G24859
Fson17G28844
Frob17G11666
Ftri9G23131
Fram9G34622
Flin9G04994
Fson9G21700
Frob9G14506
BASS4
BASS4
Atha_AT3G56160

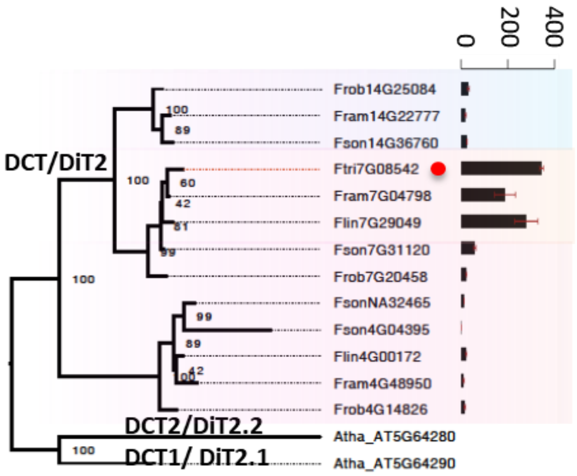

Supplementary data Fig. 6: Determining the C_4 version of C_4 genes combining gene tree and transcript abundances

Gene tree and transcript abundances of each C_4 genes. Genome-wide orthologous groups were predicted using Orthofinder. Gene trees were constructed using protein sequences from Raxml. Bootstraps were calculated from 100 independent trees. Gene expression in transcript per kilobase per million mapped reads (TPM) is shown. The bars show mean ± S.D. (n=3 biological replicates). The predicted functional versions of C_4 genes are indicated with red circles.

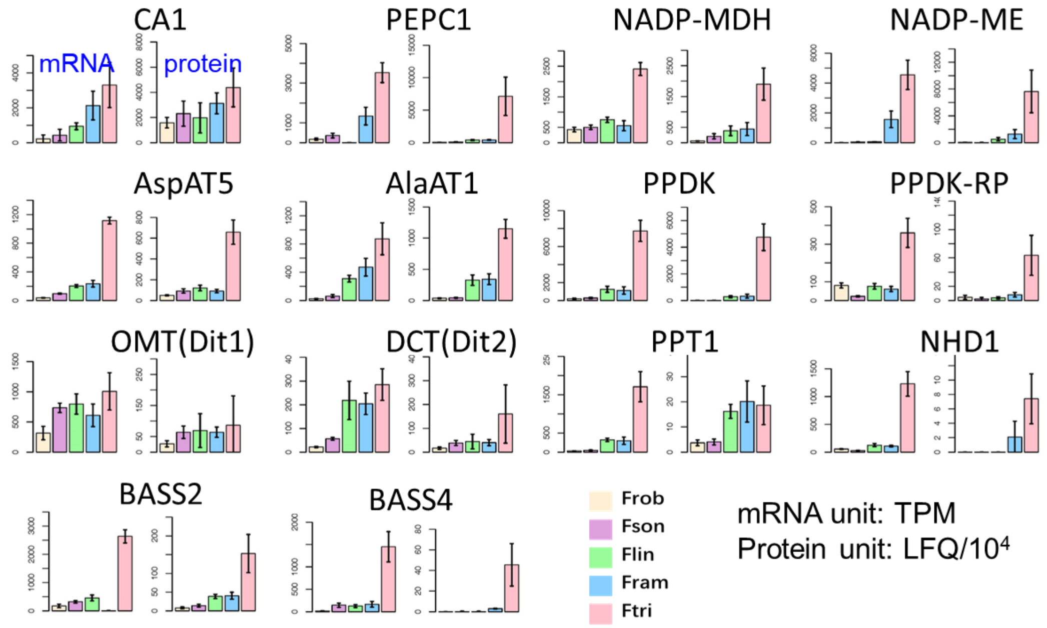

Supplementary data Fig. 7: C_4 genes show a consistent evolutionary profile in transcript and protein abundances across the five *Flaveria* species

Bar plots illustrate the transcript abundances (left) and protein abundances (right) of C_4 enzyme and transporters from the five *Flaveria* species. The Y-axis represents transcript per million mapped reads (TPM) for transcript abundances and label free quantification (LFQ)/10^4 for protein abundance. The bars show mean ± S.D. (n=6 biological replicates, for both transcript abundances and protein abundances) (Abbreviations: TPM: transcript per million mapped reads, LFQ: label free quantification, *CA1*, *carbonic anhydrase 1*; *PEPC1*: *phosphoenolpyruvate carboxylase 1*; *NADP-MDH*: *NADP-dependent malate dehydrogenase*; *NADP-ME4*: *NADP-dependent malic enzyme 4*; *AspAT*: *Aspartate amino acid transport*; *AlaAT*: *alanine aminotransferase*; *PPDK*: *pyruvate orthophosphate dikinase*; *PPDK-RP*: *PPDK regulatory protein*; *OMT*: *oxaloacetate/malate transporter or dicarboxylate transporter 1* (*DiT1*); *DCT*: *dicarboxylate transport 2.1* (or *DiT2.1*); *PPT1*: *phosphate/phosphoenolpyruvate translocator 1*; *NHD1*: *sodium: hydrogen antiporter 1*;*BASS2*: *bile acid sodium symporter 2*; *BASS4*: *bile acid sodium symporter 4*; *PEPC kinase* (*PEPC-k*) was not included as the C_4 version of *PEPC-k* was not detected in Fram)

Supplementary Data 6: C_4 version of *PEPC-k* was absent in Fram plant sequenced in this study

Methods

We found that the C_4 version *PEPC-k* was absent in Fram (Fig. 2b). We checked whether this was due to an error in genome assembly. We investigated the presence and absence of adjacent genes to *PEPC-k* in all five *Flaveria* species. Specifically, we analyzed six genes upstream of *PEPC-k* and six genes downstream of *PEPC-k* according to the chromosome location of Frob (13 genes in total). We then checked whether the orthologous genes were present in the other four *Flaveria* species.

Results

We found that the counterparts of the 13 genes were also present in the C_4 species Ftri, while, one of the orthologous genes was absent in Fson. Notably, 7 genes in tandem were absent in the Flin genome, and 6 genes in tandem were absent in the Fram genome, including *PEPC-k* (Supplementary data Fig. 8a). This suggests that the genome segments containing *PEPC-k* were dynamic during the evolution in the genus of *Flaveria*. We then checked whether the deletion in Fram was due to an assembly gap. We found that the sequence is not “N”, suggesting that this may be not a gap (Supplementary data Fig. 8b and c). Therefore, our results suggested that the Fram plant sequenced here had a deletion of the C_4 version of *PEPC-k*.

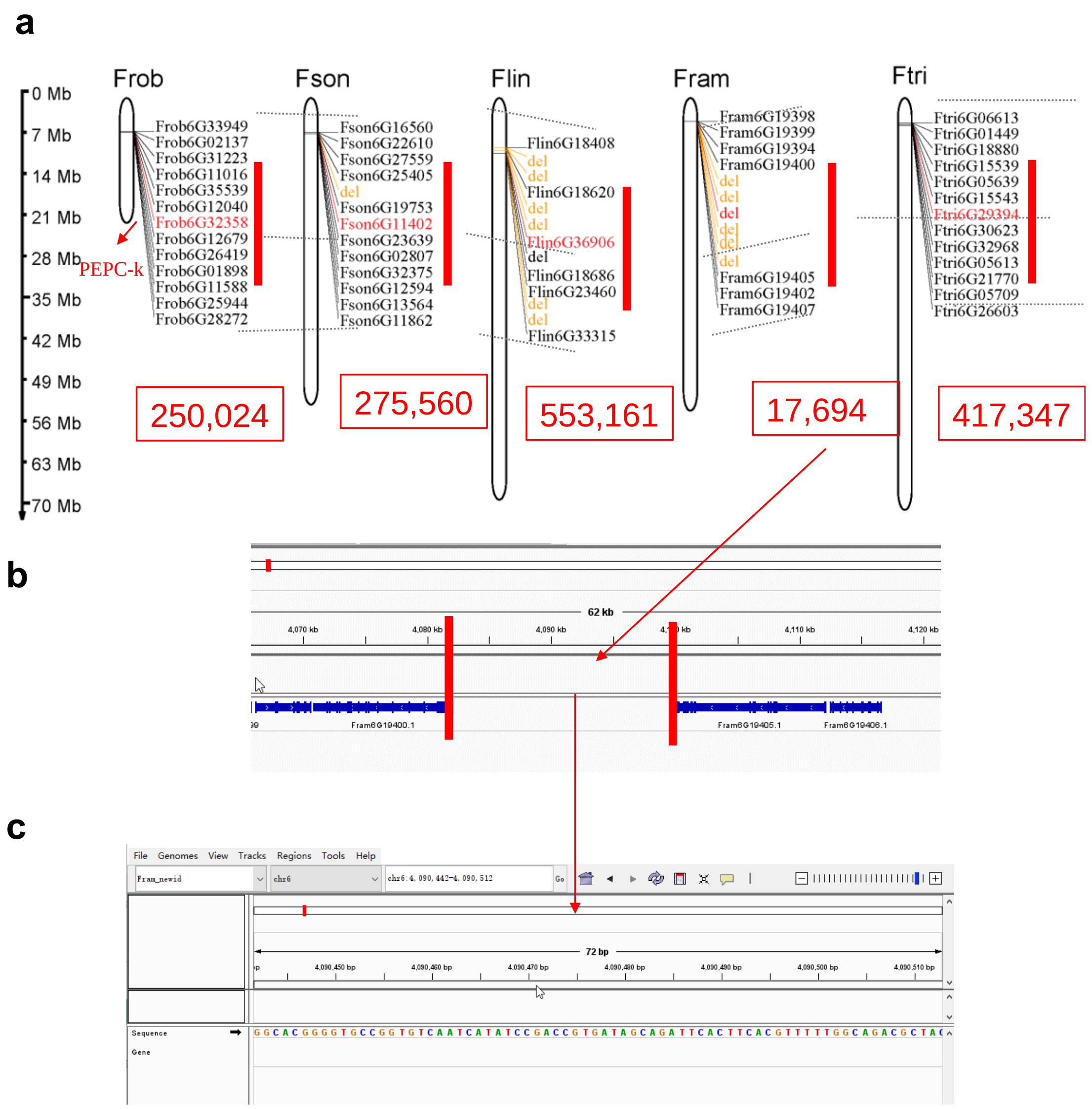

Supplementary data Fig. 8: Syntenic chromosome region containing *PEPC-k* across the five *Flaveria* species

(a) The syntenic chromosome region containing *PEPC-k*. Six genes from upstream and downstream of *PEPC-k* according to Ftri are also shown. *PEPC-k* is labeled in red font, a deletion is represented by “del” in orange. The number in the red frame indicates the length of the chromosome segment. (b) IGV indicates the chromosome region of the deletion of the six genes (between Fram6G19400 and Fram6G19405, including *PEPC-k*) in Fram. (c) The nucleotide sequences between Fram6G19400 and Fram6G19405. Note that the sequence is not “N”, suggesting that the deletion is not due to an assembly gap.

Supplementary Data 7: Verification of three copies of PEPC1 in the C_4 species Ftri

Methods

To verity the presence of three copies of *PEPC1* in Ftri, genomic DNA was isolated from young leaves of one-month old plants. DNA isolation was performed as described previously [12]. Forward primers were designed to be complementary to the non-conserved region of the 5’ UTR of the three *PEPC1* copies respectively, and the reverse primer was designed to be complementary the conserved region of the coding sequence. The forward primers were: TCGTATTTAATCTTTCGCAGGTTTAAAAATATT for Ftri3G07887 (*PEPC1.1*), GCATTAGGTTTGAGATAGCCTG for Ftri3G16655 (*PEPC1.2*), and ACGGCAACGTGCGCATA for Ftri3G30452 (*PEPC1.3*). The reverse primer was identical for three loci: TATCCAAAAGCAAAGCATCATACTC.

Results

The three copies of *PEPC1* in the C_4 species Ftri were verified by PCR. (Supplementary data Fig. 9).

a

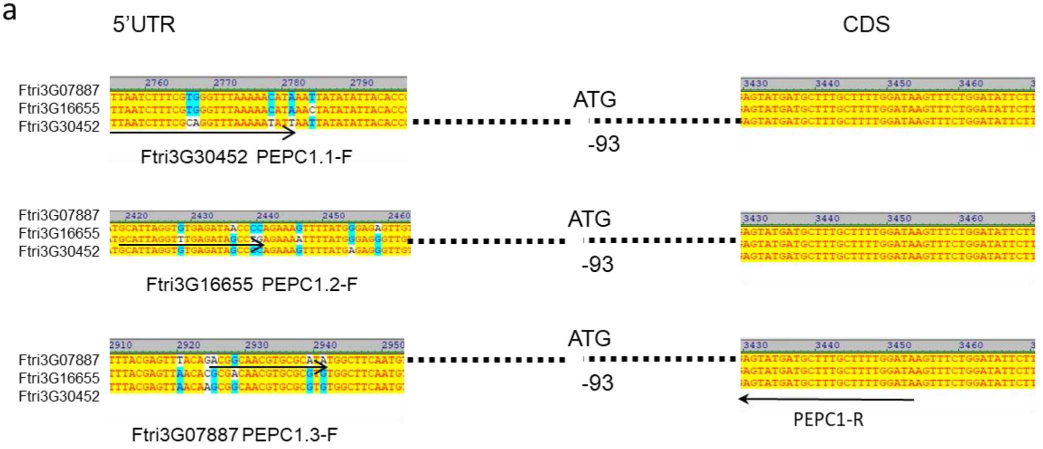

b

Paralogs	PEPC1.1-F	PEPC1-R
PEPC 1.1	TCGTATTTAATCTTTCGCAGGTTTAAAAATATT	TATCCAAAAGCAAAGCATCATACTC
PEPC 1.2	GCATTAGGTTTGAGATAGCCTG	
PEPC 1.3	ACGGCAACGTGCGCATA	

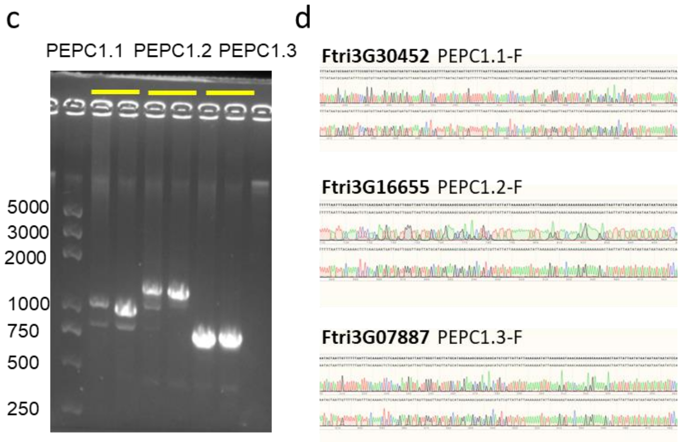

Supplementary data Fig. 9: Verification of three *PEPC1* paralogs in Ftri using PCR

(a) Schematic of the designed forward and reverse primers of there *PEPC1*s in Ftri. The forward primers are complementary to the 5'UTR where the sequences are different between the three *PEPC1*s. The reverse primer is complementary to the conserved protein coding region. Primer sequences for each *PEPC1* paralog are shown in (b), gel results and Sanger sequencing are shown in (c) and (d).

Supplementary Data 8: Investigation of tandem duplications of C_4 genes in other C_4 species

Methods

To investigate whether C_4 genes show tandem duplication in other C_4 species, we expanded the evolution of C_4 genes to more species. In total, two dicotyledonous C_4 species, *i.e.*, Ftri and *Gynandropsis gynandra* (Ggyn), and six monocotyledonous C_4 species, *i.e.*, *Zea mays* (V5), *Setaris viridis* (V2.1), *Panicum Hallii* (V2.1), *Sorghum biocolor* (v3.0.1), and *Miscanthus lutarioriparius* (V1) were included in the comparisons. Additionaly, six dicotyledonous C_3 species and two monocotyledonous C_3 species, as well as one algal species (*Chlamydomonas reinhardtii*) were included. The protein sequences of those species were downloaded from public databases as mentioned in the Methods of the manuscript. Orthologous genes were predicted using Orthofinder (v2.3.11) [9] with default parameters, orthologous protein sequences were aligned by MUSCLE (v3.8.31) [10] with default parameters, and gene trees were constructed using Raxml (v7.9.3) [11] based on alignments of protein sequences with the PROTGAMMAILG model (General Time Reversible amino acid substitution model with assumption that variations in sites follow gamma distribution).

To investigate whether the same orthologous genes were co-opted in dicotyledonous and monocotyledonous C_4 species, we quantified transcript abundances of genes for all C_4 species (Supplementary data Fig. 10) and several C_3 species, including *Oryza sativa*, *Arabidopsis thaliana*, *Tarenaya hassleriana,* and the algae species *Chlamydomonas reinhardtii*. The RNA-seq data used here are mentioned in Supplementary data 14.

Results

Our results indicated that tandem duplication of C_4 version of *CA*, *PEPC*, and *PEPC-k* were not universal but rather, species-specific (Supplementary data Fig. 10). Notably, we found that the same orthologous genes were recruited for C_4 photosynthesis across different C_4 species. Remarkably, the counterparts of recruited

C_4 genes already showed high transcript abundances in C_3 species.

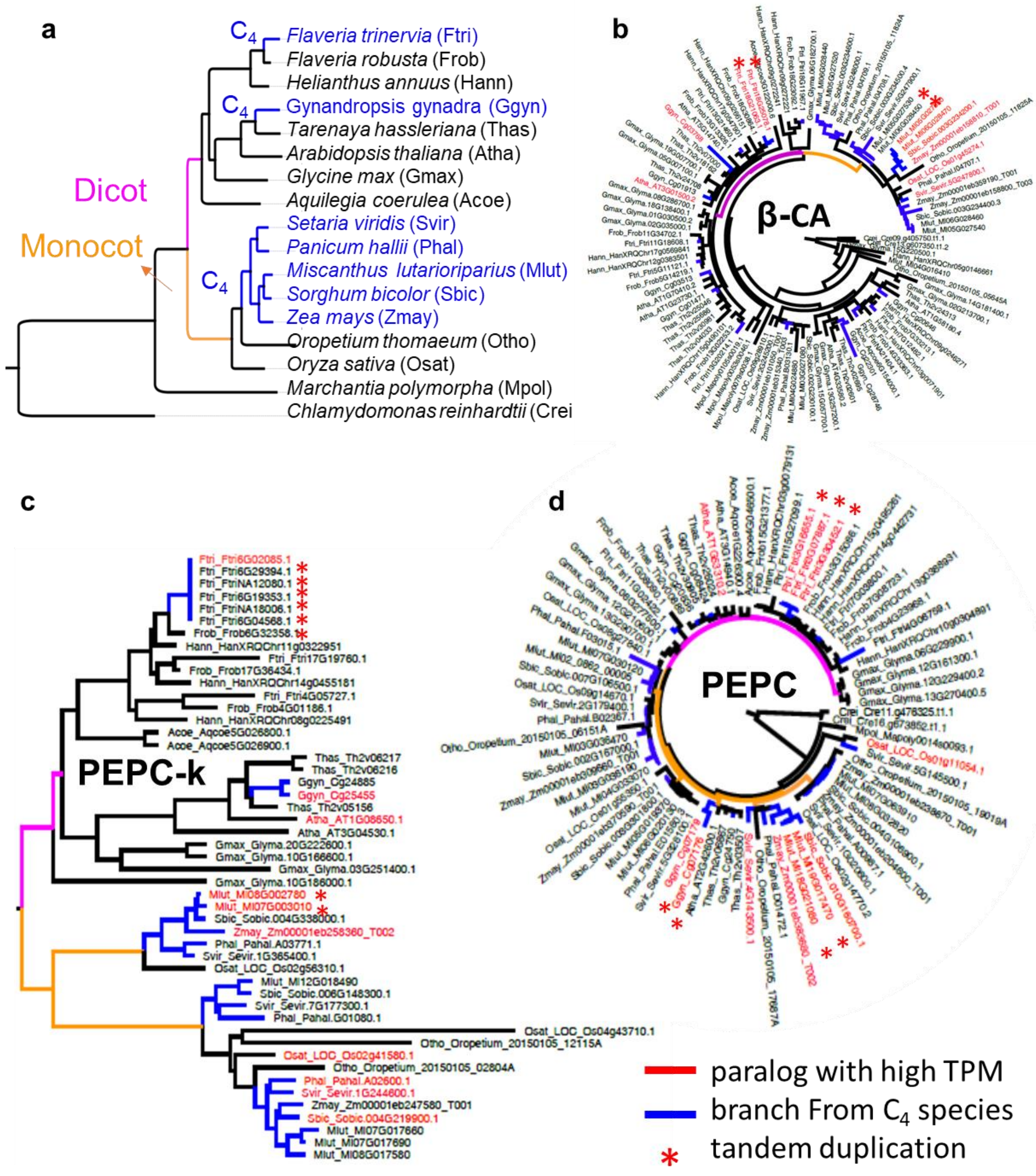

Supplementary data Fig. 10: Tandem duplications of C_4 version of *CA*, *PEPC* and *PEPC-k* are not universal but species-specific

(a) Phylogenetic tree of species to investigate tandem duplication of C_4 genes. Phylogenetic relationships were inferred from the Phytozome website (https://phytozome-next.jgi.doe.gov). C_4 species are labeled in blue. Abbreviations of each species using in the following gene tress are shown in parentheses after each species. The gene trees of *CA* (b), *PEPC-k* (c), and *PEPC* (d) are shown, where the main branch of monocotyledonous plants are shown in orange with dicotyledonous plants in purple. Branches labeled in blue are C_4 species, the gene with the highest TPM is labeled in red font. The format of the gene on the gene tree is as follows: "abbreviation of species name" pluses gene id jointed with "_".

Supplementary Data 9: Analysis of transposable elements and their effects on duplicated Ftri*PEPC1*

Methods

To predict transposable elements (TEs), entire genome sequences of the five *Flaveria* species were searched for repetitive sequences individually. A *de novo* repeat sequence library was obtained using RepeatModeler (RepeatModeler-Open-1.0.5) with the following parameters: RepeatModeler -database database_name -engine ncbi -pa [int]. We then used RepatMasker (RepeatMasker-Open-4.1.0) to search for similar TEs against the de novo library with the following parameters: RepeatMasker genome.fa -lib de_novo_library -nolow -no_is -q -engine rmblast -pa [int] –norna. Intact long terminal repeat retrotransposons (LTR-RTs) were identified using LTR_FINDER (v1.07) [13] and LTRharvest (v1.5.10) [14] with default parameters. LTR_Retriever (v2.9.0) [15] was then used to merge the above results with the following parameters: LTR_retriever -genome genome.fa -inharvest species.harvest.scn -infinder species.finder.scn –nonTGCA species.harvest.nonTGCA.scn. The insertion time of intact LTR-RT was obtained from LTR-Retriever analysis.

Results

Transposable elements (TEs) showed the highest abundance in the C_4 species, where they accounted for 82% of the total genome, followed by C_3-C_4 species (from 65.6% to 71.8%), while the percentage in the C_3 species was 47.1% (Supplementary data table 5). In all five species, long terminal repeat retrotransposons (LTR-RTs) comprised the majority of the TEs, accounting for an average of 76% of the total TEs (from 42% to 91%) (Supplementary data table 6 and Supplementary data table 7). Flin and Fram had more evolutionary recent LTR-RTs than the other species (Supplementary data Fig. 11). Compared to C_3 and intermediate species, C_4 species Ftri had a higher proportion of TEs at the whole genome scale.

We found that abundant retrotransposons were on the chromosomal regions between tandem duplicated *FtriCA1*, and the region between tandem duplicated *FtriPEPC1* and the region between tandem duplicated *FtriPEPC-K1* (Supplementary data Fig. 12). In comparison, few retrotransposons were observed in the chromosome near the region containing *FtriPPDK*, which was a single copy gene.

Reports have showed that gene duplications could be mediated by retrotransposons through retroposition[161718192021]. To test whether the observed C_4 gene duplications were related to retrotransposons in Ftri, we took a close inspection of the evolution and sequences of Ftri*PEPC1*s. Among the three Ftri*PEPC1s*, Ftri*PEPC1.1* was predicted to be the ancestral copy, as the mesophyll expression module 1 (MEM1) on the promoter of Ftri*PEPC1.1* was conserved with that of *PEPC1* from other four *Flaveria* species, i.e., Frob, Fson, Fram and Flin, whereas, the MEM1 of Ftri*PEPC1.2* and Ftri*PEPC1.3* showed a deletion of 109 bps (Supplementary data Fig. 13). Except for coding region, sequences from ~2500bp upstream and ~2000 bp downstream of the coding sequences of Ftri*PEPC1s* were also conserved among the three Ftri*PEPC1*s (Supplementary data Fig. 14b). Through close inspection of the sequences near the conserved region, we observed a 9-bp inverted repeat sequences, *i.e.*, 5’-AAAATAAAG-3’. Besides, a 4-bp motif, *i.e.*, 5’-TTTT-3’, immediately flanked the invert repeats (Supplementary data Fig. 14c).

To further test whether retrotransposons were related to duplications of DNA segments, we examined chromosomal regions containing duplicated copies of *CA1*, *PEPC1* and *PEPC-k1* in Ftri. We found that these chromosomal regions contain many duplicated sequences (Supplementary data Fig. 15). Taken together, our results suggest that the gene duplications of C_4 genes might be mediated by retrotransposons.

Supplementary data table 5: Compositions and proportions of transposable elements across the five *Flaveria* species

	Frob (% of genome)	Fson (% of genome)	Flin (% of genome)	Fram (% of genome)	Ftri (% of genome)
**Total Repeat Fractions**	47.09	70.26	71.81	65.56	82.06
**ClassⅠ:Retrotransposon**	20.41	57.27	59.52	56.29	75.97
**LTR Retrotransposon**					
Ty1/Copia	12.69	34.1	27.32	38.51	56.6
Ty3/Gypsy	5.97	22.21	29.44	16.81	18.48
Other	1.18	0.72	0.66	0.74	0.32
**NON-LTR Retrotransposon**					
SINE	0.03	0.02	0.01	0.01	0
LINE	0.54	0.22	2.09	0.22	0.57
**Class Ⅱ :DNA transposon**	6.23	4.73	5.1	4.32	1.84
CACTA	0.82	0.71	1	0.82	0.37
Mutator	0.45	0.61	1.48	0.39	0.23
PIF-Harbinger	0.27	0.31	0.27	0.11	0.1
RC/Helitron	0.4	0.64	0.25	0.2	0.11
hAT	2.21	1.62	0.77	0.88	0.6
Other	2.08	0.84	1.33	1.92	0.43
**Tandem Repeat**	0.38	0.86	1.86	0.45	0.31
**Unknown**	20.07	7.4	5.33	4.5	3.94

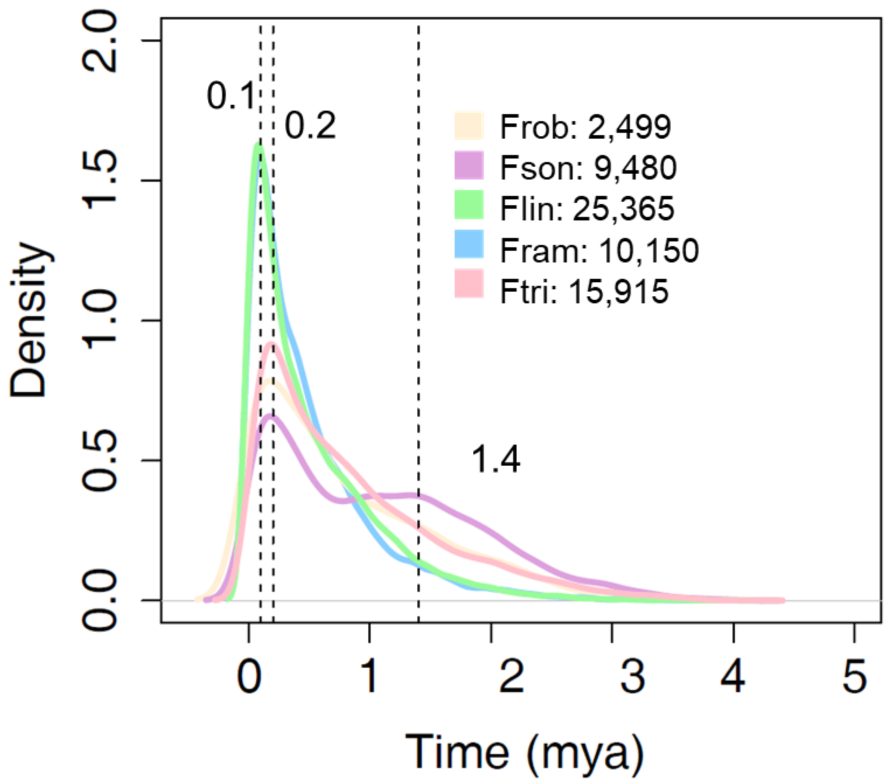

Supplementary data Fig. 11:The burst time of LTR-RTs estimated based on intact LTR-RTs

The distribution of burst time of the contact LTR-RTs of the five *Flaveria* species are shown.

The numbers of contact LTR-RTs for each species are indicated. (Abbreviations of species: Frob, *F. robusta*, Fson, *F. sonorensis*, Flin: *F. linearis*, Fram: *F. ramosissma*, Ftri: *F. trinervia*.)

Supplementary data table 6: The length of different types of transposon elements

Species	Total TE length (Mbp)	Total LTR-RT length (Mbp)	Total intact LTR-RT length (Mbp)	% Intact LTR-TR to TE	% Intact LTR-RT to LTR-RT
**Frob** (C_3)	259.73	109.42	14.68	5.65%	13.42%
**Fson** (C_3-C_4)	872.90	708.48	80.89	9.27%	11.42%
**Flin** (C_3-C_4)	1188.64	950.59	236.96	19.94%	24.93%
**Fram** (C_3-C_4)	830.21	709.81	101.38	12.21%	14.28%
**Ftri** (C_4)	1480.09	1357.84	168.89	11.41%	12.44%

Note: LTR-RT: long terminal repeat retrotransposons, TE: transposon elements

Supplementary data table 7: The different types of TEs

Species	TE	LTR-RT	Intact LTR-RT	% Intact LTR-RT to TE	% Intact LTR-RT to LTR-RT
**Frob** (C_3)	571,290	129,171	2,499	0.44%	1.93%
**Fson** (C_3-C_4)	985,595	532,042	9,480	0.96%	1.78%
**Flin** (C_3-C_4)	1,298,249	767,238	25,365	1.95%	3.31%
**Fram** (C_3-C_4)	747,356	371,205	10,150	1.36%	2.73%
**Ftri** (C_4)	1,045,859	687,601	15,915	1.52%	2.31%

Note: LTR-RT: long terminal repeat retrotransposons, TE: transposon elements

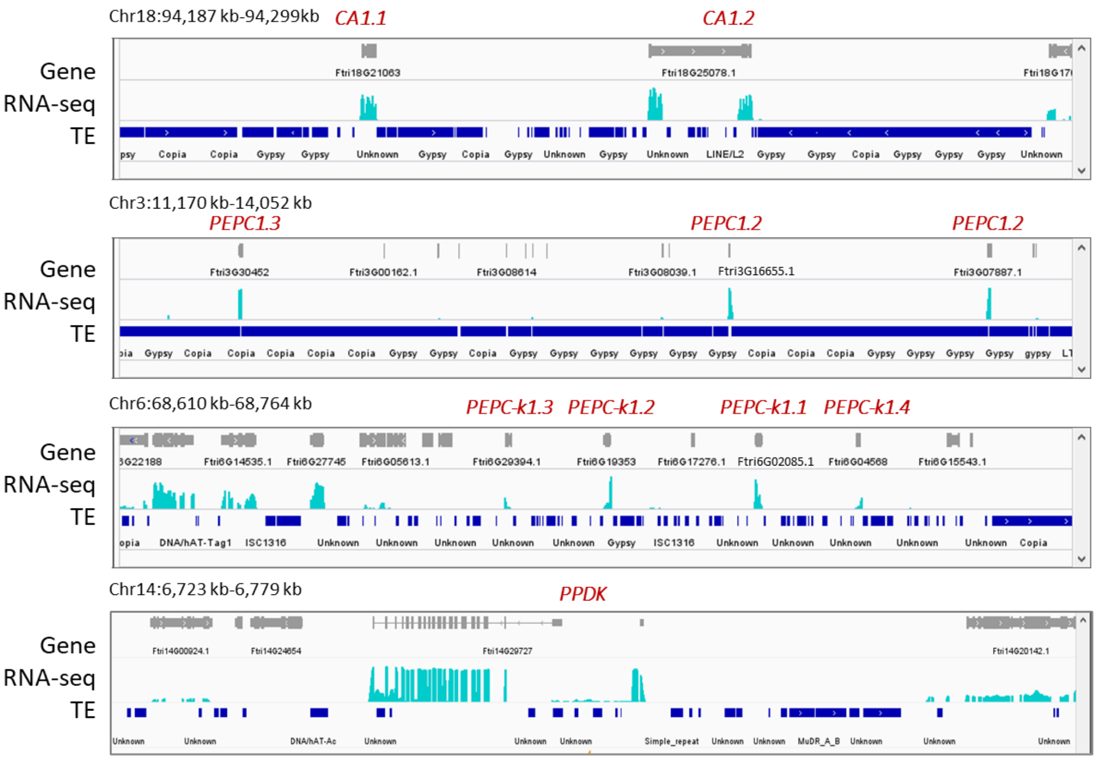

Supplementary data Fig. 12: Retrotransposons may be related to tandem duplication of *CA1*, *PEPC1* and *PEPC-k1* in Ftri.

Integrative genomics viewer (IGV) indicating the genes, transcript abundances and predicted TEs on the chromosomal regions between tandem-duplicated *CA1*, *PEPC1* and *PEPC-k1*. Transcript abundances are shown in transcript per million mapped reads (TPM). The chromosome region containing *PPDK* is also illustrated (bottom panel). (Abbreviations: *CA1*, *carbonic anhydrase 1*; *PEPC1*, *phosphoenolpyruvate carboxylase 1*; *PEPC-K*, *PEPC kinase*; *PPDK*, *pyruvate/orthophosphate dikinase*.)

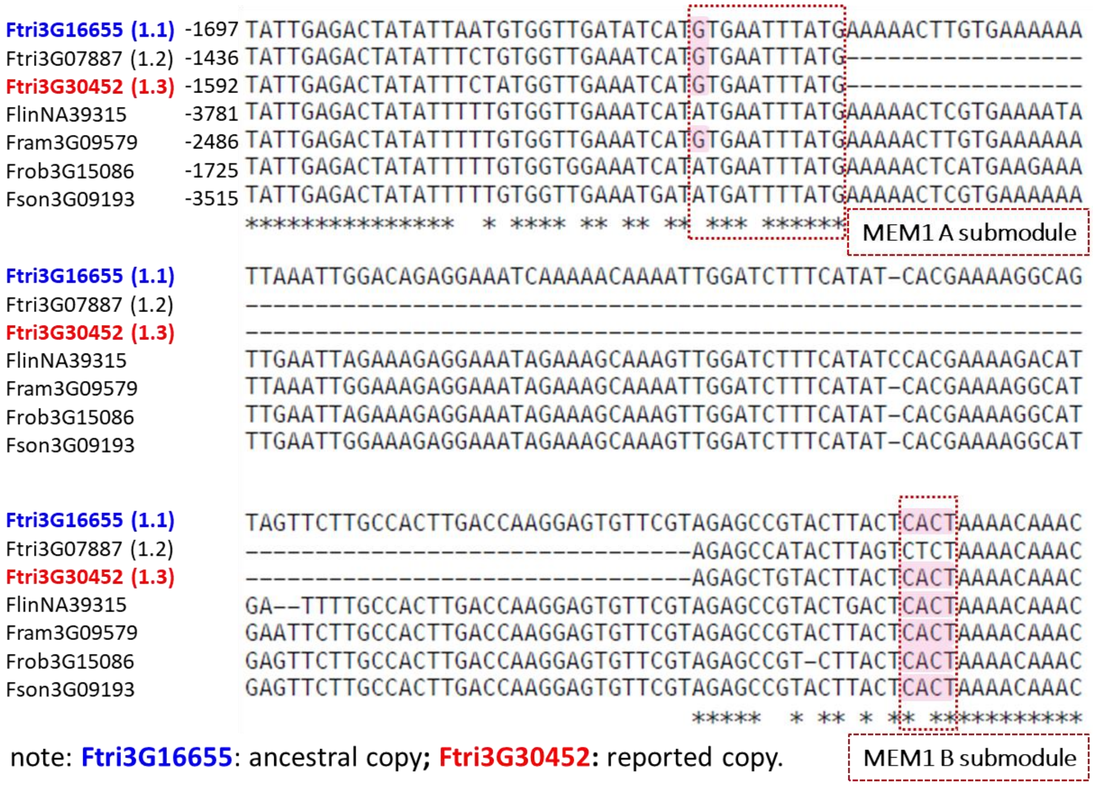

Supplementary data Fig. 13: The Mesophyll expression module 1 in the three Ftri*PEPC1*s

The mesophyll expression module 1 (MEM1) sequences of the *PEPC1* promoters from five *Flaveria* species. The A and B submodules are highlighted in red boxes. Asterisks show identical nucleotides across the two modules. Pink zones indicate single nucleotide difference in the A submodule, and the required tetranucleotide CACT in the B submodule. In Ftri, *Ftri3G1665* (*PEPC1.1*) is the ancestral copy and the other two are latterly duplicated. The duplicated *PEPC1*s show a segment of deletion between A submodule and B submodule. (Abbreviations: MEM: mesophyll expression module, which is required for the mesophyll highly expression of *PEPC* in *Flaveria* C_4 species as reported in a previous study [22])

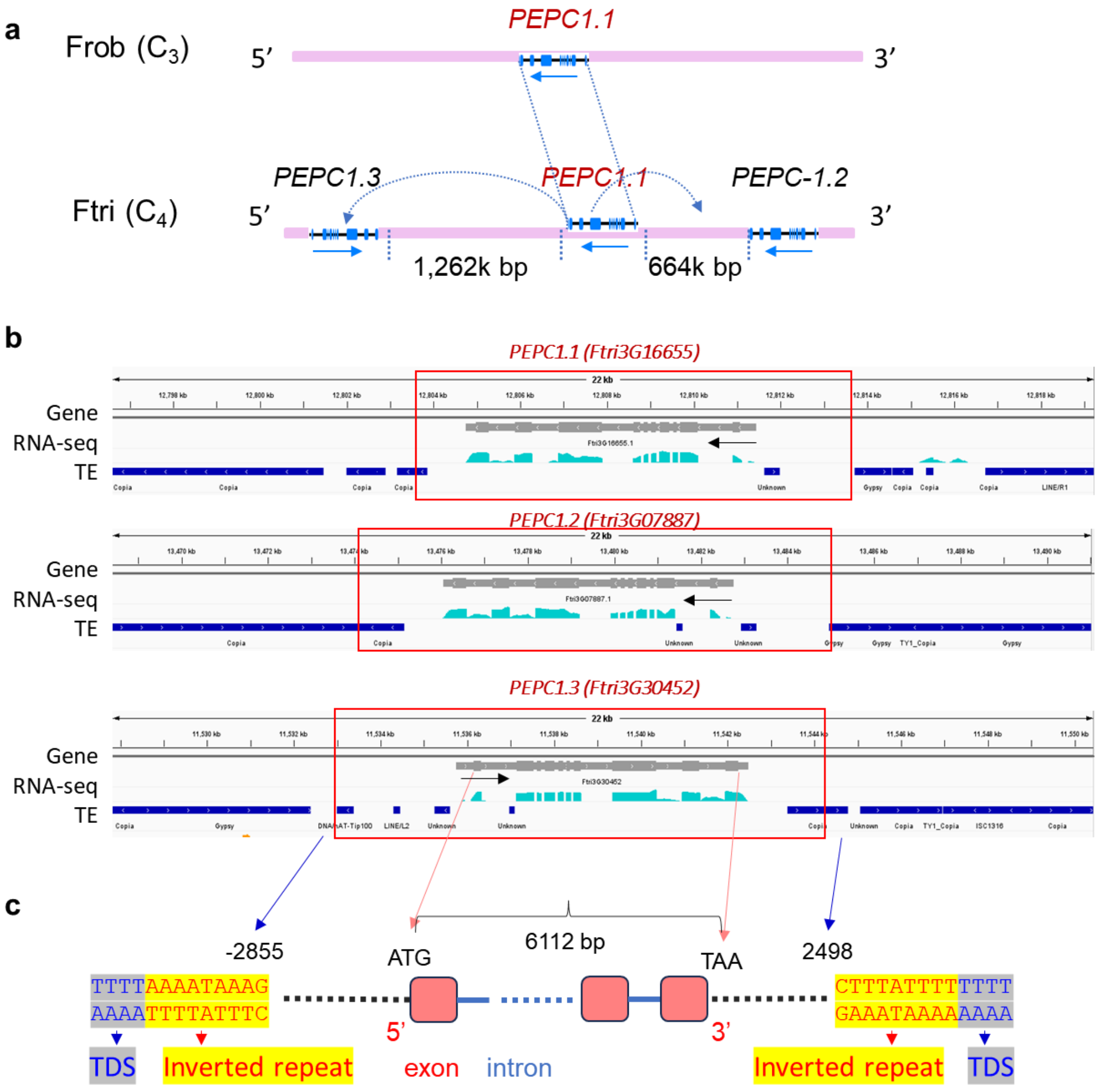

Supplementary data Fig. 14: Tandem duplicated Ftri*PEPC1*s may be mediated by retrotransposons

(a) Schematic illustration of the transposition of Ftri*PEPC1*. (b) Integrative genomics viewer (IGV) indicating genes, transcript abundances and predicted TEs on the chromosomal regions near three Ftri*PEPC1*s. The conserved regions of the three Ftri*PEPC1s* were shown in red frame. (c) Inverted repeats (red font in yellow background) were observed adjacent to the conserved region of Ftri*PEPC1.3*. A 4-bp motif (blue font in grey background) flanks the inverted repeats, resembling a target site duplication (TSD) in transposition event mediated by retrotransposons.

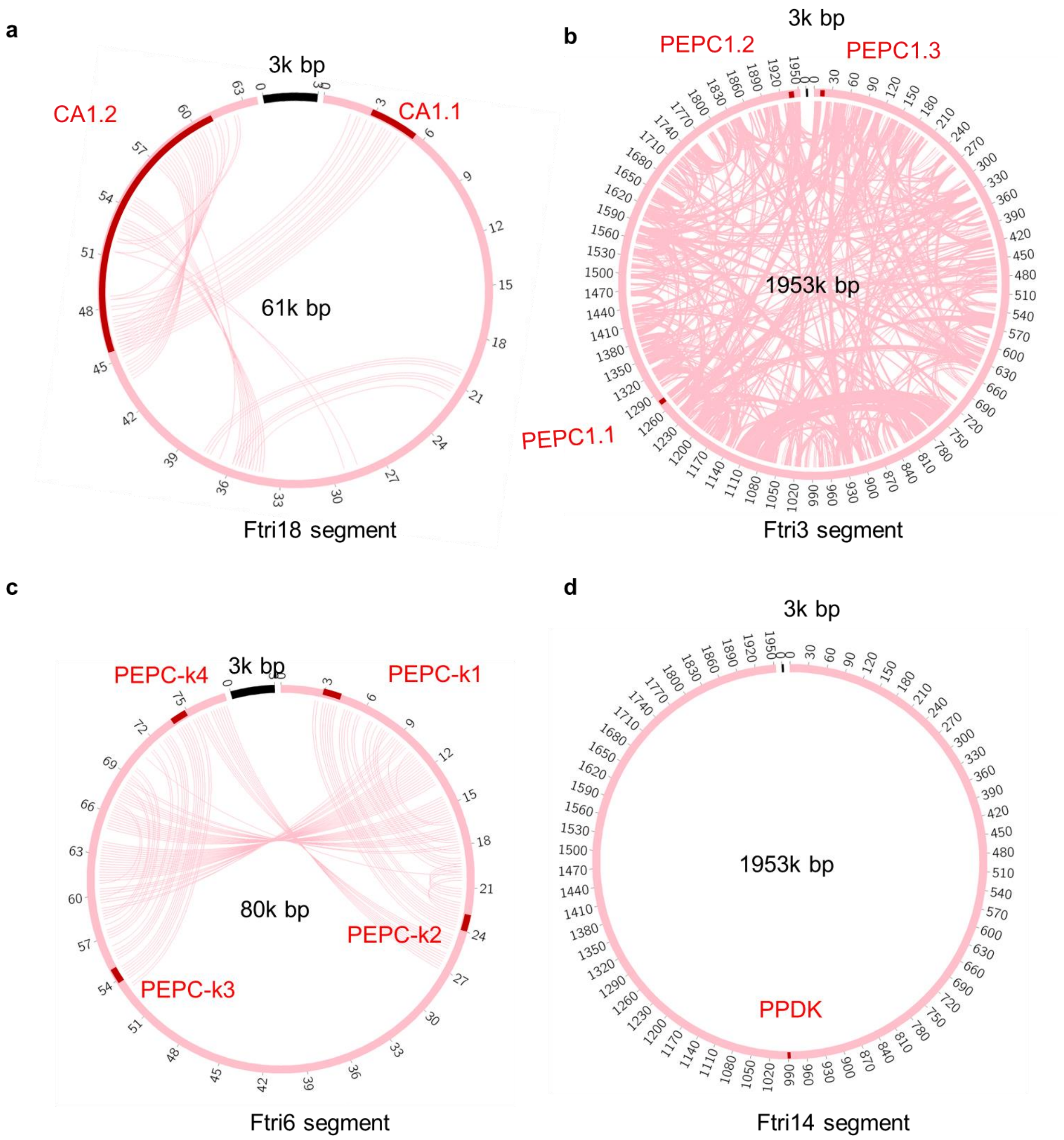

Supplementary data Fig. 15: Chromosome segments containing tandem duplicated C_4 gene copies in Ftri

The chromosome segment containing tandem duplicated *CA1*(a), *PEPC1* (b) and *PEPC-k1* (c) in Ftri. The lengths of shown chromosome segments were as indicated in the circles. (d) The chromosome segment containing *PPDK* in Ftri, which showed a length of 1953k bp of chromosome 14. We used a length of 1953kbp to compare against chromosomal segment on chromosome 13 containing three *PEPC1*s in (b). The 3kbp region in black is used as a scale of the chromosome segment size. Lines inside the circles represent duplicated segments with 200bp. Note that abundant duplicated sequences were observed in the chromosome regions containing duplicated paralogs of *CA1*, *PEPC1* and *PEPC-k1*, but not in the chromosome region containing *PPDK*, which is a singleton gene.

Supplementary Data 10: Comparison of transcript abundances based on RNA-seq data

Methods

RNA-seq data from Frob, Fson, Fram and Ftri are from our previous study [23]. Additionaly, 18 RNA-seq datasets of Flin were generated in this study, including six RNA-seq datasets from a 2-week low CO_2 experiment (three from normal CO_2 condition, *i.e.*, 380 ppm, and three from low CO_2 condition *i.e.*, 100 ppm), six RNA-seq samples from a 4-week low CO_2 experiment (three from normal CO_2 condition and three from low CO_2 condition), and six RNA-seq datasets from high light experiment (three from normal light condition, *i.e.*,500 μmol m^{-2} s^{-1}, and three from high light condition, *i.e.*, 1400 μmol m^{-2} s^{-1}). The low CO_2 experiments on Flin were conducted with those of the other four species. Plants were grown in a growth chamber with a photosynthetic photon flux density (PPFD) controlled to be 200 μmol m^{-2} s^{-1}, and a temperature of 22 ±2 °C, 70% relative humidity (RH), and a photoperiod of 16 hours light/8 hours dark. The CO_2 concentration used for the low CO_2 treatment was 100 ppm and the control CO_2 concentration was 380 ppm. The high light experiment on Flin was conducted together with those of Frob, Fram and Ftri. Plants were grown in a phytotron with a PPFD of 1400 μmol m^{-2} s^{-1} for high light condition and 500 μmol $m^{-2}s^{-1}$ for normal light condition. The high light condition was achieved by supplementing light with a lab-made light emitting diode (LED) light source. Genes were clustered into 10 patterns using Kmeans based on their transcript abundance across the five *Flaveria* species.

Results

To compare transcript profiling of the five *Flaveria* species, three RNA-seq datasets from the normal CO_2 condition of the 2-week low CO_2 experiment and three from the normal CO_2 condition of the 4-week low CO_2 experiment were used for all five *Flaveria* species. Transcript abundances were calculated as transcript per million mapped reads (TPM). In total, 27,684 genes were retained with the maximum transcript abundance no less than 1 TPM in the five *Flaveria* species. We found that

six replicates of a certain species had high Pearson correlations (Supplementary data Fig. 16). The 27,684 genes were clustered into 10 patterns according to their evolutionary profiles (Supplementary data Fig. 17). C_4 genes were mainly clustered in one pattern (P3), in which C_4 species showed the highest transcript abundances, and photorespiratory genes were mainly clustered in one pattern (P8), where Flin showed the highest transcript abundance.

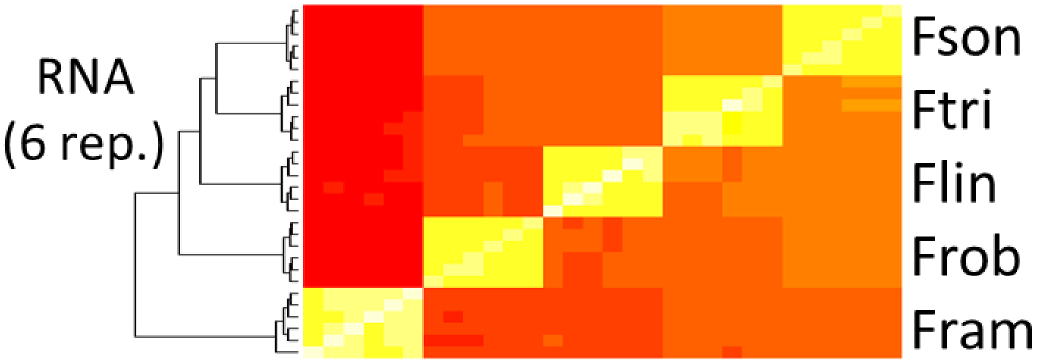

Supplementary data Fig. 16: Pearson correlations of RNA-seq datasets for five *Flaveria* species in transcript abundances

(a) Heatmap showing the Pearson correlations of RNA-seq samples based on transcript abundances of all detected genes. The transcript abundances were calculated as transcript per million mapped reads (TPM). Six RNA-seq samples were used to compare the gene expression profiles along evolutionary history, including three RNA-seq samples from normal CO_2 condition of 2-week low CO_2 experiment, and three RNA-seq samples from normal CO_2 condition of 4-week low CO_2 experiment.

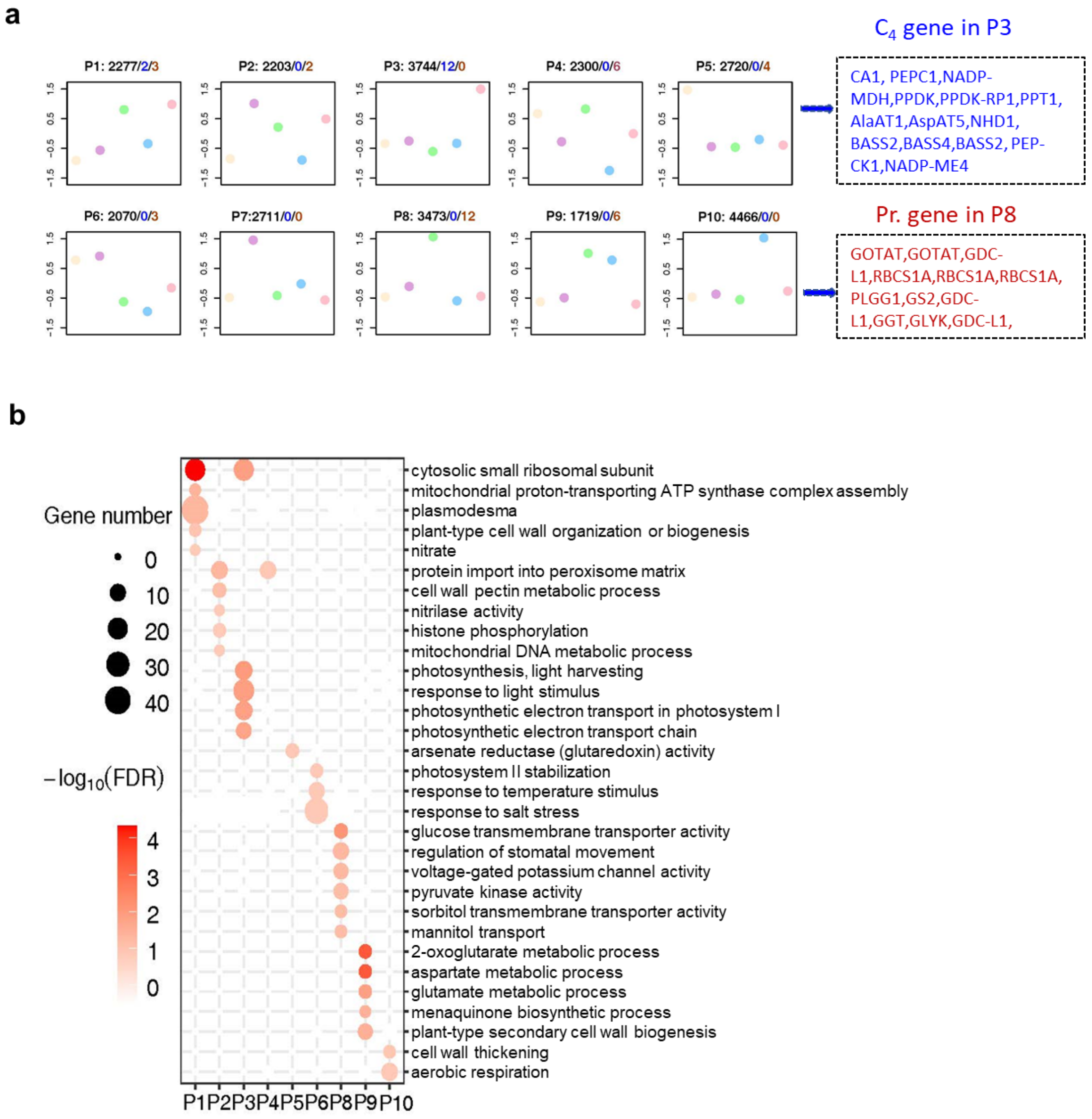

Supplementary data Fig. 17: Ten evolutionary patterns of the detected genes based on transcript abundances

(a) Genes were clustered into ten patterns (P for short, from P1 to P10) according to their expression profiles in the five *Flaveria* species. Genes were clustered using Kmeans based on mean transcript abundances from six RNA-seq samples for each species (see methods above). The numbers on the top of each pattern represent: black for all genes, blue for C_4 genes and red for photorespiratory genes. C_4 genes in pattern 3 (P3) and photorespiratory genes in pattern 8 (P8) are listed in dashed frame. (b) The enriched gene ontology (GO) terms of genes from each pattern. Pattern 7 (P7) showed no enriched gene ontology. Enriched GO terms were calculated using a Fisher's exact test with a *P* value threshold of 0.01 adjusted using Benjamini & Hochberg method.

Supplementary Data 11: Comparison of protein abundance based on proteomics

Methods

Tryptic digestion

A FASP (Filter-Aided Sample Prep) digestion was adapted for the following procedures in Microcon PL-10 filters. After buffer displacement three times with 8 M Urea in 25 mM ABC (ammonium hydroxide), proteins were reduced using 10 mM DTT at 37 °C for 30 minutes, followed by alkylation with 30 mM iodoacetamide at 25 °C for 45 minutes in the dark, and digestion with trypsin (enzyme/protein at a 1:50 ratio) at 37 °C for 12 h after washed with 20% ACN and buffer displaced three times with digestion buffer (30 mM ABC). After digestion, the solution was filtered, and the filter was washed twice with 15% CAN. All filtrates were pooled and vacuum-dried.

High pH HPLC Fractionation

To generate a data dependent acquisition (DDA) library, peptides were prefractionated using a Dionex UltiMate 3000 HPLC system (Thermo Fisher Scientific, USA) with a C18 column (3 μm, 2× 150 mm, Phenomenex, USA). HPLC solvent A was 10 mM NH_4HCO_3, solvent B was 10 mM NH_4HCO_3 in 80% ACN. Peptides from each sample were mixed (a total of 200 μg), dried, and dissolved with 10mM NH_4HCO_3. The mixture was separated by a linear gradient with a flow rate of 200nL/min. The gradient was set as follows: 5-40% in 25 min, and 40-100% in 5 min. Finally, 30 fractions were mixed into 15 samples, and vacuum-dried completely.

LC-MS/MS Analysis

LC-MS/MS analysis was performed using an EASY-nLC 1200 system (Thermo Fisher Scientific, USA) coupled to an Orbitrap Fusion Lumos mass spectrometer (Thermo Fisher Scientific, USA). Samples were resuspended in 1% FA, and iRT peptides (Biognosys, Switzerland) were added prior to MS analysis. Peptides were analyzed using a home-made C18 analytical column (75 µm i.d. × 25 cm, ReproSil-Pur 120 C18-AQ, 1.9 µm (Dr. Maisch GmbH, Germany)). The mobile phases

consisted of Solvent A (0.1% formic acid) and Solvent B (0.1% formic acid in 80% acetonitrile). The peptides were eluted using the following gradient: 2-5% B in 2 minutes, 5-35% B in 100 minutes ,35-44% B in 6 minutes, 44-100% B in 3 minutes, 100% B for 10 minutes, at a flow rate of 200 nL/min.

For DDA experiments, the resolution of full MS scans was set as 60000 at m/z 200, the AGC target was set as 4e5 with a maximum injection time of 50 ms. The scan range was set as 400-1200 m/z. For MS2, the Normalized AGC target was set as 100% with a resolution of 15,000 and maximum injection time of 22 ms, and the NCE was set as 30%. The cycle time was set as 2 seconds.

Data independent acquisition (DIA) analysis was set as three full MS scans, and each full MS scan was followed by 20 MS2 windows. The first 20 windows were set as shown in Supplementary data table 8; the second 20 windows were set as shown Supplementary data table 9; and the third was set as shown Supplementary data table 10. The resolution of full MS scans was set as 120000 at m/z 200 and full MS Normalized AGC target was 100% with a maximum injection time of 50 ms, and a scan range was of 400-1200. The resolution of MS2 was set as 30000 with a maximum injection time of 54 ms, and the NCE was set as 32%.

Data Processing

DIA data analysis was performed using Spectronaut (version 14.7, Biognosys, Zurich, Switzerland). High precision iRT calibration was employed. The library was generated by the search engine platform Pulsar in Spectronaut (version 14.7, Biognosys, Zurich, Switzerland) using default settings, and DIA data were analyzed using default settings disabling the PTM localization filter. Mass tolerance/accuracy for precursor and fragment identification was set to default settings. FDR at the peptide and protein level was set to 1% using a mutated decoy model. For matching of DIA data to the spectral library, the applied mass and retention time tolerances (for both MS1 and MS2) are dynamic based on the m/z of the targeted ion and the retention time of the scan. The calibration was performed for each run individually. The values of iBAQ were used to quantitative analysis.

Supplementary data table 8: Settings of the first 20 windows of the MS scan for DIA analysis

m/z	z	t start (min)	t stop (min)	Isolation Window (m/z)	Normalized AGC Target (%)
403.9562	2	0	120	10.50613	100
412.7018	2	0	120	8.984924	100
420.2895	2	0	120	8.190521	100
427.8015	2	0	120	8.833588	100
435.2217	2	0	120	8.006775	100
442.1428	2	0	120	7.835449	100
449.1415	2	0	120	8.161926	100
455.9776	2	0	120	7.510254	100
462.5025	2	0	120	7.539642	100
469.2666	2	0	120	7.988556	100
476.0137	2	0	120	7.505493	100
482.5152	2	0	120	7.497589	100
489.0068	2	0	120	7.485657	100
495.4973	2	0	120	7.49527	100
502.3288	2	0	120	8.167694	100
509.1054	2	0	120	7.38559	100
515.7923	2	0	120	7.988098	100
522.9423	2	0	120	8.311951	100
529.9496	2	0	120	7.702576	100
537.0469	2	0	120	8.492188	100

Supplementary data table 9: Settings of the second 20 windows of the MS scan for DIA analysis

m/z	z	t start (min)	t stop (min)	Isolation Window (m/z)	Normalized AGC Target (%)
544.5246	2	0	120	8.463196	100
551.7519	2	0	120	7.991333	100
558.9966	2	0	120	8.497986	100
566.2987	2	0	120	8.106323	100
573.6986	2	0	120	8.693542	100
581.1627	2	0	120	8.234558	100
588.5497	2	0	120	8.53949	100
596.3158	2	0	120	8.992615	100
604.7009	2	0	120	9.77771	100
613.4788	2	0	120	9.777954	100
622.3413	2	0	120	9.947144	100

631.3462	2	0	120	10.06268	100
640.3571	2	0	120	9.959106	100
649.5827	2	0	120	10.492	100
658.9945	2	0	120	10.33167	100
668.4948	2	0	120	10.66895	100
678.3311	2	0	120	11.00366	100
688.5067	2	0	120	11.3476	100
699.021	2	0	120	11.68085	100
710.0473	2	0	120	12.37189	100

Supplementary data table 10: Settings of the third 20 windows of the MS scan for DIA analysis

m/z	z	t start (min)	t stop (min)	Isolation Window (m/z)	Normalized AGC Target (%)
721.2844	2	0	120	12.10229	100
732.6165	2	0	120	12.56189	100
744.5228	2	0	120	13.25073	100
757.0234	2	0	120	13.75037	100
770.527	2	0	120	15.25696	100
784.5499	2	0	120	14.78882	100
798.5827	2	0	120	15.27673	100
813.0505	2	0	120	15.65894	100
827.7426	2	0	120	15.7251	100
843.2513	2	0	120	17.2923	100
859.7603	2	0	120	17.72589	100
877.4602	2	0	120	19.67383	100
897.1549	2	0	120	21.71552	100
918.4784	2	0	120	22.93158	100
941.9666	2	0	120	26.04468	100
968.4867	2	0	120	28.99554	100
1000.831	2	0	120	37.69305	100
1041.069	2	0	120	44.78284	100
1089.265	2	0	120	53.60974	100
1157.785	2	0	120	85.42993	100

Results

SDS PAGE indicated that C_4 species had lower abundances of ribulose-1,5-bisphosphate carboxylase-oxygenase (RubisCO) and higher abundances of PPDK and PEPC than C_3 and C_3-C_4 intermediate species (**Supplementary data Fig. 18**). On average, 6,469 proteins (from 6,205 in Fram to 6,640 in Ftri) were detected in each species. For the following analysis, only proteins detected with a unique ID in at least

15 samples were retained, which resulted in 4,908 proteins. We found that the protein abundances of 4,908 proteins across six replicates from a same species showed higher correlations than those between species (Supplementary data Fig. 19), implying the reliability of sampling and protein quantifications. All proteins were clustered into 10 patterns according to their profiles among the five *Flaveria* species using Kmeans (Supplementary data Fig. 20). Interestingly, in line with the Kmeans analysis of transcript abundances, C_4 genes were primarily classified into one single pattern, in which the C_4 species Ftri showed the highest abundances. Similarly, photorespiratory genes were mainly clustered into one pattern, in which C_4 species had the lowest abundances (Supplementary data Fig. 20). C_4 genes had higher protein abundances in the C_4 species Ftri than in other species (Supplementary data Fig. 21), whereas photorespiratory genes had lower protein abundances in the C_4 species then other species (Supplementary data Fig. 22), consistent with previous reports [24].

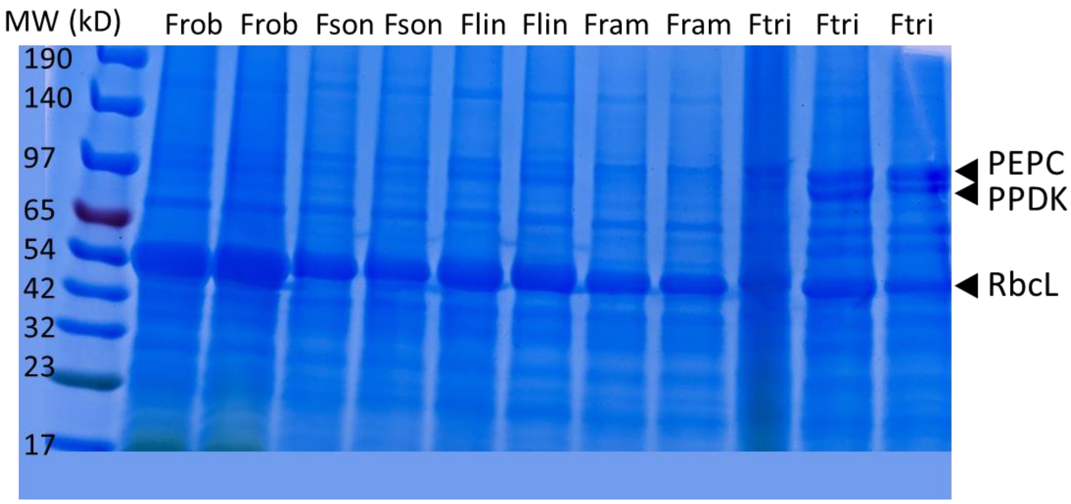

Supplementary data Fig. 18: SDS PAGE of the total proteins of *Flaveria* leaves
Total proteins were isolated from mature leaves and separated with 12% SDS PAGE on the basis of same leaf area. Two or three replicates were run for each species. (Abbreviations: RubisCO: ribulose-1,5-bisphosphate carboxylase-oxygenase; PEPC: phospho*enol*pyruvate carboxylase; PPDK, pyruvate/orthophosphate dikinase)

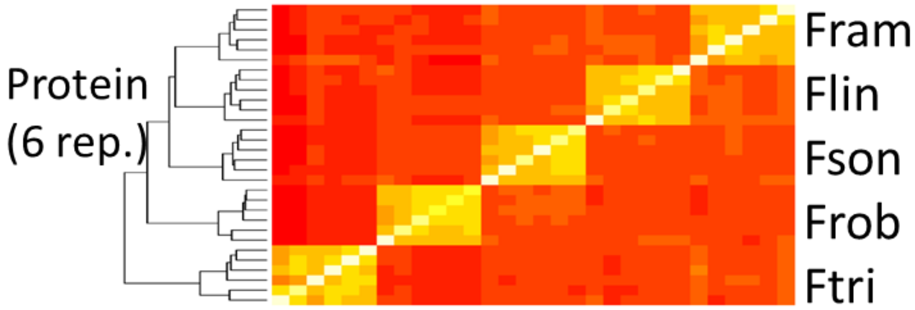

Supplementary data Fig. 19: Pearson correlation of protein datasets of *Flaveria* species in protein abundances

Heatmap showing protein abundances in log2 transformed label free quantification (LFQ) for the five *Flaveria* species. Note that six biological replicates of the same species are clustered as one branch.

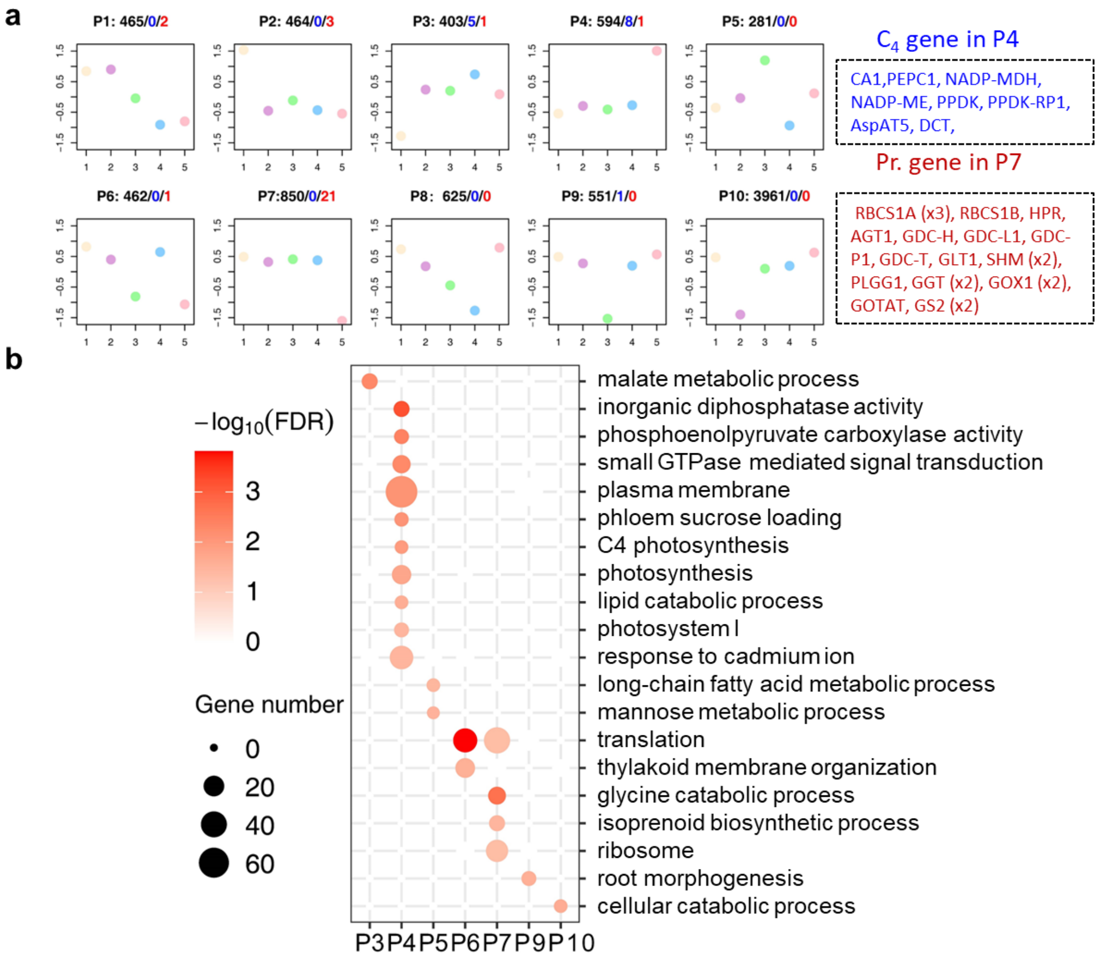

Supplementary data Fig. 20: Ten evolutionary patterns of the detected genes based on protein abundances

(a) Genes were clustered into 10 patterns according to their protein profiles within the five *Flaveria* species. Genes were clustered using Kmeans based on the mean protein abundances in Intensity-Based Absolute Quantification (iBAQ) from six biological replicates for each

species. The numbers on the top of each pattern represent: black for all genes, blue for C_4 genes and red for photorespiratory genes. (b) Enriched gene ontology (GO) terms of genes from each pattern. Enriched GO terms were calculated using Fisher's exact test with a P value threshold of 0.01 adjusted applying Benjamini & Hochberg method. Intensity-Based Absolute Quantification (IBAQ)

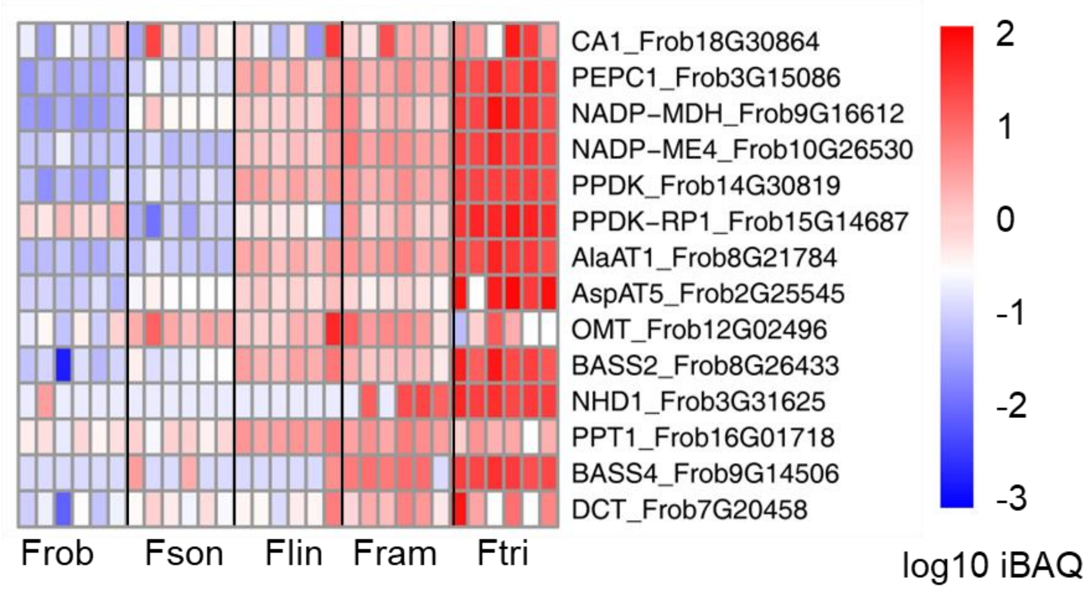

Supplementary data Fig. 21: Protein abundances of C_4 genes

Heatmap showing the log2 transformed protein abundances in label free quantification (LFQ). (Abbreviations: CA1: carbonic anhydrase 1; PEPC1: phosphoenolpyruvate carboxylase 1; NADP-MDH: NADP-dependent malate dehydrogenase; NADP-ME4: NADP-dependent malic enzyme 4; PPDK: pyruvate/orthophosphate dikinase; PPDK-RP, PPDK regulatory protein; AlaAT1, alanine aminotransferase 1; AspAT5, aspartate aminotransferase 5; OMT, oxaloacetate/malate transporter, or dicarboxylate transporter 1 (DiT1); BASS2, bile acid sodium symporter 2; NHD1, sodium: hydrogen antiporter 1; PPT1, phosphate/phosphoenolpyruvate translocator 1; BASS4, bile acid sodium symporter 4, and DCT: dicarboxylate transporter 2.1 (DiT2.1))

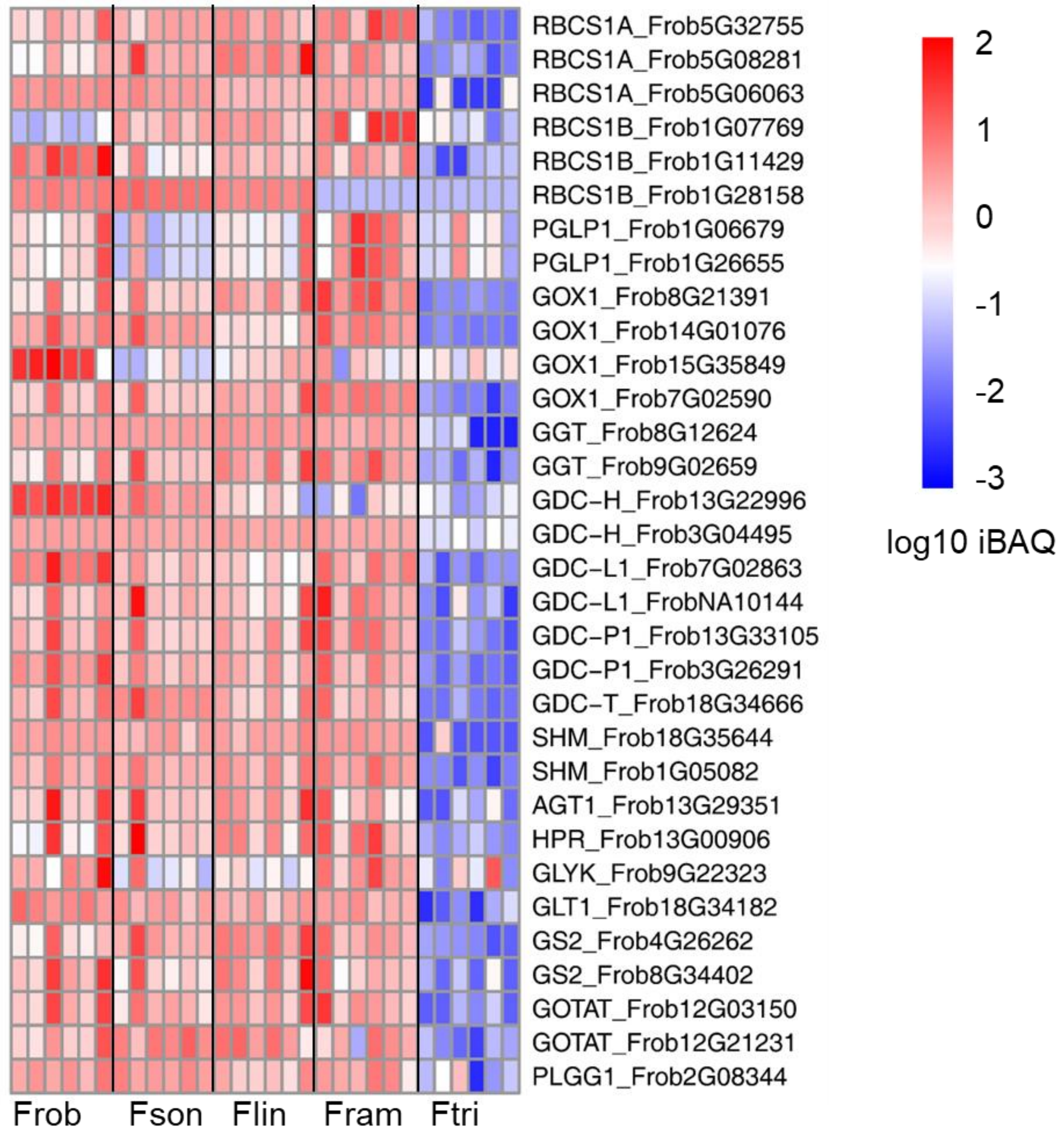

Supplementary data Fig. 22: Protein abundance of photorespiratory genes

Heatmap showing the log 2 transformed protein abundance in label free quantification (LFQ). (Abbreviations: RBCS1A: Ribulose-1,5-bisphosphat-carboxylase/-oxygenase (RubisCO) small subunit 1 A/B; RBSC1B: RubisCO small subunit B; PGLP1: 2-phosphoglycerate phosphatase1, GOX: glycolate oxidase; GDC-H: GDC-L, glycine decarboxylase complex (GDC) subunit L, GDC-P: GDC subunit P; GDC-T: GDC subunit T; SHM: serine hydroxymethyltransferase; AGT1: serine glyoxylate aminotransferase, HPR: hydroxypyruvate reductase ; GLYK: D-glycerate 3-kinase; GLT1: glutamate synthase 1; GS2: glutamine synthase 2; GOTAT: glutamine oxoglutarate aminotransferase; PLGG: chloroplastidic glycolate/glycerate transporter.)

Supplementary Data 12: Comparison of protein-to-transcript ratio

Methods

We compared the protein-to-mRNA ratio (PTR) between genes across five *Flaveria* species. Low PTR genes and high PTR genes were defined as genes with PTR values less than the mean PTR minus standard deviation (SD) and higher than the mean PTR plus SD, respectively. The remaining genes were defined as moderate PTR genes. Enriched gene ontology (GO) terms were calculated using Fisher's exact test as mentioned above. The protein abundances and transcript abundances of *Arabidopsis thaliana* (Atha) were inferred from a previously study [25]. To compare PTR between photosynthetic genes and the remaining genes, photosynthetic genes were defined as those with gene ontology of GO:0015979.

Results

An average of 181 low PTR genes (from 138 to 238) and 418 high PTR genes (from 372 to 469). The low PTR genes were enriched in gene ontology (GO) of photosynthesis and photosynthesis related GO terms, including chloroplast and, PSII (Supplementary data Fig. 23). Additionally, photosynthetic genes showed a trend towards lower PTR (Supplementary figure 6), consistent with an early study in *Arabidopsis thaliana* (Atha), which showed that photosynthesis related genes had significantly lower PTRs than other genes in photosynthetic functional leaf tissues [25], but not in old leaves (Supplementary data Fig. 24) or other tissues [25].

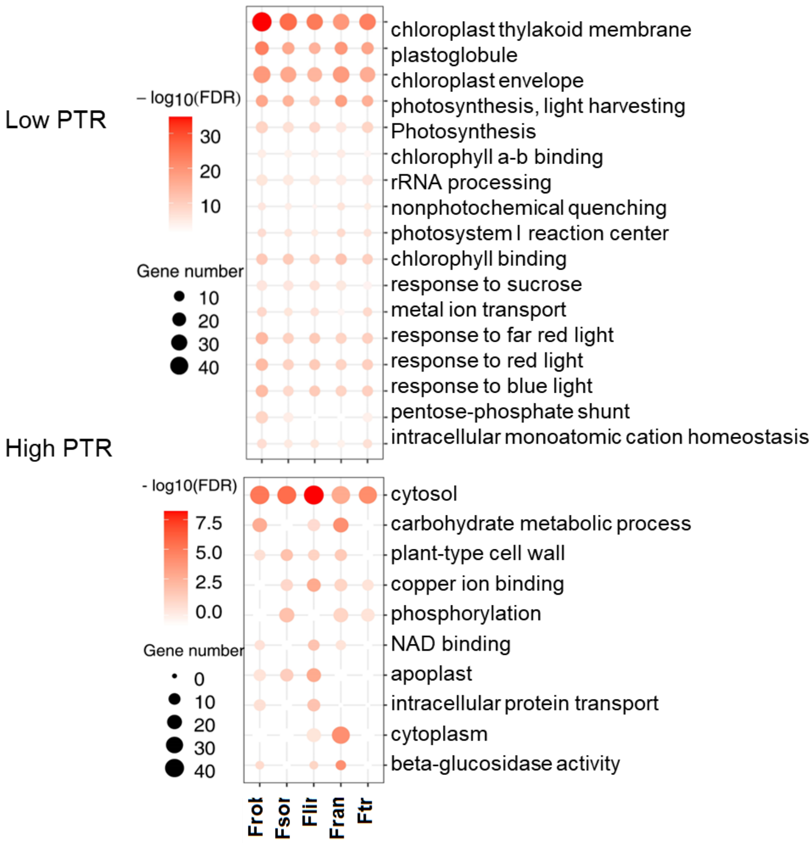

Supplementary data Fig. 23: High and low PTR genes and their enriched functions in five *Flaveria* species

Enriched gene ontology of low PTR and high PTR genes, which were calculated using Fisher's exact test with a *P*-value threshold of 0.001 (Benjamini & Hochberg adjusted). (Abbreviation: PTR: protein-to-mRNA ratio.)

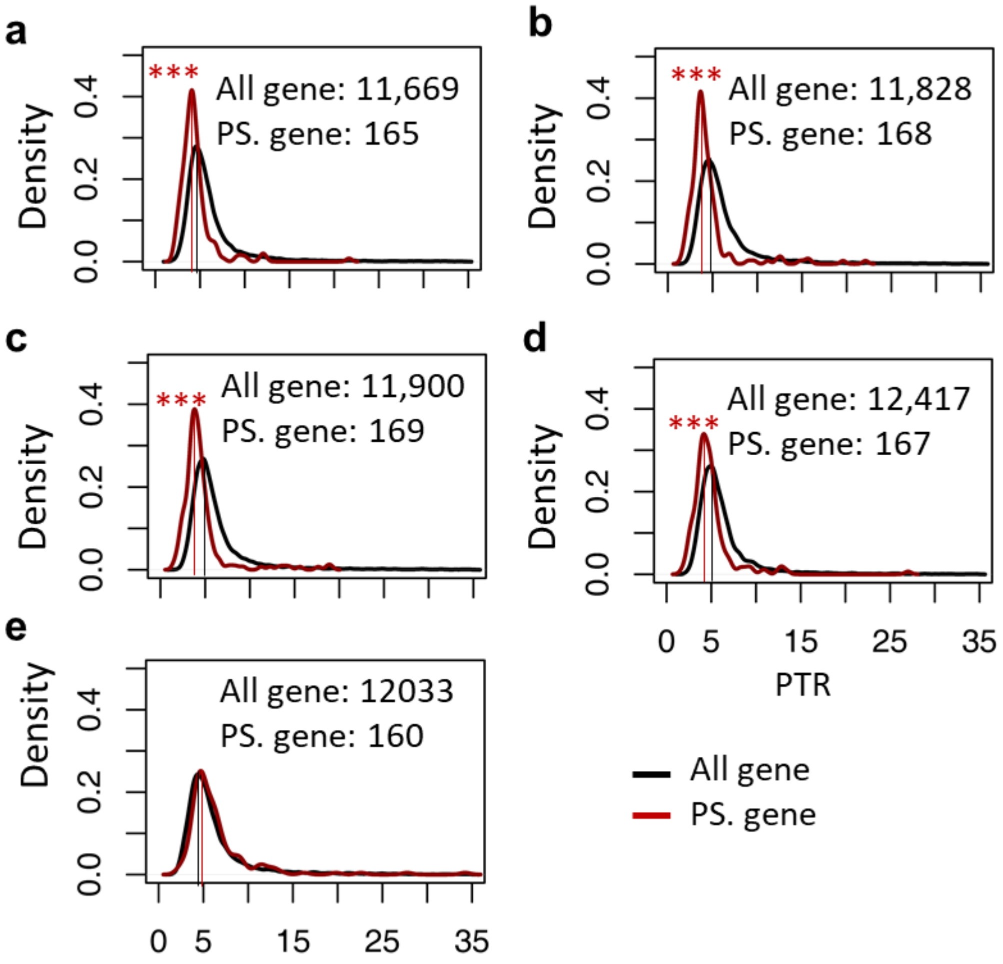

Supplementary data Fig. 24: **Photosynthetic genes have lower PTRs than other genes in photosynthetic active leaves of *Arabidopsis thaliana***

The distribution of PTRs of all detected genes and photosynthetic related genes in (a) 1st cauline leaf, (b) distal part of rosette leaf 7, (c) proximal part of rosette leaf 7, (d) petiole of rosette leaf 7, and (d) senescent leaf. Photosynthetic genes have annotated gene ontology of GO:0015979. The number of all detected genes and photosynthetic related genes in each leaf sample are showed within the panel. Figures were drawn based on data in supplementary data 4 from a prior study [25]. (Abbreviations: PTR: protein-to-mRNA ratio; PS. Photosynthesis.)

Supplementary Data 13: Prediction of *cis*-regulatory elements using ATAC-seq

Methods

Nucleic isolation, library construction sequencing, peaks calling and Tn5 hypersensitive site (THS) were illustrated in the Methods of the manuscript. To assess the consistency of the two biological replicates, the irreproducibility discovery rate (IDR) analysis was performed using the idr package (v2.0.4) following the ENCODE guidelines. The IDR analysis ensures that the peaks called in the two biological replicates are reproducible and reliable. The IDR threshold was set to 0.05 to filter out irreproducible peaks.

Results

Approximately 30% ATAC-seq raw reads were uniquely mapped to the genome sequences of Ftri (Supplementary data Fig. **25**). We calculated the Fraction of Reads in Reproducible Peaks (FRiPs) from the IDR analysis. The results show that the FRiPs were 15% for replicate 1 (Ftri1) and 16% for replicate 2 (Ftri2). We used "idr" to perform irreproducibility discovery rate analysis to assess the consistency of the two biological replicates of ATAC-seq data from Ftri. As a result, we obtained 14,333 peaks at the IDR threshold of 0.05 (Supplementary data Fig. 25). We also calculated the correlation of mapped read counts from union peak regions (204,265 peaks) across two distinct biological replicates of Ftri. Results showed a high Pearson correlation coefficient of 0.90 between the two replicates. (Supplementary data Fig. 26).

We found 1,471,751 occurrences of 277 known plant motifs in the 14,333 peaks. Among these, 1,858 occurrences of 117 motifs were from the ACRs near C_4 genes (including the regions 3 kb upstream start codons, 3kb downstream of stop codons, and the gene bodies). To further determine which motif was associated with the C_4 genes more than expected by chance, we performed both Monte Carlo permutation test and Fisher's exact test (See Methods in the manuscript). The motif enrichment

results were largely consistent between these two methods (Supplementary data table 11).

a

Replicate number	Total reads	Mapping rate	Unique mapping
Ftri_R1	25,291,260	97.7%	34.0%
Ftri_R2	31,522,521	97.3%	29.1%

b

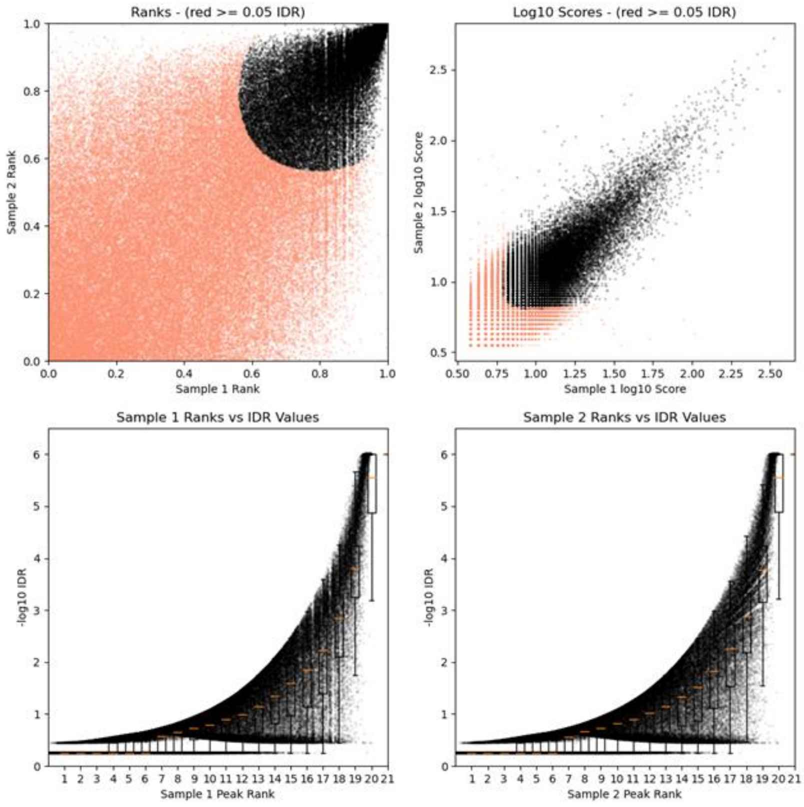

Supplementary data Fig. 25: Analysis of ATAC-seq data in Ftri

(a) Statistic of two replicates of ATAC-seq in Ftri. (b) Replicate consistency of ATAC-seq data for Ftri using IDR analysis. This figure presents the results of the Irreproducible Discovery Rate (IDR) analysis of two biological replicates of ATAC-seq data from Ftri. The upper left panel displays a scatterplot of ranks versus ranks, and the upper right panel shows log10 scores versus ranks, with red designating regions surpassing the IDR threshold. Regions having an IDR value greater than or equal to 0.05 are indicated in red. The lower panels depict rank distribution plots for each sample against the negative logarithm of IDR values, with black bars representing the number of peaks falling within each rank-IDR bin.

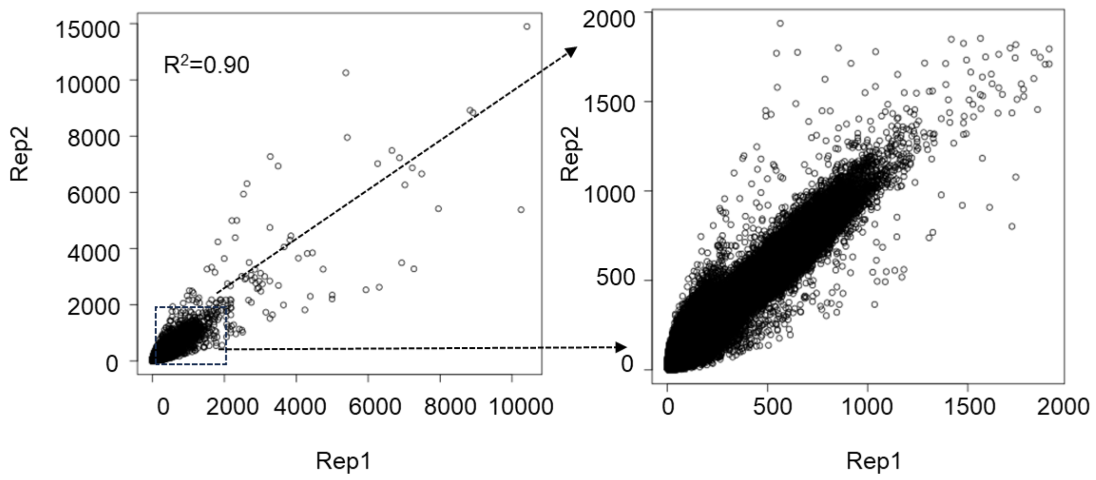

Supplementary data Fig. 26: Replicate consistency analysis of ATAC-seq data in Ftri

Plots illustrate the correlation of mapped read counts from union peak regions across two biological replicates of Ftri. The regions with counts within 2000 are highlighted for clarity.

Supplementary data table 11: Enriched motifs related to C_4 genes either from Monte Carlo permutation test or from Fisher's exact test

Motif	TF family	#Total motif in background	# Motif in background	# Total motif C_4 gene	Motif in C_4	FDR (1000 times of Permutation)	Pvalue (Fisher.test)	Odds Ratio
TFmatrixID_0771	ERF	1471751	16826	1858	49	0	1.6E-07	2.31
TFmatrixID_0748	ERF	1471751	36710	1858	76	0	4.1E-05	1.64
TFmatrixID_0777	ERF	1471751	34203	1858	67	0.001	4.7E-04	1.55
TFmatrixID_0499	MIKC_MADS	1471751	3557	1858	11	0.001	3.4E-03	2.45
TFmatrixID_0782	ERF	1471751	24802	1858	54	0.001	1.1E-04	1.72
TFmatrixID_0664	ERF	1471751	51305	1858	92	0.001	8.2E-04	1.42
TFmatrixID_0719	ERF	1471751	40625	1858	72	0.007	3.6E-03	1.40
TFmatrixID_0772	ERF	1471751	27689	1858	50	0.007	8.4E-03	1.43
TFmatrixID_0724	ERF	1471751	15570	1858	32	0.008	4.5E-03	1.63
TFmatrixID_1270	MYB_related	1471751	18663	1858	34	0.022	1.9E-02	1.44
TFmatrixID_1259	MYB_related	1471751	448	1858	3	0.029	1.0E-02	5.31
TFmatrixID_0798	BBR-BPC	1471751	1172	1858	4	0.029	3.2E-02	2.70
TFmatrixID_0973	C2H2	1471751	22956	1858	39	0.034	3.7E-02	1.35
TFmatrixID_0892	Dof	1471751	9574	1858	19	0.042	2.9E-02	1.57
TFmatrixID_0023	ERF	1471751	57091	1858	91	0.043	1.8E-02	1.26
TFmatrixID_1238	MYB_related	1471751	573	1858	3	0.047	1.9E-02	4.15
TFmatrixID_1378	B3	1471751	979	1858	4	0.048	1.9E-02	3.24
TFmatrixID_1214	MYB	1471751	28474	1858	41	0.048	2.0E-01	1.14
TFmatrixID_0055	ERF	1471751	421	1858	2	0.048	5.0E-02	3.76
TFmatrixID_1370	Nin-like	1471751	5510	1858	10	0.049	1.2E-01	1.44
TFmatrixID_1126	LBD	1471751	3265	1858	7	0.049	7.0E-02	1.70
TFmatrixID_0743	ERF	1471751	47391	1858	72	0.049	6.6E-02	1.20
TFmatrixID_1004	CPP	1471751	55	1858	1	0.062	3.4E-02	14.40

Note: FDR were adjusted with Benjamini-Hochberg method.

Supplementary Data 14: ERF *cis*-regulatory elements were abundant in photosynthesis related genes of different C_4 species

Methods

ATAC-seq data of *Zea mays* mesophyll cell and bundle sheath cells were obtained from a previous study [26]. The data of TF binding sites (TFBS) of *Zea mays*, *Setaria italica* and *Sorghum bicolor* were from a prior study [27]. To investigate the enriched CREs of C_4 genes in the C_4 species Ftri, the promoter region (3k bps upstream of the start codon) of C_4 genes were compared with the rest of the genes using findMotifsGenome.pl within Hommer [28] (v4.11.1) with default parameters.

Results

ERF CREs were also the most abundant CREs in *Zea mays* (corn), *Setaria italica* and *Sorghum bicolor* (Supplementary data Fig. 27 and Supplementary data Fig. 28).

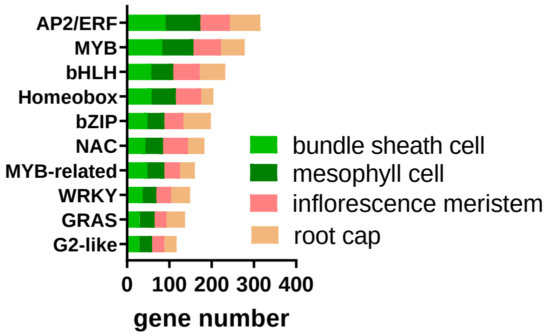

Supplementary data Fig. 27: A large number of ERF TFs show positive chromatin accessibility in different tissues of *Zea mays*

Bar plots showing the profiles of chromatin accessibility of TFs from different types of tissues in *Zea mays*. The top ten TFs with the greatest number of genes showing chromatin accessibility are shown here. A large number of ERF TFs show positive chromatin accessibility in different tissues, especially in the mesophyll and bundle sheath. TF frequency data used for drawing this bar plot are from a previous study [26] which are based on single cell ATAC-seq. (Abbreviations: MC: mesophyll cell; BSC: bundle sheath cell.)

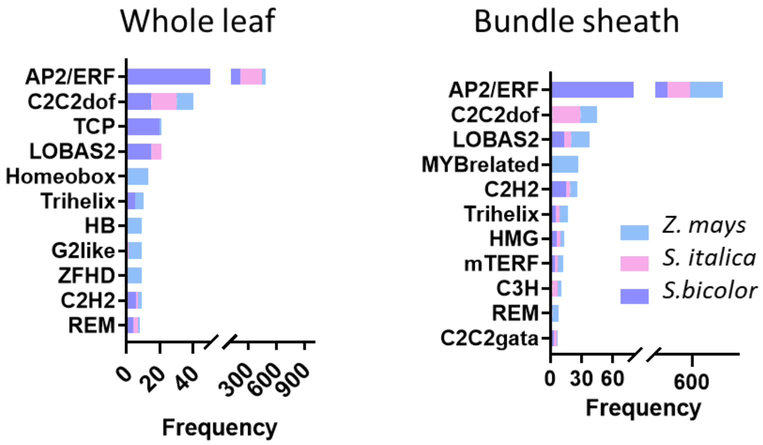

Supplementary data Fig. 28: ERF TF binding sites are abundant in photosynthesis related genes

Bar plots showing the frequency of different TF binding site (TFBS) in photosynthesis related genes in the chromosome regions of the three C_4 species, *i.e.*, *Zea mays* (*Z. mays*), *Setaria italica* (*S. italica*) and *Sorghum bicolor* (*S. bicolor*). Photosynthesis related genes include genes in C_4 pathway, photorespiratory pathway and Calvin-Benson-Bassham cycle. The top 10 most frequently known TFBS are showed. The data of TFBS used for drawing bar plots here are from a previous study [27], in which the chromosome open regions were computed based on DNase-seq.

Supplementary Data 15: Construction of gene regulatory networks

Methods

We constructed genome-wide co-regulatory networks (GRNs) for each of the five *Flaveria* species, which based on the gene expression profiles of at least 18 RNA-seq datasets either from a previous work [29] (for Frob, Fson, Fram and Ftri) or generated in the current study (for Flin) (Supplementary data table 12). The GRNs were constructed following a previously decribed method [29] . Specifically, genome-wide gene regulatory networks (GRNs) of five *Flaveria* species were constructed independently through application of the CMIP software package [30]. The CMIP package implements a path consistency algorithm based on the conditional mutual information which is specifically designed to predict direct regulation between genes [31]. Based on the gene expression table (the only input), the method generates a zero-order network by computing mutual information (MI), and indirect relationships were removed considering the CMI, which resulted in a first-order network. We implemented a *P*-value determination in the CMIP package based on a permutation test. Specifically, we shuffled the expression level of each gene 2000 times, generating 2,000 null datasets. Thereafter, the *P*-value was defined as the ratio of number of null datasets to 2000 whose CMI values were higher than that calculated from the original dataset. Here, a *P*-value of 0.001 was used as the cutoff. The C_4GRN was obtained by retaining the GRN of the C_4 genes and their co-regulated TFs (thereafter, C_4TFs) from the genome-wide GRN.

In the original CMIP package, the cutoff of gene-gene partial Pearson correlation coefficient (PCC) was estimated as the inflection point of the exponential-function-fitted curve with the relationship between edge number and PCC values [30]. Considering that we mainly focused on the C_4GRN, we revised this computational procedure to determine the proper PCC. Specifically, we compared the relative abundance of all TF families (TF frequency) of the C_4GRN obtained using different PCC cutoffs, increasing from 0.1 to 0.9 with a step size of 0.1. We found that the TF

frequency maintained a high degree of similarity under different PCC cutoffs ranging from 0.1 to 0.7 (Supplementary data Fig. 29). We found that a PCC of 0.5 represented the inflection point in the curve of relationship between edge number of C_4GRNs and PCC cutoffs (Supplementary data Fig. 30). We thus selected 0.5 to be the PCC cutoff for GRN construction.

Results

We constructed a genome-wide co-regulatory network (GRN) of the five *Flaveria* species based on the gene expression profiles of at least 18 RNA-seq datasets either from a previous work [29] or generated in the current study (Supplementary data table 12).

Previously, we constructed C_4GRN for Frob, Fson, Fram and Ftri using the same RNA-seq datasets, whereas genes were annotated based on the transcript of Fram [29] (transcriptome-based C_4GRN). We compared the C_4GRN constructed here (genome-based C_4GRN) with transcriptome-based C_4GRN for the four species. The total gene number and TFs were enhanced due to the improved gene annotations, which resulted in increased C_4TFs across all the species (Supplementary data Fig. 31a), nevertheless, the trends of the number of C_4TFs along evolution (from C_3 to C_4) were consistent between the two studies[29] . The updated C_4GRN demonstrated that 1/3 C_4TFs were species specific, which was lower than the transcriptome-based C_4GRN (Supplementary data Fig. 31b and c), but still consistent with previous report [29] that a large number of TFs were species specific. In addition, among the top 20 TF families with the greatest number of C_4TFs, 15 of them were common across the two studies (Supplementary data Fig. 31d and e). On the other hand, the genome-based GRNs were improved as following (1) with the genome annotation, we improved the annotation of TFs for the five Flaveria species sequenced here, (2) combined with *cis*-elements information, we filtered out C_4 genes co-regulated TFs with no predicted cognate *cis*-regulatory elements on the promoter. These improvements enabled us to find out that ERF TFs were more abundant in C_4GRNs in the C_4 species than in other species (Fig. 5d and Supplementary figure 10 in the manuscript).

While the total number of ERF were comparable in all the five *Flaveria* species, C_4GRN of the C_4 species Ftri showed more ERF TFs than those in other *Flaveria* species, specifically, 27 ERF TFs were predicted in Ftri C_4GRN (Fig. 5e in the manuscript) compared to 11 in Frob C_4GRN (Supplementary data Fig. 32 a). When only one copy of *CA1*, *PEPC1* and *PEPC-k* were analyzed for C_4GRN in the C_4 species Ftri, 22 ERF TFs were co-regulated with 13 C_4 genes (Supplementary data Fig. 32b).

We performed electrophoretic mobility shift assays (EMSA) and transient expression experiments to verify the regulation of ERF transcription factors on C_4 genes in Ftri (C_4) (Figs. 5 f-i in the manuscript). The primers were list in Supplementary data table 13.

Supplementary data table 12: RNA-seq datasets used for constructing gene regulatory networks for the five *Flaveria* species

**Species**	**low CO_2**	**low CO_2**	**low CO_2**	**ABA**	**high light**	**# total Samples**
	2 weeks	4 weeks	6 months	3 hours	2 weeks	
**Frob**	6	6	4	6	6	**28**
**Fson**	6	6	4	6	n.a	**22**
**Flin**	6	6	n.a	n.a	6	**18**
**Fram**	6	6	n.a	6	6	**24**
**Ftri**	6	6	n.a	6	6	**24**

Note: n.a: not available

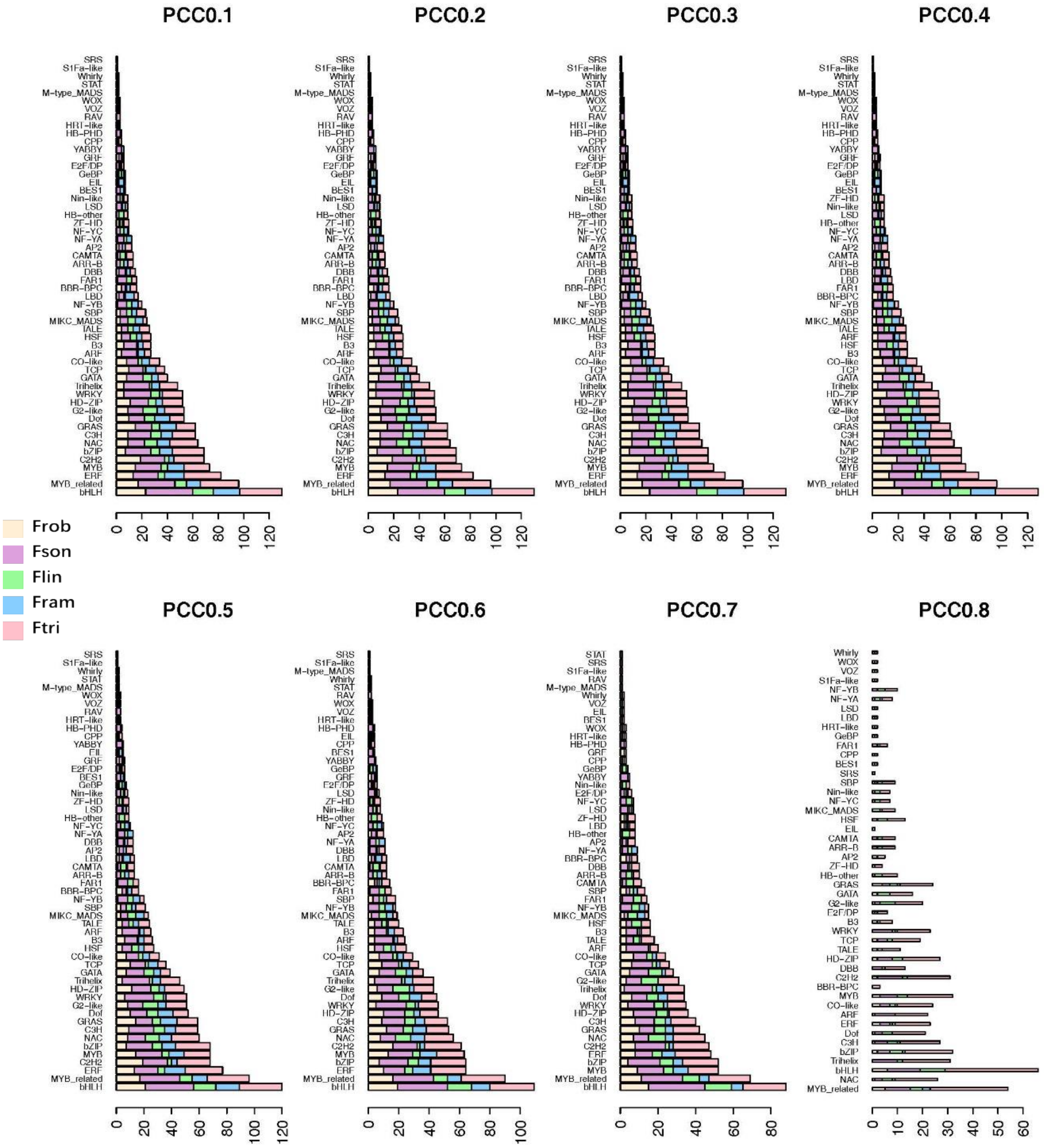

Supplementary data Fig. 29: Distributions of C$_4$TFs in each TF family for each *Flaveria* species under different PCCs cutoffs

Bar plots showing the TFs number for each TF family across four *Flaveria* species under different thresholds of PCC cutoffs ranging from 0.1 to 0.8. The X axes display the number of TFs for each TF family.

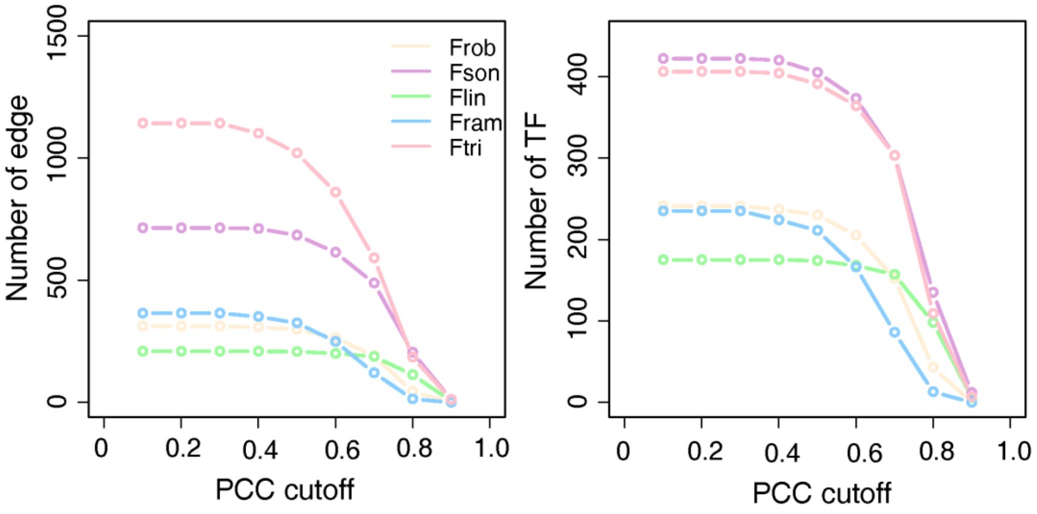

Supplementary data Fig. 30: The number of edges and TFs of C_4GRN under different PCC cutoffs

Curves showing the distributions of C_4GRN edges (interaction between C_4 genes and TFs) in five *Flaveria* species under PCC cutoffs ranging from 0.1 to 0.9. The number of edges shows an inflection point at a PCC of 0.5.

a

Items	Study	Frob (C_3)	Fson (C_3-C_4)	Fram (C_3-C_4)	Ftri (C_4)
# Genes	Trans	15,005	14,677	17,784	14,761
	Geno	26,147	24,744	19,599	22,377
# Total TF	Trans	1,604	1,569	1,968	1,608
	Geno	2,430	2,366	1,992	2,066
# TF in C_4GRN (C_4TF)	Trans	185	220	114	196
	Geno	191 (166)$^{\alpha}$	366 (300)$^{\alpha}$	175 (160)$^{\alpha}$	323 (280)$^{\alpha}$
# Species specific C_4TF	Trans	129	143	66	125
	Geno	61 (53)$^{\alpha}$	137 (109)$^{\alpha}$	50 (43)$^{\alpha}$	121 (97)$^{\alpha}$
# C_4TF shared with Ftri	Trans	23	37	26	196
	Geno	74 (61)$^{\beta}$	162 (126)$^{\beta}$	78 (71)$^{\beta}$	324 (280)$^{\beta}$

note: α the number of orthologous group

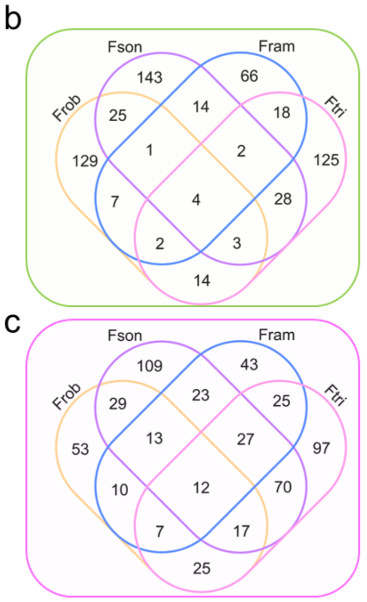

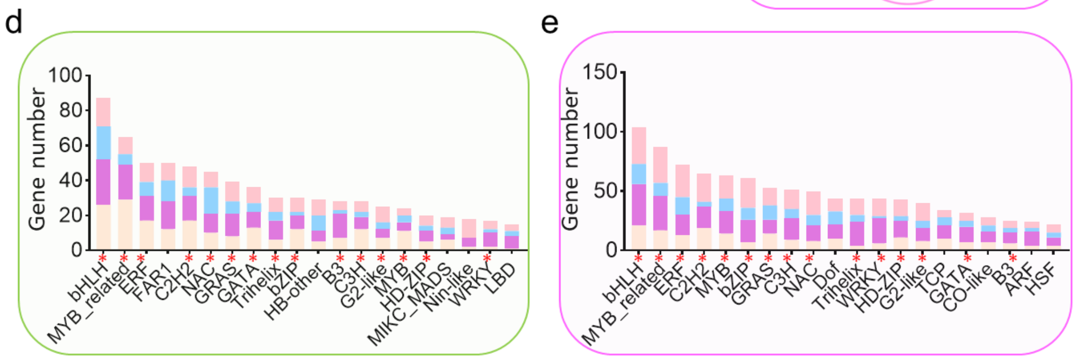

Supplementary data Fig. 31: Comparison of C_4 GRN based on RNA-seq data with either genome annotation or transcript annotation.

(a) Statistics of GRNs based on transcript-based annotation (Trans. for short) from a previous study [29] and genome-based annotation (Geno. for short) from this study. α and β represent the number of orthologous groups that the under-studied TFs belonging to. (b) and (c) Venn diagram showing the intersection between TFs across four *Flaveria* species from transcript-based C_4GRN and genome-based C_4GRN, respectively. The numbers in (c) represents the number of orthologous groups instead of genes. (d) and (e) Bar plots showing the frequency of the top 20 TF families with the greatest number of genes in C_4GRNs from transcript-based GRN and genome-based GRN respectively. TF families shared between transcript-based GRN and genome-based GRN are marked with red a star.

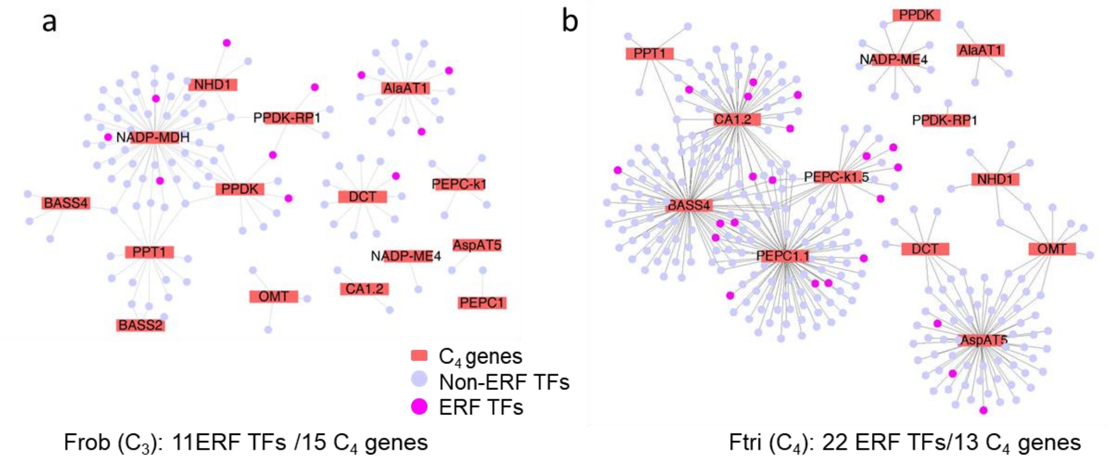

Supplementary data Fig. 32: The C_4GRN of Ftri and Frob

(a)The C_4GRN of the C_3 species Frob. The C_4GRN of the Ftri (C_4) was shown in Fig. S6 in the manuscript. (c) C_4GRN of Ftri including only one copy for *CA1*, *PEPC1*, and *PEPC-k1* respectively.

Supplementary data table 13: Primer used in experimental validation of the function of ERF TFs in Ftri

**Constructs for Protein Purification in *E. coli* for EMSAs**	
Ftri15G25371(ERF12)_pET_GST_F	ggcggtggcggttctatgGCTTCTCCGGCGC
Ftri15G25371(ERF12)_pET_GST_R	tgccgccgccaccAAGCCAAATGGGTGGAGGC
Ftri17G17198(ERF1)_pET_GST_F	ggcggtggcggttctatgCATCACAACATTTTCCGC
Ftri17G17198(ERF1)_pET_GST_R	tgccgccgccaccAACTAGAACTACTACTGCTACAAGT
Ftri1G11197(ERF57)_pET_GST_F	ggcggtggcggttctatgGCTTCTGCAACAATGGAG
Ftri1G11197(ERF57)_pET_GST_R	TGCCGCCGCCACCTAGAGCTTCCCAATCAATCTCATACG

Ftri13G23465(ERF61)_pET_GST_F	ggcggtggcggttctatgGCGAAAACAGGGGTATCA
Ftri13G23465(ERF61)_pET_GST_R	TGCCGCCGCCACCATTTGCTAAAACTTCCCAAATCAAC
**Constructs for transient transcription assay in Zmay**	
FtCA-pGreenII_F	gtcgacggtatcgataTTTTAATATTGATATTTTTTTAAAACAGTTTG
FtCA-pGreenII_R	cgctctagaactagtgAAATTGTATATGAGTAATAATATAAGAAATAAAACTATG
FtPEPC1.1-pGreenII_F	gtcgacggtatcgatTTCATTTTCAACTTTGATCCCATATAC
FtPEPC1.1-pGreenII_R	cgctctagaactagtATATTATTAAAAAAAATAATAGCTAGGTCTATCTATG
FtAspAT-pGreenII_F	gtcgacggtatcgataaATGGTAATACAAACGGTGGGTTT
FtAspAT-pGreenII_R	cgctctagaactagtgATATAAAATAGTGGGTCGGGTTTATTT
Ftri15G25371(ERF12)-pCAMBIA1300-F	tttggagagaacacgcccATGGCTTCTCCGGCGCAC
Ftri15G25371(ERF12)-pCAMBIA1300-R	acttcctcctccaccAAGCCAAATGGGTGGAGGC
Ftri13G23465(ERF61)-pCAMBIA1300-F	tttggagagaacacgcccATGGCGAAAACAGGGGTATCA
Ftri13G23465(ERF61)-pCAMBIA1300-R	acttcctcctccaccATTTGCTAAAACTTCCCAAATCAAC

References

1 Li, S. F. *et al.* The landscape of transposable elements and satellite DNAs in the genome of a dioecious plant spinach (Spinacia oleracea L.). *Mob DNA* **10**, 3, doi:10.1186/s13100-019-0147-6 (2019).

2 Seppey, M., Manni, M. & Zdobnov, E. M. BUSCO: Assessing Genome Assembly and Annotation Completeness. *Methods Mol Biol* **1962**, 227-245, doi:10.1007/978-1-4939-9173-0_14 (2019).

3 Li, B. & Dewey, C. N. RSEM: accurate transcript quantification from RNA-Seq data with or without a reference genome. *BMC Bioinformatics* **12**, 323, doi:10.1186/1471-2105-12-323 (2011).

4 Dobin, A. *et al.* STAR: ultrafast universal RNA-seq aligner. *Bioinformatics* **29**, 15-21, doi:10.1093/bioinformatics/bts635 (2013).

5 Langmead, B. & Salzberg, S. L. Fast gapped-read alignment with Bowtie 2. *Nature Methods* **9**, 357-U354, doi:10.1038/Nmeth.1923 (2012).

6 Taniguchi, Y. Y. *et al.* Dynamic changes of genome sizes and gradual gain of cell-specific distribution of C4 enzymes during C4 evolution in genus Flaveria. *Plant Genome*, e20095, doi:10.1002/tpg2.20095 (2021).

7 Min, X. J., Butler, G., Storms, R. & Tsang, A. OrfPredictor: predicting protein-coding regions in EST-derived sequences. *Nucleic Acids Res* **33**, W677-680, doi:10.1093/nar/gki394 (2005).

8 Camacho, C. *et al.* BLAST plus : architecture and applications. *BMC Bioinformatics* **10**, doi:https://doi.org/10.1186/1471-2105-10-421 (2009).

9 Emms, D. M. & Kelly, S. OrthoFinder: phylogenetic orthology inference for comparative genomics. *Genome Biol* **20**, 238, doi:10.1186/s13059-019-1832-y (2019).

10 Edgar, R. C. MUSCLE: multiple sequence alignment with high accuracy and high throughput. *Nucleic Acids Research* **32**, 1792-1797, doi:10.1093/nar/gkh340 (2004).

11 Stamatakis, A. RAxML-VI-HPC: Maximum likelihood-based phylogenetic analyses with thousands of taxa and mixed models. *Bioinformatics* **22**, 2688-2690, doi:10.1093/bioinformatics/btl446 (2006).

12 Lyu, M. J. *et al.* What Matters for C_4 Transporters: Evolutionary Changes of Phosphoenolpyruvate Transporter for C4 Photosynthesis. *Front Plant Sci* **11**, 935, doi:10.3389/fpls.2020.00935 (2020).

13 Xu, Z. & Wang, H. LTR_FINDER: an efficient tool for the prediction of full-length LTR retrotransposons. *Nucleic Acids Research* **35**, W265-W268, doi:10.1093/nar/gkm286 (2007).

14 Ellinghaus, D., Kurtz, S. & Willhoeft, U. LTRharvest, an efficient and flexible software for de novo detection of LTR retrotransposons. *BMC Bioinformatics* **9**, 18, doi:10.1186/1471-2105-9-18 (2008).

15 Ou, S. & Jiang, N. LTR_retriever: A Highly Accurate and Sensitive Program for Identification of Long Terminal Repeat Retrotransposons. *Plant Physiol* **176**, 1410-1422, doi:10.1104/pp.17.01310 (2018).

16 Kaessmann, H., Vinckenbosch, N. & Long, M. RNA-based gene duplication: mechanistic and evolutionary insights. *Nat Rev Genet* **10**, 19-31, doi:10.1038/nrg2487 (2009).

17 Tan, S. *et al.* DNA transposons mediate duplications via transposition-independent and -dependent mechanisms in metazoans. *Nat Commun* **12**, 4280, doi:10.1038/s41467-021-24585-

9 (2021).

18 Xiao, H., Jiang, N., Schaffner, E., Stockinger, E. J. & van der Knaap, E. A retrotransposon-mediated gene duplication underlies morphological variation of tomato fruit. *Science* **319**, 1527-1530, doi:10.1126/science.1153040 (2008).

19 Tan, S. J. *et al.* LTR-mediated retroposition as a mechanism of RNA-based duplication in metazoans. *Genome Research* **26**, 1663-1675, doi:10.1101/gr.204925.116 (2016).

20 Zhu, Z., Tan, S., Zhang, Y. & Zhang, Y. E. LINE-1-like retrotransposons contribute to RNA-based gene duplication in dicots. *Sci Rep* **6**, 24755, doi:10.1038/srep24755 (2016).

21 Hu, Y. *et al.* Rapid Genome Evolution and Adaptation of Thlaspi arvense Mediated by Recurrent RNA-Based and Tandem Gene Duplications. *Front Plant Sci* **12**, 772655, doi:10.3389/fpls.2021.772655 (2021).

22 Akyildiz, M. *et al.* Evolution and function of a cis-regulatory module for mesophyll-specific gene expression in the C_4 dicot Flaveria trinervia. *Plant Cell* **19**, 3391-3402, doi:10.1105/tpc.107.053322 (2007).

23 Zhu, M.-J. A. L. J. E. F. C. G. C. X.-G. Evolution of co-regulatory network of C_4 metabolic genes and TFs in the genus Flaveria: go anear or away in the intermediate species? *BioRxiv*, doi:10.1101/2020.10.02.324558 (2020).

24 Mallmann, J. *et al.* The role of photorespiration during the evolution of C_4 photosynthesis in the genus Flaveria. *Elife* **3**, e02478, doi:10.7554/eLife.02478 (2014).

25 Mergner, J. *et al.* Mass-spectrometry-based draft of the Arabidopsis proteome. *Nature* **579**, 409-414, doi:10.1038/s41586-020-2094-2 (2020).

26 Marand, A. P., Chen, Z. L., Gallavotti, A. & Schmitz, R. J. A cis-regulatory atlas in maize at single-cell resolution. *Cell* **184**, 3041-+, doi:10.1016/j.cell.2021.04.014 (2021).

27 Burgess, S. J. *et al.* Genome-Wide Transcription Factor Binding in Leaves from C_3 and C_4 Grasses. *Plant Cell* **31**, 2297-2314, doi:10.1105/tpc.19.00078 (2019).

28 Heinz, S. *et al.* Simple combinations of lineage-determining transcription factors prime cis-regulatory elements required for macrophage and B cell identities. *Mol Cell* **38**, 576-589, doi:10.1016/j.molcel.2010.05.004 (2010).

29 Lyu, M. J. *et al.* Evolution of gene regulatory network of C_4 photosynthesis in the genus Flaveria reveals the evolutionary status of C_3-C_4 intermediate species. *Plant Commun* **4**, 100426, doi:10.1016/j.xplc.2022.100426 (2023).

30 Zheng, G. *et al.* CMIP: a software package capable of reconstructing genome-wide regulatory networks using gene expression data. *BMC Bioinformatics* **17**, 535, doi:10.1186/s12859-016-1324-y (2016).

31 Zhang, X. J. *et al.* Inferring gene regulatory networks from gene expression data by path consistency algorithm based on conditional mutual information. *Bioinformatics* **28**, 98-104, doi:10.1093/bioinformatics/btr626 (2012).

32 Reyna-Llorens, I. & Hibberd, J. M. Recruitment of pre-existing networks during the evolution of C_4 photosynthesis. *Philos Trans R Soc Lond B Biol Sci* **372**, doi:10.1098/rstb.2016.0386 (2017).
