# Supplementary material for: A dominant role of transcriptional regulation during the evolution of C4 photosynthesis in Flaveria species
Source: Nat Commun. 2025 Feb 14;16:1643. doi: 10.1038/s41467-025-56901-y (PMC11828953; doi:10.1038/s41467-025-56901-y)
Supplement: Supplementary file 5 — Reporting Summary [file 41467_2025_56901_MOESM5_ESM.pdf]

Corresponding author(s): Xin-Guang Zhu, Tiegang Lu, Chenzhi Liang, and Ming-Ju Amy Lyu

Last updated by author(s): Jan 10, 2025

Reporting Summary

Nature Portfolio wishes to improve the reproducibility of the work that we publish. This form provides structure for consistency and transparency in reporting. For further information on Nature Portfolio policies, see our Editorial Policies and the Editorial Policy Checklist.

Statistics

For all statistical analyses, confirm that the following items are present in the figure legend, table legend, main text, or Methods section.

n/a	Confirmed	
☐	☒	The exact sample size (*n*) for each experimental group/condition, given as a discrete number and unit of measurement
☐	☒	A statement on whether measurements were taken from distinct samples or whether the same sample was measured repeatedly
☐	☒	The statistical test(s) used AND whether they are one- or two-sided<br>*Only common tests should be described solely by name; describe more complex techniques in the Methods section.*
☒	☐	A description of all covariates tested
☐	☒	A description of any assumptions or corrections, such as tests of normality and adjustment for multiple comparisons
☐	☒	A full description of the statistical parameters including central tendency (e.g. means) or other basic estimates (e.g. regression coefficient) AND variation (e.g. standard deviation) or associated estimates of uncertainty (e.g. confidence intervals)
☒	☐	For null hypothesis testing, the test statistic (e.g. *F*, *t*, *r*) with confidence intervals, effect sizes, degrees of freedom and *P* value noted<br>*Give P values as exact values whenever suitable.*
☒	☐	For Bayesian analysis, information on the choice of priors and Markov chain Monte Carlo settings
☒	☐	For hierarchical and complex designs, identification of the appropriate level for tests and full reporting of outcomes
☒	☐	Estimates of effect sizes (e.g. Cohen's *d*, Pearson's *r*), indicating how they were calculated

Our web collection on statistics for biologists contains articles on many of the points above.

Software and code

Policy information about availability of computer code

Data collection	No software had been used for data collection.
Data analysis	Canu (v1.8), bwa v0.7.17, samtools (v1.11), Pilon (v1.22), HERA, HiC-Pro (v2.11.1), RepeatModeler (v.1.0.5), RepatMasker (v4.1.0), LTR_FINDER (v1.07) , LTRharvest (v1.5.10), Augustus (v2.4), GlimmerHMM (v3.0.4), GeneID (v1.4),Genscan, GeMoMa (v1.3.1), Hisat2 (v2.0.4), StringTie (v1.2.3), PASA (v2.0.2), EVidenceModeler (v1.1.1), GeneWise (v2.4.1), BUSCO (v3.0.2), STAR (v2.7.3a), bowtie2 (v2.3.4.3), BLASTP (v2.2.31+), Orthofinder (v2.3.11), MUSCLE (v3.8.31), RAxML (v7.9.3), pal2nal (v14), PAML (v4.9), RSEM (v1.3.3), fastp (v0.20.0), macs2 (v2.2.7.1), bedtools (v2.29.2), deeptools (v3.5.0), meme (v5.0.2), homer, riboWaltz (v2.0). R, Python. Perl. Specific parameters used during run-time are provided in the methods.

For manuscripts utilizing custom algorithms or software that are central to the research but not yet described in published literature, software must be made available to editors and reviewers. We strongly encourage code deposition in a community repository (e.g. GitHub). See the Nature Portfolio guidelines for submitting code & software for further information.

Data

Policy information about availability of data

All manuscripts must include a data availability statement. This statement should provide the following information, where applicable:

- Accession codes, unique identifiers, or web links for publicly available datasets
- A description of any restrictions on data availability
- For clinical datasets or third party data, please ensure that the statement adheres to our policy

The genome assemblies, gene annotations, proteomics data, and raw reads of transcriptome data, Ribo-seq data and ATAC-seq data are available at the China National GeneBank (CNGB) [https://db.cngb.org/codeplot/datasets/flaveria] with project ID CPN0003058. The genome assemblies, gene annotations, transcriptome data, and proteomics data are also available at figshare [https://doi.org/10.6084/m9.figshare.19918876.v4]. The genome assemblies are also available at the National Center for Biotechnology Information (NCBI) with accession numbers: SAMN14943594 for Frob [https://www.ncbi.nlm.nih.gov/biosample/SAMN14943594/], SAMN14943595 for Fson [https://www.ncbi.nlm.nih.gov/biosample/SAMN14943595], SAMN14943597 for Flin [https://www.ncbi.nlm.nih.gov/biosample/SAMN14943597], SAMN14943596 for Fram [https://www.ncbi.nlm.nih.gov/biosample/SAMN14943596], and SAMN14943598 for Ftri [https://www.ncbi.nlm.nih.gov/biosample/SAMN14943598]. The mass spectrometry proteomics data were submitted to the PRoteomics IDEntifications Database (PRIDE)116 with accession number PXD024720 [http://proteomecentral.proteomexchange.org/cgi/GetDataset?ID= PXD024720]. RNA-seq data of Flin were also submitted to Gene Expression Omnibus (GEO) in the NCBI database under accession number PRJNA827625 [https://www.ncbi.nlm.nih.gov/bioproject/PRJNA827625/] RNA-seq data of Frob, Fson, Fram, and Ftri were obtained from published data under project accession PRJNA600545 [https://www.ncbi.nlm.nih.gov/bioproject/PRJNA600545/]. Source data are provided with this paper.

Research involving human participants, their data, or biological material

Policy information about studies with human participants or human data. See also policy information about sex, gender (identity/presentation), and sexual orientation and race, ethnicity and racism.

Reporting on sex and gender	
Reporting on race, ethnicity, or other socially relevant groupings	
Population characteristics	
Recruitment	
Ethics oversight	

Note that full information on the approval of the study protocol must also be provided in the manuscript.

Field-specific reporting

Please select the one below that is the best fit for your research. If you are not sure, read the appropriate sections before making your selection.

☒ Life sciences ☐ Behavioural & social sciences ☐ Ecological, evolutionary & environmental sciences

For a reference copy of the document with all sections, see nature.com/documents/nr-reporting-summary-flat.pdf

Life sciences study design

All studies must disclose on these points even when the disclosure is negative.

Sample size	No statistical methods were used to predetermine sample size for genome sequencing as our samples were all from wild type.
Data exclusions	No data were excluded.
Replication	For RNA-seq, three biological replicates were performed, for proteomics, six biological replicates were performed, for ATAC-seq, two biological replicates were performed, for transient transcription assay, three biological replicates were used, and for qPCR, three biological replicates were used, for Ribo-seq, two biological replicates were performed.
Randomization	This is not relevant to our study, as all the analysis used in this study is based on well-establised methods.
Blinding	The investigators were blinded to groups allocation during data collection.

Behavioural & social sciences study design

All studies must disclose on these points even when the disclosure is negative.

Study description

Research sample

Sampling strategy

Data collection

Timing

Data exclusions

Non-participation

Randomization

Ecological, evolutionary & environmental sciences study design

All studies must disclose on these points even when the disclosure is negative.

Study description

Research sample

Sampling strategy

Data collection

Timing and spatial scale

Data exclusions

Reproducibility

Randomization

Blinding

Did the study involve field work? ☐ Yes ☐ No

Field work, collection and transport

Field conditions

Location

Access & import/export

Disturbance

Reporting for specific materials, systems and methods

We require information from authors about some types of materials, experimental systems and methods used in many studies. Here, indicate whether each material, system or method listed is relevant to your study. If you are not sure if a list item applies to your research, read the appropriate section before selecting a response.

Materials & experimental systems

n/a	Involved in the study
☐	☒ Antibodies
☒	☐ Eukaryotic cell lines
☒	☐ Palaeontology and archaeology
☒	☐ Animals and other organisms
☒	☐ Clinical data
☒	☐ Dual use research of concern
☐	☒ Plants

Methods

n/a	Involved in the study
☒	☐ ChIP-seq
☐	☒ Flow cytometry
☒	☐ MRI-based neuroimaging

Antibodies

Antibodies used

Western blots for PEPC, NADP-ME, and PPDK were performed using 0.6 g of fresh, fully expanded leaf tissue. Actin was used as a loading control. The antibody of PEPC and NADP-ME were custom-developed by Orizymes Biotechnologies Company (Shanghai). The antibody of PPDK was from Orizymes Biotechnologies Company (Shanghai) (catalog number: PAB07103)[http://www.phytoantibodies.com/a/ninanjie_shuangziyezhiwu_kangti/guanghezuoyong/C/2021/0728/172.html]. The antibody of Actin was from Yamei (Shanghai) (catalog number: LF208S)[http://www.epizyme.cn/pro_show.aspx?productid=1105]. For all experiments, these antibodies were diluted to a working concentration of 1:5000.

Validation

All the antibody were validated using Western Blot.

Eukaryotic cell lines

Policy information about cell lines and Sex and Gender in Research

Cell line source(s)

Authentication

Mycoplasma contamination

Commonly misidentified lines
(See ICLAC register)

Palaeontology and Archaeology

Specimen provenance

Specimen deposition

Dating methods

☐ Tick this box to confirm that the raw and calibrated dates are available in the paper or in Supplementary Information.

Ethics oversight

Note that full information on the approval of the study protocol must also be provided in the manuscript.

Animals and other research organisms

Policy information about studies involving animals; ARRIVE guidelines recommended for reporting animal research, and Sex and Gender in Research

Laboratory animals

Wild animals

Reporting on sex

Field-collected samples

Ethics oversight

Note that full information on the approval of the study protocol must also be provided in the manuscript.

Clinical data

Policy information about clinical studies

All manuscripts should comply with the ICMJE guidelines for publication of clinical research and a completed CONSORT checklist must be included with all submissions.

Clinical trial registration

Study protocol

Data collection

Outcomes

Dual use research of concern

Policy information about dual use research of concern

Hazards

Could the accidental, deliberate or reckless misuse of agents or technologies generated in the work, or the application of information presented in the manuscript, pose a threat to:

No	Yes	
☐	☐	Public health
☐	☐	National security
☐	☐	Crops and/or livestock
☐	☐	Ecosystems
☐	☐	Any other significant area

Experiments of concern

Does the work involve any of these experiments of concern:

No	Yes	
☐	☐	Demonstrate how to render a vaccine ineffective
☐	☐	Confer resistance to therapeutically useful antibiotics or antiviral agents
☐	☐	Enhance the virulence of a pathogen or render a nonpathogen virulent
☐	☐	Increase transmissibility of a pathogen
☐	☐	Alter the host range of a pathogen
☐	☐	Enable evasion of diagnostic/detection modalities
☐	☐	Enable the weaponization of a biological agent or toxin
☐	☐	Any other potentially harmful combination of experiments and agents

Plants

Seed stocks

F. robusta (Frob, C3) and F. ramosissima (Fram, C3-C4) were provided by Prof. Peter Westhoff (Heinrich Heine University, Germany). F. sonorensis (Fson, C3-C4), F. linearis (Flin, C3-C4), and F. trinervia (Ftri, C4) were obtained from Prof. Rowan F. Sage (University of Toronto, Canada).

Novel plant genotypes

Authentication

ChIP-seq

Data deposition

☐ Confirm that both raw and final processed data have been deposited in a public database such as GEO.

☐ Confirm that you have deposited or provided access to graph files (e.g. BED files) for the called peaks.

Data access links<br>*May remain private before publication.*	
Files in database submission	
Genome browser session<br>(e.g. UCSC)	

Methodology

Replicates	
Sequencing depth	
Antibodies	
Peak calling parameters	
Data quality	
Software	

Flow Cytometry

Plots

Confirm that:

☒ The axis labels state the marker and fluorochrome used (e.g. CD4-FITC).

☒ The axis scales are clearly visible. Include numbers along axes only for bottom left plot of group (a 'group' is an analysis of identical markers).

☒ All plots are contour plots with outliers or pseudocolor plots.

☒ A numerical value for number of cells or percentage (with statistics) is provided.

Methodology

Sample preparation	Nuclei were isolated from young leaves,using DAPI (NPE Analyzer, NPE 731085, USA) staining for 30 minutes.
Instrument	MD FACS Melody flow cytometer
Software	FACS data analyses were performed using Kaluza
Cell population abundance	More than 9000 cells were collected for each sample. Total nuclei populations were gated using relative fluorescence intensity: the proportions of nuclei with different ploidy levels were determined based on their relative fluorescence intensity: Tomato is a diploid (2N) as a reference, according to the peak position (Fig. S1 in Supplementary notes).
Gating strategy	Total nuclei populations were gated using PI intensity. In PI+ singles cells, the proportions of nuclei with different ploidy levels were determined based on their PI intensity (Fig. S1 in Supplementary notes).

☒ Tick this box to confirm that a figure exemplifying the gating strategy is provided in the Supplementary Information.

Magnetic resonance imaging

Experimental design

Design type	
Design specifications	
Behavioral performance measures	

Acquisition

Imaging type(s)

Field strength

Sequence & imaging parameters

Area of acquisition

Diffusion MRI ☐ Used ☐ Not used

Preprocessing

Preprocessing software

Normalization

Normalization template

Noise and artifact removal

Volume censoring

Statistical modeling & inference

Model type and settings

Effect(s) tested

Specify type of analysis: ☐ Whole brain ☐ ROI-based ☐ Both

Statistic type for inference
(See Eklund et al. 2016)

Correction

Models & analysis

n/a	Involved in the study
☐	☐ Functional and/or effective connectivity
☐	☐ Graph analysis
☐	☐ Multivariate modeling or predictive analysis

Functional and/or effective connectivity

Graph analysis

Multivariate modeling and predictive analysis
